# R-spondin family biology and emerging linkages to cancer

**DOI:** 10.1080/07853890.2023.2166981

**Published:** 2023-01-16

**Authors:** Zhimin He, Jialin Zhang, Jianzhong Ma, Lei Zhao, Xiaodong Jin, Hongbin Li

**Affiliations:** a School of Life Science and Engineering, Lanzhou University of Technology, Lanzhou, China; b Key Laboratory of Biotechnology and Bioengineering of State Ethnic Affairs Commission, Biomedical Research Center, Northwest Minzu University, Lanzhou, China; c Institute of Modern Physics, Chinese Academy of Sciences, Lanzhou, China

**Keywords:** R-spondin, WNT signaling, LGR4/5/6, tumor microenvironment, targeted therapy, biomarker

## Abstract

The R-spondin protein family comprises four members (RSPO1-4), which are agonists of the canonical Wnt/β-catenin pathway. Emerging evidence revealed that RSPOs should not only be viewed as agonists of the Wnt/β-catenin pathway but also as regulators for tumor development and progression. Aberrant expression of RSPOs is related to tumorigenesis and tumor development in multiple cancers and their expression of RSPOs has also been correlated with anticancer immune cell signatures. More importantly, the role of RSPOs as potential target therapies and their implication in cancer progressions has been studied in the preclinical and clinical settings. These findings highlight the possible therapeutic value of RSPOs in cancer medicine. However, the expression pattern, effects, and mechanisms of RSPO proteins in cancer remain elusive. Investigating the many roles of RSPOs is likely to expand and improve our understanding of the oncogenic mechanisms mediated by RSPOs. Here, we reviewed the recent advances in the functions and underlying molecular mechanisms of RSPOs in tumor development, cancer microenvironment regulation, and immunity, and discussed the therapeutic potential of targeting RSPOs for cancer treatment. In addition, we also explored the biological feature and clinical relevance of RSPOs in cancer mutagenesis, transcriptional regulation, and immune correlation by bioinformatics analysis.

**KEY MESSAGES**
- Aberrant expressions of RSPOs are detected in various human malignancies and are always correlated with oncogenesis.
- Although extensive studies of RSPOs have been conducted, their precise molecular mechanism remains poorly understood.
- Bioinformatic analysis revealed that RSPOs may play a part in the development of the immune composition of the tumor microenvironment.

## Introduction

R-spondins (roof plate-specific spondins; RSPOs) are represented by four secreted glycoproteins (RSPO1-4) with a sequence similarity of 40 ∼ 60%. The common structure of RSPO1-4 contains four major functional regions: two adjacent cysteine-rich furin-like domains (CR), a thrombospondin type 1 repeat domain (TSP1) and a basic amino acid-rich domain (BR) with varying length at the C-terminus [1].

RSPOs stimulate and synergize the canonical Wnt/β-catenin pathway, which regulates cell development and proliferation, as well as disease pathogenesis. RSPOs cannot directly initiate Wnt signaling, but act as a ligand for LGR4-6 receptors to activate the canonical Wnt/β-catenin pathway and potentiate reactions of low-dose Wnt proteins [2]. Upon binding to LGR4-6, LGR4-6 binds to phosphorylated LRP6 and Frizzled (FZD) receptors, which are activated by extracellular Wnt receptors. This induces the Wnt/β-catenin pathway and increases the expression of target genes. Additionally, they are also capable of inhibiting the activation of ZNRF3, a crucial regulator of the Wnt pathway, to modulate the Wnt/β-catenin pathway [3,4]. Furthermore, RSPOs also have a regulatory role in the non-canonical pathway and other signaling pathways [5], such as MAPK [6], BMP [7], and TGF-β [8]. The aberrant expression and gene fusions of RSPOs have been observed in a variety of tumors.

There is growing evidence implicating RSPOs in cancer development, and the expression of RSPOs is associated with a favorable prognosis. Bioinformatic analyses such as gene enrichment found that RSPOs are widely involved in immune regulation. For example, the latest study reported that RSPO increased tumor sensitivity to anti-PD-1 therapy, highlighting an unprecedented treatment approach [9,10]. Immune checkpoint blockade has been widely used in the clinical treatment of tumors, but the lack of tumor antigens and the failure to effectively initiate adaptive immunity led to poor efficacy of immunotherapy. Despite the insufficient evidence of implementing RSPOs with immunotherapy, RSPOs have great potential in tumor immunotherapy. In this review, we presented a summary of our understanding of the RSPO family and integrated various potential aspects of RSPO proteins, from structure to diverse cellular functions, for cancer therapy.

## Structural features of the RSPOs

**Figure 1.**
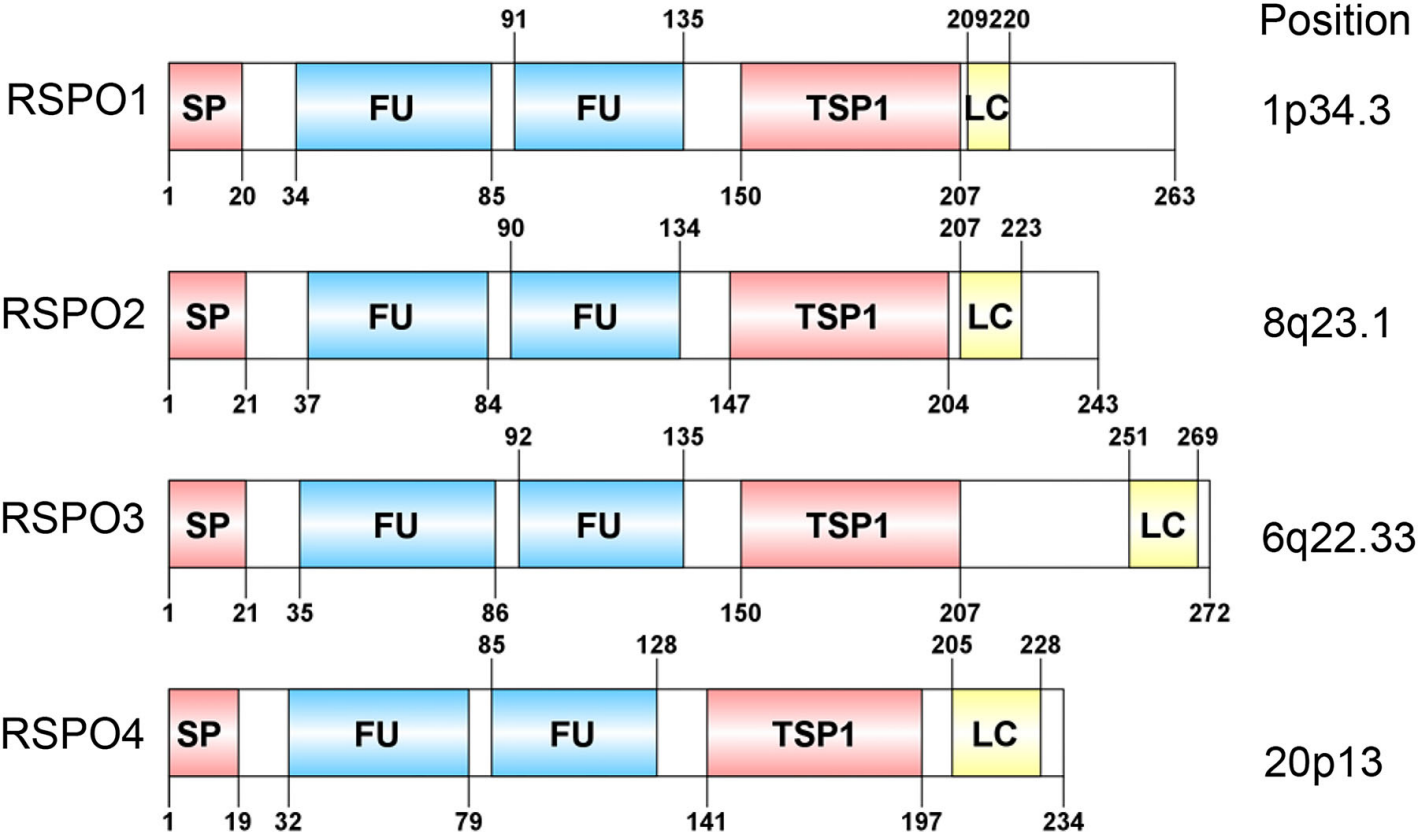
Domain structure and chromosomal localization of human RSPOs. The lengths of RSPO1-4 are 263, 243, 292, and 234 amino acids, respectively. SP: signal peptide; FU: The furin-like cysteine-rich region; TSP1: thrombospondin type 1 repeats; LC: low complexity region.

The RSPO protein family consists of four members, RSPO1-4, with sequence similarity of 40 ∼ 60% and a common structure. Thus far, all reported RSPO proteins contain four major functional regions: two adjacent cysteine-rich furin-like domains (FU1-FU2), a TSP1 domain, and a BR domain with varying length at the C-terminus. As a typical secretory protein, the RSPO protein has a signal peptide with a length of 20 ∼ 25 amino acids at its N-terminus. After the signal peptide, there are two adjacent furin-like domains, which are rich in cysteine and are the most conserved regions in the RSPO protein structure (Figure 1).

**Figure 2.**
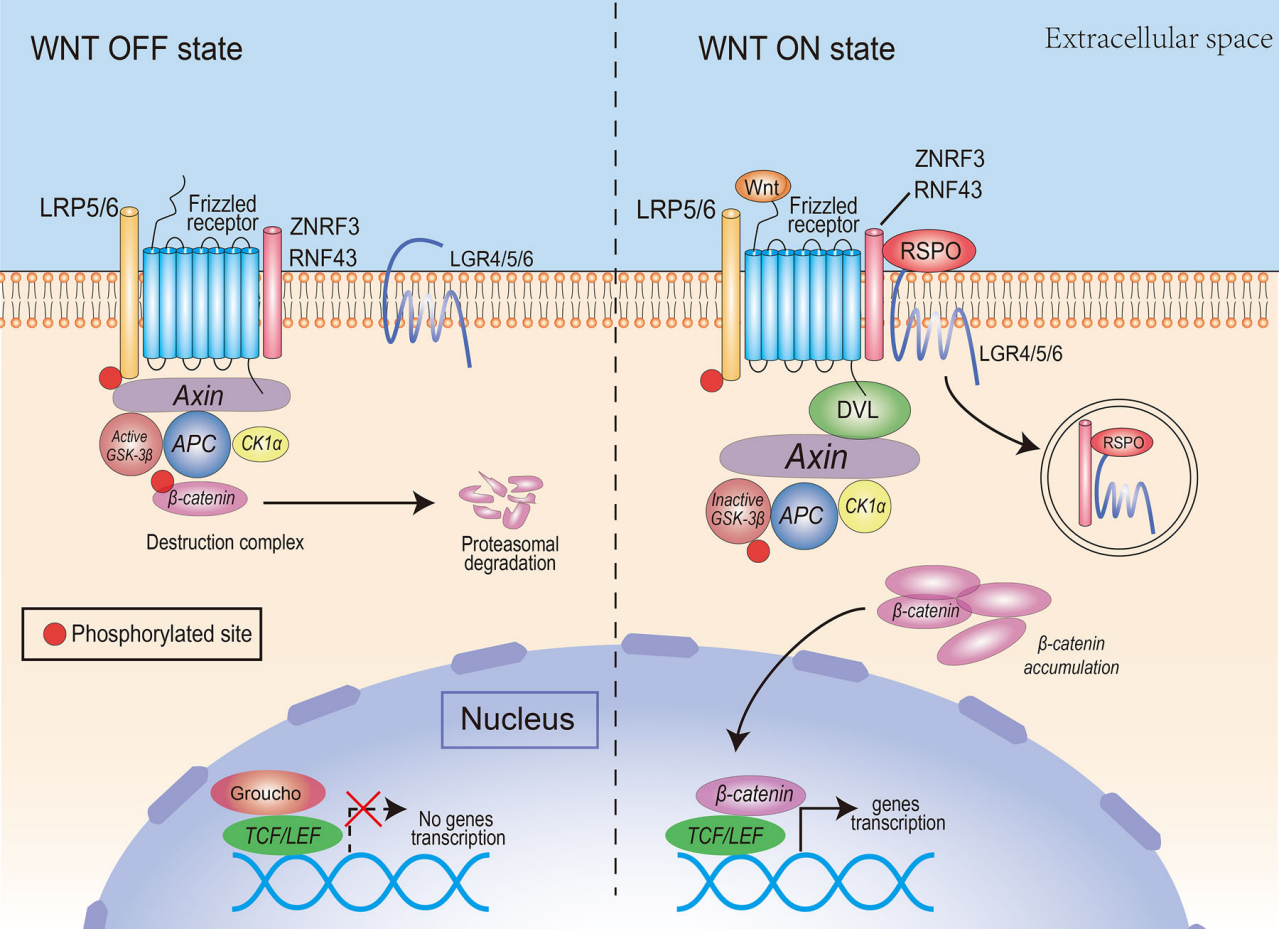
The canonical Wnt pathway and the potentiating effect of RSPOs. Canonical Wnt ligands induce the activation of DVL and inhibit the activity of the destruction complex, leading to β-catenin accumulation, nuclear translocation, and transcription of Wnt target genes. RSPOs enhance the canonical Wnt/β-catenin signaling pathway by eliminating the negative regulator ZNRF3/RNF43, thus enhancing the availability of membrane Wnt receptors and Wnt ligand-mediated pathway activation. In the absence of canonical Wnt ligands, the destruction complex induces β-catenin phosphorylation. The phosphorylated β-catenin is degraded by the cytoplasmic proteasome.

The main function of RSPOs is to synergize the canonical Wnt/β-catenin signaling pathway. The operation of the canonical Wnt/β-catenin signaling pathway depends on the activation of the upstream Wnt/FZD/LRP complex (Figure 2). In the case where there is no basal state for Wnt ligand stimulation, β-catenin in the cytoplasm interacts with APC, Axin, GSK-3β, and CK1α to develop a ‘destruction complex’, which degrades β-catenin via proteasomes. At the same time, TCF/LEF transcription factors bind to a variety of transcription suppressor proteins to inhibit Wnt signaling [11].When the Wnt ligand connects and activates FZD and LRP5/6 co-receptors on the membrane, it inhibits GSK-3β phosphorylation of β-catenin in the destruction complex. Unphosphorylated β-catenin accumulates in the cytoplasm and is transported to the nucleus, where it forms transcriptional activation complexes with TCF/LEF factors, which promote transcription of specific downstream target genes [12].

It is well established that the furin-like domain plays a decisive role in activating the canonical Wnt/β-catenin signaling pathway via RSPO proteins. Reports from Nam et al. implicated that RSPO could bind to LRP6, FZD, and Wnt1. However, in the absence of two furin-like domains, RSPO is unable to bind to LRP6, FZD, and Wnt1, affecting the activation of the Wnt/β-catenin signaling pathway [13]. Additionally, RSPOs could be co-activated by LRP6 and Wnt1/β-catenin. After the deletion of two furin-like domains, the synergistic effect of RSPO with LRP6 and Wnt1 was significantly inhibited. Similar results were reported previously, indicating that the function of the furin-like domain in RSPOs is essential for activating β-catenin signaling [14]. The function of different RSPO proteins is determined via their two furin-like domains. Li et al. proved that there were five free cysteine residues in a purified peptide embodying both furin-like repeats of RSPO2; three in furin repeat 1 and two in furin repeat 2 [15].

The TSP1 domain is involved in many signaling pathways that are critical to vascular physiology and disease occurrence. Ayadi et al. conducted three-dimensional structure simulation experiments on the TSP1 domain of human RSPO4 and found that the presence of basic amino acids in this domain formed a large positively charged region, in which tryptophan and arginine were arranged to form a groove-like structure [16]. A similar structure exists in the TSP1 domain of F-spondins and thrombospondin-related anonymous protein (TRAP), but this structure itself does not have high binding efficiency to heparin, and the C-terminus of RSPO4 has a cluster of basic amino acids that matches the heparin sequence. Therefore, the TSP1 and C-terminus of RSPO4 are likely to form a continuous surface that can bind to heparin.

The RSPO family requires the activity of Wnt ligands and LRP6 to amplify the signal conduction of Wnt3A, Wnt1, and Wnt7A, indicating that RSPO proteins are common modulators of canonical Wnt/β-catenin signaling [14]. Han et al. suggested that RSPOs produced a marked effect through the Wnt/β-catenin signaling pathway [17].

Recent studies have also discovered some novel molecular mechanisms of RSPOs. By way of illustration, Hao et al. demonstrated that the cell-surface transmembrane E3 ubiquitin ligase zinc and ring finger 3 (ZNRF3) and its homolog ring finger 43 (RNF43) had negative roles in Wnt signaling. ZNRF3 is connected with the Wnt receptor complex, and the Wnt signaling is suppressed due to the turnover of FZD and LRP6. Inhibiting ZNRF3 not only enhances Wnt/β-catenin signaling but also interrupts Wnt/planar cell polarity (PCP) signaling [18]. Kazanskaya et al. found that the effect of RSPO2 on the Wnt/β-catenin signaling pathway occurred in DSH/DVL or an upstream level. Besides, there is no combination of RSPO2 and LRP6 or FZD, which speculates that there may be other role factors. The study found that the *RSPO* gene was commonly expressed with Wnt, and the expression is formed in Wnt → *RSPO* → Wnt [19].

Although RSPO family proteins can increase the activity of the canonical Wnt/β-catenin signaling pathway, their regulatory capabilities differ due to structural differences. Based on the evidence, a new study has reported that RSPO2 and RSPO3 can enhance Wnt/β-catenin signaling with the deletions of LGR4-6. The need for LGR4-6 is determined by the interplay between RSPOs and the ZNRF3/RNF43 E3 ubiquitin ligases [20]. Lee et al. assessed the interrelations between the expression of the RSPOs and LGRs using the single-sample GSEA method and concluded that RSPO1 and LGR6 were coordinately expressed in high-grade serous ovarian cancer (HGSOC) and two normal tissues, and their expression was associated with Wnt pathways [21].

Gene fusions have been considered to be driver mutations in tumors, and the findings of numerous studies have aided us in developing a better knowledge of the carcinogenic process [22]. Gene fusions involving *RSPOs* have been proven to induce the genesis of Wnt-dependent cancers in a variety of malignancies. Based on recent research reports, the authors identified five previously unreported RSPO fusion events (e.g. *IFNGR1*-*RSPO3*) [23]. Seshagiri et al. used RNA-SEQ to analyze 70 pairs of colon cancer tumors and their adjacent non-cancer tissues and identified a plurality of fusion transcripts involving *RSPO2* and *RSPO3*, where the two transcriptions appeared together in patients with colon cancer in colon cancer. After fusion with EIF3E or PTPRK exon 1, the expression of *RSPO2* or *RSPO3* increased, which activated the Wnt pathway accordingly [24]. Zhang et al. studied the regulation of specific candidate fusion genes in the HCT116 colon cancer cell line by utilizing the brother of the regulator of imprinted sites (BORIS). It was found that BORIS inhibited the expressions of *EIF3E*, *RSPO2*, *PTPRK*, *RSPO3*, *TADA2A,* and *CD4* in HCT116 cells and the expression of fusion transcripts [25]. A more detailed understanding of fusion proteins in terms of their regulatory mechanisms and the downstream biological effects in cellular processes can facilitate the development of new therapeutic methods in tumor treatment.

## Functions of the RSPOs

### Reproductive system development

**Figure 3.**
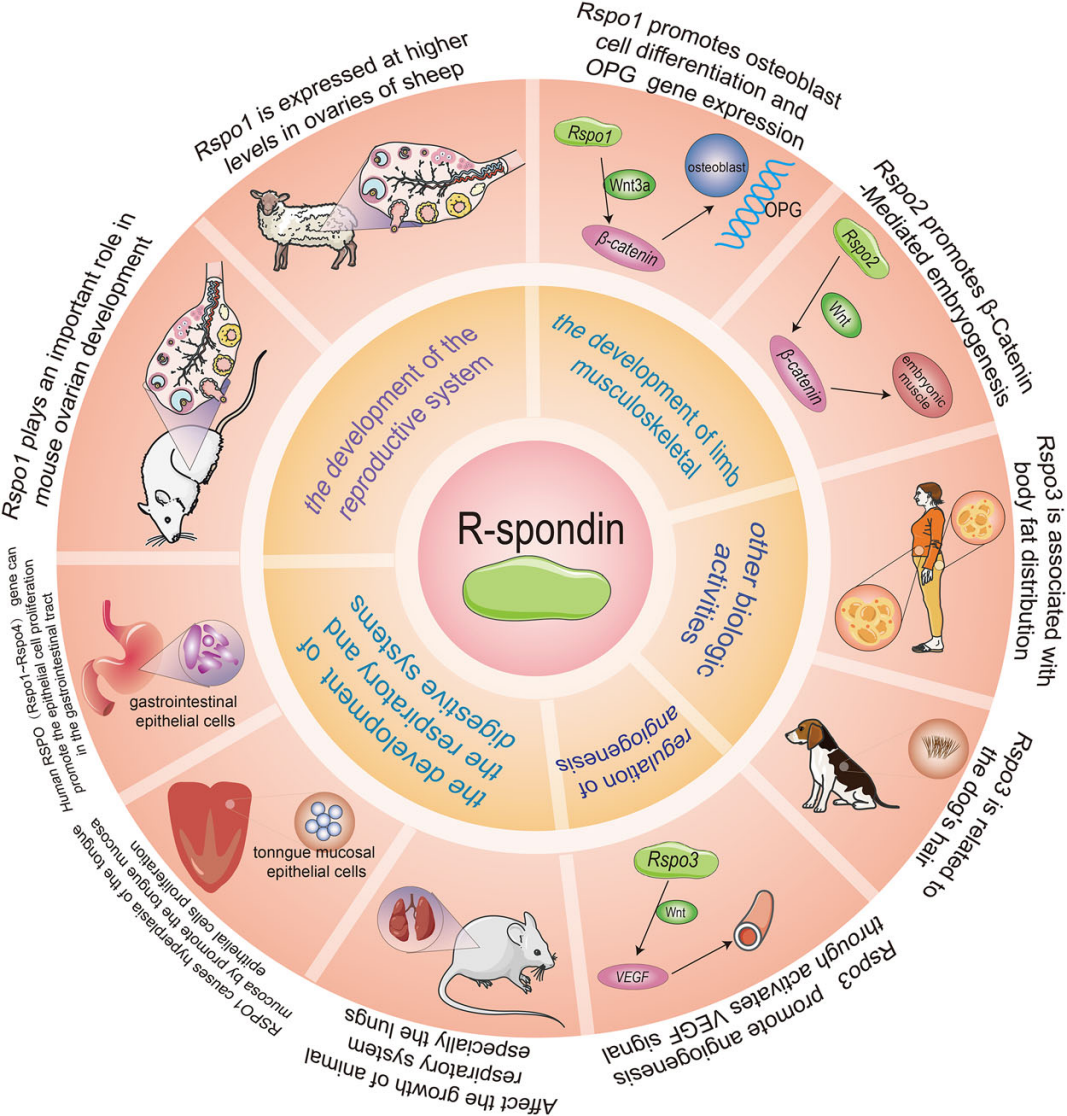
Functions of the RSPO family *in vivo*. The RSPO family has a wide range of biological functions, including reproductive system development, limb musculoskeletal development, angiogenesis, respiratory and digestive system development, and other biological activities.

The RSPO family has a huge impact on the development of animal reproductive systems (Figure 3). Smith et al. suggested that the expression of the gonad *RSPO1* gene was upregulated in the embryonic gonads of each species at the beginning of sex differentiation [26]. In chicken embryos, the expression of RSPO1 increased during female ovarian differentiation, which was consistent with female-specific *Foxl2* gene activation and estrogen synthesis. To study fish sex determination, the research of the *RSPO1* gene of zebrafish by Zhang et al. indicated that the expression of RSPO1 in the ovary was stronger than that in the testis, clarifying a differential RSPO1 expression between various sex organs [27]. Moreover, the RSPO1 signal in the breast epithelium is also necessary for the normal development of the breast. Tomizuka et al. established *Rspo1*-null (*Rspo1^-/-^*) mice as models, then finally observed that *Rspo1^-/-^* female mice exhibited male characteristics, including pseudosexuality in the reproductive ducts and fetuses [28]. It follows that RSPO1 may accelerate gonadal differentiation, inhibit the male differentiation program, and maintain the survival of oocytes.

RSPO2 can also regulate the development of the female reproductive system. Choi et al. knocked out the *Nobox* gene, which plays a significant role in oocyte development, in mice, but RSPO2 was significantly expressed in early oogenesis, indicating that RSPO2 may regulate the development of oocytes downstream of *Nobox* [29]. De Cian et al. found that RSPO2 expression in both human and mouse ovaries was limited to the oocyte of developing follicles from the beginning of follicular growth [30]. RSPO2 plays an irreplaceable role in maintaining female reproductive capacity. Nam et al. constructed a mouse model that blocked RSPO2 expression. In contrast to the normal performance of *Rspo2* (+/−) male mice, *Rspo2* (+/−) female mice started to lose their fertility from 4 months of age [31]. In the study of goat gonadal development, Kocer et al. discovered that RSPO1 and RSPO2 displayed a sex-dependent expression pattern, and their expression was higher in the ovary [32]. Unlike RSPO1, the expression pattern of goat RSPO2 was significantly affected by polled intersex syndrome (PIS) mutations.

### Angiogenesis

Wnt/β-catenin signaling is indispensable for the process of angiogenesis. RSPO3 could activate Wnt/β-catenin signaling and have crucial effects on angiogenesis. Aoki et al. constructed mice models carrying a mutant *Rspo3* allele and discovered that the homozygous mutant mice died from the damage to the labyrinthine layer of the placenta at about embryonic day 10, suggesting that RSPO3 may affect blood vessel generation and development [33]. Kazanskaya et al. revealed that the knockdown of RSPO3 in *Xenopus* embryos caused defects in the vascular system, which implied that RSPO3 regulation between angioblast and blood cell specification was essential. Similarly, the targeted destruction of RSPO3 in mice resulted in vascular defects and death. In addition, RSPO signaling can boost proliferation and angiogenesis in human endothelial cells [34]. Caruso et al. proved that under culture conditions, RSPO1 alone was sufficient to promote testicular angiogenesis [35]. These results identified RSPO3 as an original, vital, and evolutionarily conserved angiogenic factor.

### Skeletal development

Skeletal development is regulated by the Wnt/β-catenin signaling pathway. Wnt/β-catenin signal controls the development of animal bones by promoting the development, differentiation, and activation of osteoprotegerin (OPG) gene expression [36]. Bell et al. demonstrated that RSPO2, which was mediated by LRP6 through the canonical Wnt/β-catenin signaling pathway, generated normal differentiation and growth of the respiratory tract and limbs [37]. Hankenson et al. reflected that RSPOs were highly expressed in developing and postnatal skeletal tissues of mice. RSPOs promote osteoblast differentiation, and the lack of RSPO2 leads to bone developmental defects [38]. Han et al. investigated that RSPOs were Wnt/β-catenin signaling pathway activators in the development of skeletal muscle cells. Particularly, in the shortage of the *Rspo2* gene, MYF5 in the developing limbs of mouse embryos exhibited a reduced expression [17]. Furthermore, Knight et al. worked with *Rspo2* conditional knockout (Rspo2^floxed^) mice with the Osteocalcin-Cre mouse line (Ocn- Cre + Rspo2^f/f^), which sequentially influenced the expression of RSPO2 in osteoblast-lineage cells. They observed lower overall body size and bone mass of Ocn- Cre + Rspo2^f/f^ mice as compared with the control littermates. These results indicated that *Rspo2* gene knockout had a significant effect on skeletal growth in mice [39]. Doherty et al. pointed out that LGR, also known as Wnt-related receptor, interacted with RSPO ligands and played a prominent role in bone and dental biology [40].

### Digestive systems and respiratory system

A certain amount of evidence revealed that RSPOs play a crucial role in the development of both the digestive and respiratory systems. For example, recent evidence suggests that recombinant human RSPO1 (rhRSPO1) treatment improved the culture of mouse intestinal organoid units, and increased their size and survival rate *in vitro*. hRSPO1 is expressed in intestinal endocrine and epithelial cells of assorted tissues, and also displayed effectiveness in the model of chemotherapy-induced intestinal mucositis [41]. Zhao et al. injected the recombinant RSPO1 protein *in vitro* into TOPGAL mice and tested the tongue, stomach, lung, liver, small intestine, large intestine, skin, and gonads. The results specified that the tongue exhibited an RSPO1-specific response. RSPO1 enhanced the proliferation of the basal epithelial cells in the tongue mucosa via Wnt/β-catenin signaling [42]. Interestingly, another study also reported that the lack of RSPO in the culture medium could not generate taste bud cells *in vitro*, suggesting that RSPO is necessary for the generation of differentiated taste cells. Therefore, it is proposed that RSPO can maintain the steady state of taste tissues.

LGR5 is RSPOs receptor that regulate both Wnt/β-catenin and Wnt/PCP signaling. Harnack et al. demonstrated that RSPO3 was a key element for epithelial restoration in differentiated cells *via* Wnt signaling [43]. Axin2 is a marker of the Wnt signaling pathway, and RSPO3 participates in the expression process of both Axin2 and LGR5. Sigal  et al. proved that RSPO3 knockout resulted in the hypercolonization of *Helicobacter pylori* (*H. pylori*) in the gastric glands. The deficiency of Lgr5+ cells yielded similar results, and RSPO3 overexpression in the stroma cleared *H. pylori* from the gastric glands. Accordingly, RSPO3/LGR5 can regulate the regeneration of the digestive tract mucosa and improve their defensive ability [44]. Yan et al. identified that Wnt and RSPO ligands play a coordinating role in intestinal crypt stem cells. Wnt protein maintained the expression of the RSPO receptor and induced RSPO ligands to activate the self-renewal of LGR5 [45]. A report from Greicius et al. indicated that RSPO3 was expressed in the pericryptal myofibroblasts of the lamina propria, and could significantly trigger canonical Wnt/β-catenin signaling and organoid proliferation. Dissolution of RSPO3 *in vitro* led to a significant reduction in organoid growth, while exogenous RSPO3 protein restored this phenotype [46]. On the contrary, Liu et al. confirmed that hepatocytes were less sensitive to hypoxia/reoxygenation (H/R) injury after being pretreated with RSPO3. Knockdown of LGR4 with shRNA could weaken the protective effects of RSPO3. Hence, RSPO3/LGR4 protects the hepatocytes from H/R injury by stimulating β-catenin [47].

In the respiratory system, Nam et al. found that *Rspo2*(−/−) homozygous mutant mice had lung development defects [31]. Bell et al. interbred the *Rspo2* (*Rspo2^Tg^*) and *Lrp6^–^* alleles in mice models and discovered that the mice developed lung hypoplasia and damaged the tracheobronchial ring and laryngeal structures [37]. Wu et al. used ONCOMINE analysis to illustrate the expression levels of RSPO1, 2, and 3 mRNAs in lung cancer tissues and found that they were notably lower than those in normal samples [48]. Munguía-Reyes et al. found that upregulated RSPO2 and its receptor LGR6 occurred in fibroblasts and epithelial cells of patients with idiopathic pulmonary fibrosis (IPF) in the lungs [49].

## RSPOs expression in cancer

A growing body of evidence revealed that the expression of RSPOs in all manner of tumors tends to be abnormal. For instance, the expressions of RSPO1, RSPO2, and RSPO3 were significantly reduced in lung cancer patients as compared with normal tissues [48]. Coussy et al. also detected RSPO2 and RSPO4 overexpression levels in breast cancers, particularly in triple-negative breast cancers [50].

**Figure 4.**
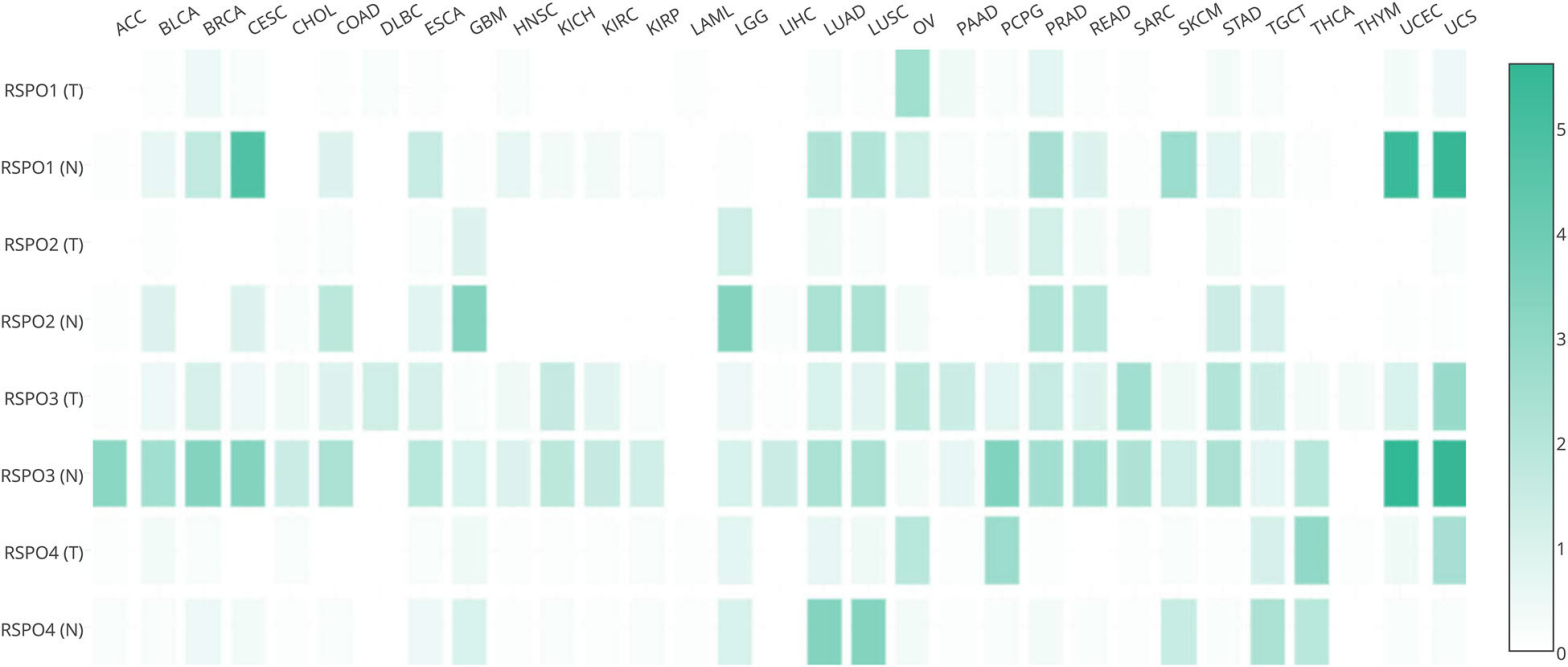
Expression matrix plots of RSPOs in multiple tumors. The density of color in each block represents the median expression value of a gene in a given tissue, normalized by the maximum median expression value across all blocks.

We analyzed the abnormal expression of RSPOs in different tumors through the GEPIA database (http://gepia.cancer-pku.cn/). As seen in Figure 4, RSPO1 expression was upregulated in OV and downregulated in CESC, SKCM, UCEC, and UCS. The expression of RSPO2 was downregulated in GBM, LGG, and LUAD. RSPO3 was upregulated in DLBC and downregulated in a variety of tumors, including ACC, BRCA, CESC, PCPG, UCEC, and UCS. RSPO4 was significantly downregulated in lung cancer.

To explore the transcription factor of RSPOs, we selected 2000 bp promoter sequences of the *Rspo1-4* gene from the NCBI database (https://www.ncbi.nlm.nih.gov/) and predicted their transcription factors (homo sapiens only) through UCSC-JASPAR (http://genome.ucsc.edu/; https://jaspar.genereg.net/). The track scores were over 600 (track score ≥131, *p*-value <0.05; track score ≥600, *p*-value <10^−6^). We utilized NCBI to study the tissue specificity of the predicted transcription factors and selected the tissues with the highest expression of human transcription factors. We predicted the binding sites of *Rspo1-4* genes and potential transcription factors through JASPAR and displayed the binding sites with the highest relative score, including the bases and positions (Table 1).

**Table 1.** TFs predictions of *Rspos* promoters.

Gene	TF^a^	Species	Strand	Highest expression tissue	Predicted binding site^b^
*Rspo1*	MYF5	Homo sapiens	±	–	GAACAGCTGTTT 1203-1214
	MYF6	Homo sapiens	±	Prostate	AACAGCTGTT 1204-1213
	MSC	Homo sapiens	±	Placenta	AACAGCTGTT 1204-1213
	ZNF460	Homo sapiens	–	Adrenal	GCCTCAGCCTCCCAAA 40-55
	POU3F1	Homo sapiens	–	–	ATATGCAAATTT 126-137
	POU3F2	Homo sapiens	–	–	ATATGCAAATTT 126-137
	POU2F3	Homo sapiens	–	Skin	CATATGCAAATTT 125-137
	RBPJ	Homo sapiens	+	–	TATGGGAAAA 260-269
*Rspo2*	GATA2	Homo sapiens	–	Prostate	TTCTTATCTTT 1165-1175
	TRPS1	Homo sapiens	–	Esophagus	TTTCTTATCTAT 821-832
	GATA6	Homo sapiens	–	Stomach	TTTTCTTATCTAT 820-832
	ZNF354A	Homo sapiens	+	Testis	–
*Rspo3*	PRDM9	Homo sapiens	–	Testis	GGAAGGAAGGAAGGAAGGAAAGAA 1190-1213
	PRDM1	Homo sapiens	–	Endometrium	CTCTTTCCCTC 1801-1811
	PATZ1	Homo sapiens	–	Ovary	ATGGGGTGGGGG 1467-1478
	KLF15	Homo sapiens	+	Fat	CCCCCGCCCCC 1883-1893
	GATA2	Homo sapiens	–	Prostate	GGCTTATGTTA 514-524
	POU1F1	Homo sapiens	+	Gall bladder	ACCTAATTTACATAAA 257-272
	POU2F2	Homo sapiens	–	Lymph node	TTCATGTAAATTTA 244-257
	POU3F1	Homo sapiens	–	–	TCATGTAAATTT 245-256
*Rspo4*	ZNF460	Homo sapiens	+	Adrenal	–
	ZNF768	Homo sapiens	–	Testis	TCACTTGGCCTCTCTGAG 940-957
	ZNF652	Homo sapiens	–	Skin	GAAAGGGTTAAA 1168-1179
	ZNF341	Homo sapiens	–	Testis	AGGAACAGCCAG 1555-1566
	PATZ1	Homo sapiens	+	Ovary	GAGGGGTGGGGC 32-43
	MAZ	Homo sapiens	–	Bone marrow	GCCCCCTCCCC 1892-1902
	KLF15	Homo sapiens	–	Fat	CCCCCGCCCCC 1887-1897
	E2F6	Homo sapiens	–	Testis	GGGGGCGGGAAAG 1819-1831

^a^Highest expression tissue: the results came from NCBI. According to the RNA sequences of 95 human individual tissue samples representing 27 different tissues, the tissue specificity of all protein coding genes was determined, and the tissue with the highest expression was selected. Where ‘–’ indicated no data. ^b^Predicted binding site: only the predicted binding sites with the highest relative score were listed. Where ‘–’ indicated that the relative score was lower than 0.8 or the prediction results were all antisense chains.

**Figure 5.**
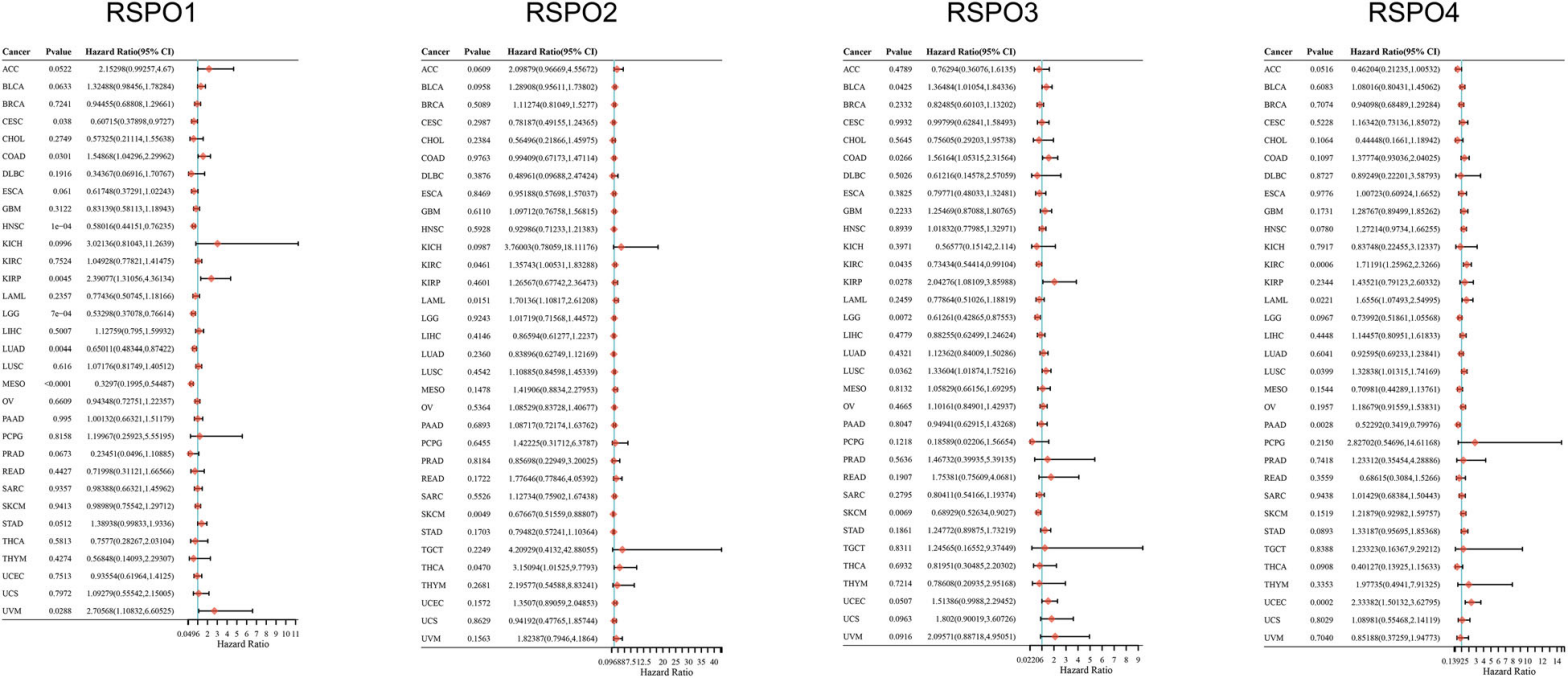
Univariate cox analysis results of RSPOs in multiple tumors. Forest plot: The *p*-value, risk coefficient (HR), and confidence interval of RSPOs in multiple tumors are analyzed by univariate cox regression.

RSPOs expression is altered in tumors, implying the outstanding effect of RSPOs in tumor progression. We utilized the univariate Cox regression analysis to determine the univariate predictors. RNA-sequencing expression profiles and corresponding clinical information for RSPOs were downloaded from the TCGA dataset (https://portal.gdc.com). Using the univariate cox regression analysis and the ‘forestplot’ R package, the *p*-value, hazard ratio (HR) and 95% CI of each variable were determined. The univariate Cox was proportional to the hazards regression analysis (HR ≠ 1, *p* < 0.05) (Figure 5) and RSPOs were linked to poor prognosis in a variety of tumors (HR > 1, higher expression of RSPOs suggests poor prognosis of patients; HR < 1, lower expression of RSPOs suggests poor prognosis of patients).

## Mutation of RSPOs in cancer

Gene mutations have been a hot topic in tumor research. It can occur at any stage of development, usually during DNA replication. The mutation is related to DNA replication, damage, and repair, as well as cancer and aging. It is also one of the important factors in biological evolution. Therefore, the study of gene mutation has extensive biological significance in addition to its theoretical significance.

**Figure 6.**
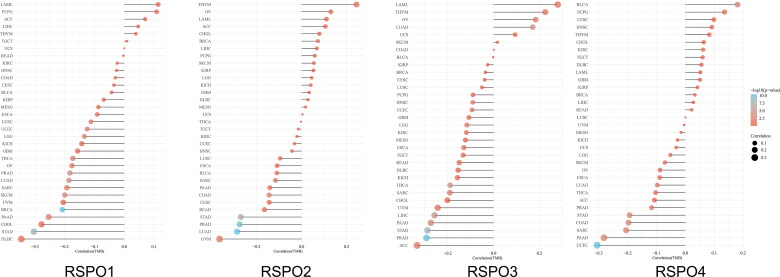
Spearman correlation analysis of TMB and RSPOs expression. The abscissa represents the correlation coefficient between genes and TMB, the ordinate represents different tumors. The size of the dots represents the size of the correlation coefficient, and different colors represent the significance of the *p*-value. The deeper the blue color, the smaller the *p*-value.

Genetic mutations of RSPOs were analyzed through the cBioPortal online tool (http://www.cbioportal.org/), and the results are provided in Table 2. RSPO3 is the most mutated gene in tumors, and the mutation sites are mainly concentrated at 100-250 amino acid sites. Tumor mutational burden (TMB) is defined as the total number of somatic gene coding errors, base substitutions, gene insertion, or deletion errors detected per million bases. TMB is the latest marker for evaluating the therapeutic efficacy of PD-1 antibody, and its efficacy has been proven in the treatment of colorectal cancer with mismatch repair defects [51,52]. We have analyzed the spearman correlation of TMB and RSPOs expression using bioinformatics. In Figure 6, RSPO1 expression was negatively correlated with TMB in a variety of tumors, especially in PAAD, CHOL, STAD, and DLBC. Likewise, RSPO3 was positively correlated with TMB in LAML, THYM, OV, and other tumors. A recent study also found that gene fusions involving R-spondin (RSPOfp) and RNF43 mutations induced WNT-dependent tumor initiation in colorectal cancer [23]. The biological significance of these mutations deserve further experimental evaluation.

**Table 2.** Genetic mutations of *Rspos.*

Gene	Study of origin	Sample ID	Cancer type detailed	Protein change	Mutation type	Variant type	Copy #	COSMIC	Allele Freq (T)^a^	# Mut in sample^b^
*Rspo1*	Metastatic solid cancers (UMich, Nature 2017)	MO_1209	Clear cell carcinoma of the lung	R66S	Missense_Mutation	SNP			0.17	575
	Metastatic solid cancers (UMich, Nature 2017)	MO_1233	Adenoid cystic carcinoma	A182T	Missense_Mutation	SNP			0.94	22
	Metastatic solid cancers (UMich, Nature 2017)	MO_1307	Cutaneous squamous cell carcinoma	G23R	Missense_Mutation	SNP			0.19	3053
	MSS mixed solid tumors (Broad/Dana-Farber, Nat Genet 2018)	CR9699_T	Cutaneous melanoma	C97_E100del	In_Frame_Del	DEL			0.31	1291
	MSS mixed solid tumors (Broad/Dana-Farber, Nat Genet 2018)	HNSCC-296 -Tumor-SM-CLFOB	Oropharyn × squamous cell carcinoma	C56S	Missense_Mutation	SNP			0.04	423
	MSS mixed solid tumors (Broad/Dana-Farber, Nat Genet 2018)	MEL-IPI_Pat139 -Tumor-SM-5VWJH	Cutaneous melanoma	E174V	Missense_Mutation	SNP			0.39	1387
	MSS mixed solid tumors (Broad/Dana-Farber, Nat Genet 2018)	MEL-IPI_Pat21 -Tumor-SM-4DK1H	Cutaneous melanoma	P183S	Missense_Mutation	SNP			0.08	1428
	Pan-cancer analysis of whole genomes (ICGC/TCGA, Nature 2020)	SP16958	Colorectal adenocarcinoma	R2Q	Missense_Mutation	SNP	Diploid	2	0.41	146
	Pan-cancer analysis of whole genomes (ICGC/TCGA, Nature 2020)	SP56730	Lung squamous cell carcinoma	N67K	Missense_Mutation	SNP	Diploid	1	0.42	218
	Pan-cancer analysis of whole genomes (ICGC/TCGA, Nature 2020)	SP82900	Melanoma	P145S	Missense_Mutation	SNP	Diploid	1	0.5	1246
	Pan-cancer analysis of whole genomes (ICGC/TCGA, Nature 2020)	SP124401	Melanoma	E195K	Missense_Mutation	SNP	Diploid		0.65	2446
	Pan-cancer analysis of whole genomes (ICGC/TCGA, Nature 2020)	SP124439	Melanoma	E195K	Missense_Mutation	SNP	Gain		0.5	1504
	Pan-cancer analysis of whole genomes (ICGC/TCGA, Nature 2020)	SP124423	Melanoma	S33R	Missense_Mutation	SNP	Diploid		0.14	155
*Rspo2*	MSK MetTropism (MSK, Cell 2021)	P-0036478-T01-IM6	Colorectal adenocarcinoma	RSPO2-RAD21 fusion - Archer	fusion	FUSION			0.11	6
	MSK MetTropism (MSK, Cell 2021)	P-0045582-T01-IM6	Colon adenocarcinoma	RSPO2-MECOM fusion - Archer	fusion	FUSION			0.29	5
	Metastatic solid cancers (UMich, Nature 2017)	MO_1087	Colon adenocarcinoma	R4C	Missense_Mutation	SNP		1	0.13	159
	Metastatic solid cancers (UMich, Nature 2017)	MO_1107	Breast invasive ductal carcinoma	D239N	Missense_Mutation	SNP			0.06	88
	Metastatic solid cancers (UMich, Nature 2017)	MO_1141	Squamous cell carcinoma, NOS	S103C	Missense_Mutation	SNP			0.24	311
	Metastatic solid cancers (UMich, Nature 2017)	MO_1266	Breast invasive ductal carcinoma	R158T	Missense_Mutation	SNP			0.21	438
	Metastatic solid cancers (UMich, Nature 2017)	MO_1307	Cutaneous squamous cell carcinoma	E214K	Missense_Mutation	SNP		2	0.19	3053
	Metastatic Solid Cancers (UMich, Nature 2017)	MO_1318	Breast invasive ductal carcinoma	V234I	Missense_Mutation	SNP			0.23	82
	MSS mixed solid tumors (Broad/Dana-Farber, Nat Genet 2018)	BLADDER-15330_CCPM _0700691 -Tumor-SM-AVI13	Bladder urothelial carcinoma	R158G	Missense_Mutation	SNP			0.13	165
	MSS mixed solid tumors (Broad/Dana-Farber, Nat Genet 2018)	CR3665_T	Cutaneous melanoma	R161C	Missense_Mutation	SNP		2	0.23	155
	MSS mixed solid tumors (Broad/Dana-Farber, Nat Genet 2018)	MEL-IPI_Pat11 -Tumor-SM-4DK17	Melanoma of unknown primary	E152K	Missense_Mutation	SNP			0.38	1981
	MSS mixed solid tumors (Broad/Dana-Farber, Nat Genet 2018)	MEL-IPI_Pat110 -Tumor-SM-4CU6X	Cutaneous melanoma	G67E	Missense_Mutation	SNP		2	0.1	6485
	MSS mixed solid tumors (Broad/Dana-Farber, Nat Genet 2018)	MEL-IPI_Pat139 -Tumor-SM-5VWJH	Cutaneous melanoma	S77F	Missense_Mutation	SNP			0.41	1387
	MSS mixed solid tumors (Broad/Dana-Farber, Nat Genet 2018)	MEL-IPI_Pat71 -Tumor-SM-4DK2W	Melanoma of unknown primary	P38S	Missense_Mutation	SNP			0.28	821
	MSS mixed solid tumors (Broad/Dana-Farber, Nat Genet 2018)	SD6494_T	Melanoma	G51E	Missense_Mutation	SNP		2	0.41	620
	Pan-cancer analysis of whole genomes (ICGC/TCGA, Nature 2020)	SP124298	Melanoma	G84E	Missense_Mutation	SNP	Diploid		0.22	2106
	Pan-cancer analysis of whole genomes (ICGC/TCGA, Nature 2020)	SP82644	Melanoma	G164R	Missense_Mutation	SNP	Diploid	1	0.28	1007
	Pan-cancer analysis of whole genomes (ICGC/TCGA, Nature 2020)	SP16886	Colorectal adenocarcinoma	R64Q	Missense_Mutation	SNP	Gain	5	0.16	3672
	Pan-cancer analysis of whole genomes (ICGC/TCGA, Nature 2020)	SP80615	Colorectal adenocarcinoma	R64Q	Missense_Mutation	SNP	Diploid	5	0.3	12012
	Pan-cancer analysis of whole genomes (ICGC/TCGA, Nature 2020)	SP83146	Melanoma	R64Q	Missense_Mutation	SNP	Diploid	5	0.39	1825
	Pan-cancer analysis of whole genomes (ICGC/TCGA, Nature 2020)	SP59464	Follicular lymphoma	R54Q	Missense_Mutation	SNP	Diploid		0.2	24
	Pan-cancer analysis of whole genomes (ICGC/TCGA, Nature 2020)	SP55235	Lung adenocarcinoma	P134Q	Missense_Mutation	SNP	Gain	2	0.18	697
	Pan-cancer analysis of whole genomes (ICGC/TCGA, Nature 2020)	SP57669	Lung squamous cell carcinoma	R86L	Missense_Mutation	SNP	Diploid	2	0.28	310
	Pan-cancer analysis of whole genomes (ICGC/TCGA, Nature 2020)	SP83146	Melanoma	E214K	Missense_Mutation	SNP	Diploid	2	0.5	1825
	Pan-cancer analysis of whole genomes (ICGC/TCGA, Nature 2020)	SP101716	Serous ovarian cancer	T209S	Missense_Mutation	SNP	Diploid	1	0.06	104
	Pan-cancer analysis of whole genomes (ICGC/TCGA, Nature 2020)	SP50171	Hepatocellular carcinoma	R158S	Missense_Mutation	SNP	Diploid		0.11	87
	Pan-cancer analysis of whole genomes (ICGC/TCGA, Nature 2020)	SP125763	Pancreatic adenocarcinoma	R174W	Missense_Mutation	SNP	Diploid		0.29	71
	Metastatic solid cancers (UMich, Nature 2017)	MO_1202	Prostate adenocarcinoma	GRHL2-RSPO2 fusion	fusion	FUSION			0.13	55
*Rspo3*	Metastatic solid cancers (UMich, Nature 2017)	MO_1307	Cutaneous squamous cell carcinoma	K162N	Missense_Mutation	SNP			0.2	3053
	Metastatic solid cancers (UMich, Nature 2017)	MO_1506	Esophageal adenocarcinoma	S241R	Missense_Mutation	SNP			0.41	302
	Metastatic solid cancers (UMich, Nature 2017)	MO_1524	Breast invasive lobular carcinoma	N109S	Missense_Mutation	SNP			0.06	174
	MSS mixed solid tumors (Broad/Dana-Farber, Nat Genet 2018)	NR8815_T	Cutaneous melanoma	R211Q	Missense_Mutation	SNP			0.71	1691
	MSS mixed solid tumors (Broad/Dana-Farber, Nat Genet 2018)	MEL-IPI_Pat110 -Tumor-SM-4CU6X	Cutaneous melanoma	T104I	Missense_Mutation	SNP			0.25	6485
	MSS mixed solid tumors (Broad/Dana-Farber, Nat Genet 2018)	MEL-IPI_Pat139 -Tumor-SM-5VWJH	Cutaneous melanoma	G123E	Missense_Mutation	SNP			0.4	1387
	MSS mixed solid tumors (Broad/Dana-Farber, Nat Genet 2018)	SD1494_T	Cutaneous melanoma	K71*	Nonsense_Mutation	SNP			0.49	918
	Pan-cancer analysis of whole genomes (ICGC/TCGA, Nature 2020)	SP82644	Melanoma	E217K	Missense_Mutation	SNP	Gain		0.31	1007
	Pan-cancer analysis of whole genomes (ICGC/TCGA, Nature 2020)	SP19983	Colorectal adenocarcinoma	T193A	Missense_Mutation	SNP	Diploid	1	0.41	92
	Pan-cancer analysis of whole genomes (ICGC/TCGA, Nature 2020)	SP19295	Mucinous Adenocarcinoma of the colon and rectum	A231V	Missense_Mutation	SNP	Diploid	1	0.19	1330
	Pan-cancer analysis of whole genomes (ICGC/TCGA, Nature 2020)	SP17905	Mucinous adenocarcinoma of the colon and rectum	F62S	Missense_Mutation	SNP	Diploid	1	0.11	11034
	Pan-cancer analysis of whole genomes (ICGC/TCGA, Nature 2020)	SP29559	Glioblastoma	C105S	Missense_Mutation	SNP	Diploid	2	0.41	44
	Pan-cancer analysis of whole genomes (ICGC/TCGA, Nature 2020)	SP124382	Melanoma	G212E	Missense_Mutation	SNP	Diploid	1	0.09	2785
	Pan-cancer analysis of whole genomes (ICGC/TCGA, Nature 2020)	SP116965	Pancreatic adenocarcinoma	D101Y	Missense_Mutation	SNP	ShallowDel		0.3	248
	Pan-cancer analysis of whole genomes (ICGC/TCGA, Nature 2020)	SP19295	Mucinous adenocarcinoma of the colon and rectum	R31Q	Missense_Mutation	SNP	Diploid		0.29	1330
	Pan-cancer analysis of whole genomes (ICGC/TCGA, Nature 2020)	SP55235	Lung adenocarcinoma	K208M	Missense_Mutation	SNP	ShallowDel		0.15	697
	Pan-cancer analysis of whole genomes (ICGC/TCGA, Nature 2020)	SP110822	Pancreatic adenocarcinoma	G215E	Missense_Mutation	SNP	Diploid		0.08	91
	MSK MetTropism (MSK, Cell 2021)	P-0032966-T01-IM6	Colon adenocarcinoma	RSPO3-PTPRK fusion - Archer	fusion	FUSION				1
	MSK MetTropism (MSK, Cell 2021)	P-0034040-T01-IM6	Rectal adenocarcinoma	RSPO3-PTPRK fusion - Archer	fusion	FUSION				6
	MSK MetTropism (MSK, Cell 2021)	P-0034063-T01-IM6	Rectal adenocarcinoma	RSPO3-PTPRK fusion - Archer	fusion	FUSION				8
	MSK MetTropism (MSK, Cell 2021)	P-0034853-T01-IM6	Colon adenocarcinoma	RSPO3-PTPRK fusion - Archer	fusion	FUSION				11
	MSK MetTropism (MSK, Cell 2021)	P-0035372-T01-IM6	Rectal Adenocarcinoma	RSPO3-PTPRK fusion - Archer	fusion	FUSION				9
	MSK MetTropism (MSK, Cell 2021)	P-0036261-T01-IM6	Colorectal adenocarcinoma	RSPO3-PTPRK fusion - Archer	fusion	FUSION				7
	MSK MetTropism (MSK, Cell 2021)	P-0036522-T01-IM6	Colorectal adenocarcinoma	RSPO3-PTPRK fusion - Archer	fusion	FUSION				7
	MSK MetTropism (MSK, Cell 2021)	P-0037180-T01-IM6	Rectal adenocarcinoma	RSPO3-PTPRK fusion - Archer	fusion	FUSION				7
	MSK MetTropism (MSK, Cell 2021)	P-0037571-T01-IM6	Colon adenocarcinoma	RSPO3-PTPRK fusion - Archer	fusion	FUSION				2
	MSK MetTropism (MSK, Cell 2021)	P-0037865-T01-IM6	Colon adenocarcinoma	RSPO3-PTPRK fusion - Archer	fusion	FUSION				9
	MSK MetTropism (MSK, Cell 2021)	P-0037968-T01-IM6	Colon adenocarcinoma	RSPO3-PTPRK fusion - Archer	fusion	FUSION				10
	MSK MetTropism (MSK, Cell 2021)	P-0038162-T01-IM6	Colon adenocarcinoma	RSPO3-PTPRK fusion - Archer	fusion	FUSION				3
	MSK MetTropism (MSK, Cell 2021)	P-0038239-T01-IM6	Rectal adenocarcinoma	RSPO3-PTPRK fusion - Archer	fusion	FUSION				6
	MSK MetTropism (MSK, Cell 2021)	P-0044388-T01-IM6	Rectal adenocarcinoma	RSPO3-PTPRK fusion - Archer	fusion	FUSION				6
	MSK MetTropism (MSK, Cell 2021)	P-0045139-T01-IM6	Colon adenocarcinoma	RSPO3-PTPRK fusion - Archer	fusion	FUSION				3
	MSK MetTropism (MSK, Cell 2021)	P-0045531-T01-IM6	Colon adenocarcinoma	RSPO3-PTPRK fusion - Archer	fusion	FUSION				4
	MSK MetTropism (MSK, Cell 2021)	P-0046018-T01-IM6	Colon adenocarcinoma	RSPO3-PTPRK fusion - Archer	fusion	FUSION				5
	MSK MetTropism (MSK, Cell 2021)	P-0046861-T01-IM6	Colon adenocarcinoma	RSPO3-PTPRK fusion - Archer	fusion	FUSION				10
	MSK MetTropism (MSK, Cell 2021)	P-0048933-T01-IM6	Colorectal adenocarcinoma	RSPO3-PTPRK fusion - Archer	fusion	FUSION				10
	MSK MetTropism (MSK, Cell 2021)	P-0050356-T01-IM6	Rectal adenocarcinoma	RSPO3-PTPRK fusion - Archer	fusion	FUSION				6
*Rspo4*	Metastatic solid cancers (UMich, Nature 2017)	MO_1146	Cutaneous melanoma	A173V	Missense_Mutation	SNP			0.45	2219
	Metastatic solid cancers (UMich, Nature 2017)	TP_2024	Small cell lung cancer	H175D	Missense_Mutation	SNP			0.86	108
	MSS Mixed Solid Tumors (Broad/Dana-Farber, Nat Genet 2018)	LSD3484_T	Cutaneous melanoma	R81C	Missense_Mutation	SNP			0.33	529
	MSS mixed solid tumors (Broad/Dana-Farber, Nat Genet 2018)	MEL-IPI_Pat103 -Tumor-SM-4CU6Q	Melanoma of unknown primary	S159L	Missense_Mutation	SNP		1	0.07	760
	MSS mixed solid tumors (Broad/Dana-Farber, Nat Genet 2018)	HNSCC-306 -Tumor-SM-CK9Y6	Oropharyn × squamous cell carcinoma	G28S	Missense_Mutation	SNP			0.12	65
	MSS mixed solid tumors (Broad/Dana-Farber, Nat Genet 2018)	SD7357_T	Melanoma	P120L	Missense_Mutation	SNP			0.2	1211
	Pan-cancer analysis of whole genomes (ICGC/TCGA, Nature 2020)	SP17905	Mucinous adenocarcinoma of the colon and rectum	S159L	Missense_Mutation	SNP	Diploid	1	0.2	11034
	Pan-cancer analysis of whole genomes (ICGC/TCGA, Nature 2020)	SP101616	Serous ovarian cancer	Q233K	Missense_Mutation	SNP	Diploid	1	0.24	105
	Pan-cancer analysis of whole genomes (ICGC/TCGA, Nature 2020)	SP60842	Serous ovarian cancer	V15L	Missense_Mutation	SNP	Diploid		0.42	85

^a^Variant allele frequency in the tumor sample; ^b^Total number of nonsynonymous mutations in the sample.

Mutations in one gene may affect both its expression and the expression of other genes. To identify the altered genes, we used the muTarget database (https://www.mutarget.com/) to analyze samples harboring mutated RSPOs. As displayed in Table S1, some tumors, such as bladder cancer, did not yield consistent results due to insufficient mutated patient samples. RSPO mutations affected the expression of many genes in colon adenocarcinoma, lung adenocarcinoma, and other tumors. These data are useful for studying new drug targets in a cohort of patients with a given mutation.

## Immune correlates of RSPOs in cancer

Immuno-oncology is revolutionizing cancer treatment as a new pillar of modern cancer treatment. Immune checkpoint blockade has been widely used in the clinical treatment of tumors, but the lack of tumor antigens and the failure to effectively initiate adaptive immunity are important reasons for the poor efficacy of immunotherapy. We investigated the immune correlations of RSPOs in multiple tumors using bioinformatics.

RNA-sequencing expression profiles and corresponding clinical information were downloaded from the TCGA dataset. The immune cell infiltration was analyzed using the ‘immunedeconv’ R software package. The heatmap of TIMER immune score and RSPOs expression in multiple tumor tissues is presented in Figure S1. RSPO3 was correlated with the score for immune cell infiltration, suggesting its potential immunotherapeutic value.

SIGLEC15, IDO1, CD274, HAVCR2, PDCD1, CTLA4, LAG3 and PDCD1LG2 are the transcripts associated with the immune checkpoint. The expression value of the immune checkpoint-related genes was observed and evaluated in Figure S2. RSPO1 and RSPO3 expressions were significantly associated with immune checkpoint-related gene expression in a variety of tumors. This raises the potential to develop immune checkpoint-blocking therapy.

## Molecular mechanism of RSPOs involved in tumor progression

A considerable amount of research demonstrated the critical role of RSPOs in cancer. The loss of regulation of the Wnt/β-catenin signaling pathway is linked to the onset and progression of a variety of tumors. Therefore, inhibitors, antagonists, and agonists are created to treat solid tumors and hematological malignancies [53]. Porcupine inhibitors [54], Wnt/FZD antagonists [55], LRP5/6 inhibitors [56], and some targeted medicaments for the β‑catenin‑destruction complex have been developed [57,58]. Seeber et al. reported that *RNF43* mutation and *RSPO* fusion in colon cancer were caused by dysregulated Wnt/β-catenin signaling, suggesting that changes in these genes might present new therapeutic targets [23]. In addition to the above, natural agents play a crucial role in therapy, such as gigantol, a bibenzyl compound from orchid species, which could suppress Wnt/β-catenin signaling via downregulating the expressions of phosphorylated LRP6 and cytosolic β-catenin in breast cancer cells [59]. Nimbolide, a limonoid present in neem leaves, could eliminate the canonical Wnt/β-catenin signaling and simultaneously induce the endogenous apoptosis of hepatocarcinoma cells [60].

The aforementioned studies actuated further research to better understand the roles of RSPOs in tumor progression. Considering the irreplaceable importance of the Wnt signaling pathway in determining cell fate decisions and tissue formation, proliferation and homeostasis, RSPOs may act as essential participants in these processes and prospective targeted therapies [61]. RSPOs are largely involved in tumor regulation through the Wnt pathway.

RSPOs regulate Wnt receptor turnover, which has been regarded as an essential regulatory mechanism of Wnt signaling. Cancer cells tend to inactivate this negative feedback mechanism by way of the ZNRF3/RNF43 mutation or RSPO overexpression, which causes continuous Wnt/β-catenin signaling [62]. Additionally, RSPOs have been reported to significantly increase the sensitivity of target cells to Wnt ligands [63].

RSPO1 is an upstream regulator in the Wnt/β-catenin pathway, which can help ovarian cancer cells grow, survive, and migrate. Liu et al. illustrated that the transcription and protein expression of RSPO1 in ovarian cancer cell lines and tissues were upregulated compared with normal samples. The downregulation of RSPO1 prevented the proliferation and migration of ovarian cancer cells, and induced apoptosis [64]. Meanwhile, RSPO1 also played a different role in adenoma. Lähde et al. added RSPO1 to mice models and found that the number of adenoma organoids in mice decreased significantly and interfered with the expression of Wnt target genes [65].

RSPO2 contributes to the secretory activation of the canonical Wnt signaling pathway. Gong et al. investigated the influence of RSPO2 on human dental pulp stem/progenitor cells (hDPSCs) and found that RSPO2 mediated the canonical Wnt/β-catenin signaling pathway, leading to the proliferation of hDPSCs [66]. Conboy et al. constructed an *in vivo* model and discovered that the overexpression of RSPO2 in the liver reduced Wnt/β-catenin signaling and increased liver tumor development when paired with the deletion of transformation-related protein 53 (Trp53) [67]. Zhang et al. observed that the overexpression of RSPO2 led to many phenotypes of squamous cell carcinoma (SCC), such as growth, migration, epithelial-mesenchymal transition (EMT), and stem-like properties, while the knockout of RSPO2 rescued these phenotypes. In tongue squamous cell carcinoma (TSCC), RSPO2 silencing reduced the expression of LGR4, resulting in LRP6 phosphorylation and β-catenin nuclear translocation [68]. However, RSPO2 exhibited two contradictory roles in colon cancer. The research of Al-Samadi et al. indicated that RSPO2 enhanced cancer progression by enriching LGR5^+^ stem cells, and also suppressed cancer progression by restraining the Wnt/β-catenin pathway, thereby reducing cancer cell proliferation and metastasis [69].

RSPO3, a vital activator of the Wnt/β-catenin signaling pathway, is involved in the development of a variety of malignancies. Chen et al. overexpressed RSPO3 in JEG-3 cells and detected the expression of biomarkers related to tumorigenicity. The overexpression of RSPO3 accelerated the proliferation of the JEG-3 cells and suppressed their migration, invasion, and mortality [70]. Fischer et al. discovered that in colorectal cancer (CRC), the activation mutation of the Wnt/β-catenin pathway induced tumor sensitivity to the synergistic effect of Wnt-RSPO [71]. Fujiwara et al. reported that C-mannosylation of RSPO3 enhanced the activation of Wnt/β-catenin signaling [72]. Picco et al. found that Wnt pathway activation through *RSPO3* gene rearrangements could be considered a potential target in CRC [73]. Zhang et al. demonstrated that mice fibrosis model and patients with IPF and nonalcoholic steatohepatitis (NASH) had high levels of RSPO3, suggesting that RSPO3 might induce the secretion of fibrogenic chemokines and cytokines in the lung and hepatocytes [74]. Nevertheless, Rong et al. reported that zebrafish RSPO3 downregulated the intensity of Wnt/β-catenin signaling. In contrast, the constrained expression of RSPO3 eliminated the effect of exogenous Wnt3a and decreased the endogenous Wnt signaling activity [75]. Tang et al. found that the expression of the Wnt signaling booster gene *RSPO3* was linked to a better prognosis. Exogenous expression of RSPO3 in tumors can increase the cytotoxic effector cells infiltration and function, resulting in tumor regression [9].

Unlike RSPO1-3, RSPO4 has not been identified in almost all kinds of tumor tissues and normal tissues yet. Wu et al. demonstrated that the expression level of RSPO4 was not directly related to the overall survival (OS) of patients with lung cancer, indicating the functional differences between RSPO4 and RSPO1-3 [48].

### RSPOs regulate tumor progression through other signaling pathways

Although RSPOs are most recognized for their ability to agonize and synergize the canonical Wnt/β-catenin pathway, recent research has also found that RSPOs can be involved in tumor progression through other signaling pathways.

*RSPO2*, a new cancer inhibitor gene, was able to prevent HCC from proliferating and invading the MAPK signaling pathway. Zheng et al. observed that the MAPK signaling pathway was changed after transfecting the shRNAs against *RSPO2*. Furthermore, *RSPO2* knockout enhanced the tumorigenicity of HCC cells [6].

RSPO3 was reported to remodel the endothelial vasculature by activating a non-canonical Wnt signaling pathway. Scholz et al. discovered that inducible endothelial *Rspo3* deletion (Rspo3-iECKO) mice had vascular defects, and this phenotype was consistent with that induced by non-canonical Wnt signal. This was due to the deletion of Wnt secretion factor *Evi*/*Wls*, inferring that RSPO3 and EVI/WLS might have a co-coordinated role [76]. Furthermore, RSPO3 can also act as a suppressor of ERK/FGF signaling. Zhang et al. demonstrated that *RSPO3* knockout enhanced the osteogenic capacity of human adipose-derived stem cells (hASCs), which were rescued *via* suppression of ERK signaling. Besides, the lack of LGR4 abolished RSPO3-induced ERK1/2 signaling suppression [77]. On the other hand, Gu et al. confirmed that RSPO3 could enhance ovarian cancer invasiveness by activating the PI3K/AKT pathway, but not the Wnt/β-catenin pathway [78]. LGR5/4 are positive regulators of NF-κB signaling. The study of Lai et al. suggested that the proliferation of LGR5+ adult stem cells and intestinal crypts might be regulated by both NF-κB and Wnt signaling [79].

## Concluding remarks

The RSPO family is known to activate the canonical Wnt/β-catenin signaling pathway by playing the role of ligand for the LGR4-6 receptors. An increasing amount of studies has reflected that abnormal RSPOs, mainly characterized by gene fusion or abnormal expression, have been recorded in patients with cancer of any kind. At present, most of our knowledge about the molecular activity of RSPO has been gained through intestinal studies. While these provide valuable and pertinent insights, only a small fraction of the mechanism has been uncovered. This would include the unknown role of RSPOs in non-Wnt pathways. Since RSPOs are secreted proteins, this greatly increases the diversity of their molecular mechanisms in tumor progression, despite the lack of available data.

In the future, it is of vital importance to comprehend the molecular mechanisms of RSPOs. Tissue specificity should also be considered (since the roles of RSPOs may differ in different tissues) to provide a good direction for clinical studies. The wide range of indications for the carcinogenic effect of RSPOs has led to the exploration of probable treatment methods, and RSPOs have been identified as prospective targets for tumor treatment, especially in tumor immunotherapy. Recent studies have also implied that inhibitors of RSPOs or their receptors (such as LGR5) are being evaluated as candidate targets for cancer therapeutic intervention, which provides a valuable framework for developing new therapeutic strategies.

## Supplementary Material

Supplemental Material

Supplemental Material

Supplemental Material
