# Supplementary material for: R-spondin family biology and emerging linkages to cancer
Source: Ann Med. 2023 Jan 16;55(1):428–46. doi: 10.1080/07853890.2023.2166981 (PMC9848353; doi:10.1080/07853890.2023.2166981)
Supplement: Supplemental Material [file IANN_A_2166981_SM6696.docx]

Table S1. Altered expression in samples harbouring a mutated RSPOs

| Tumor type | Expression of | Mean mutant (n=26) | Mean wild (n=370) | FC (mutant/wild) | Direction | p-value |
| --- | --- | --- | --- | --- | --- | --- |
| Colon Adenocarcinoma | KCTD1 | 264.19 | 139.35 | 1.9 | up | 1.75e-05 |
|  | GNLY | 379.73 | 187.43 | 2.03 | up | 1.85e-05 |
|  | PLK2 | 1412.88 | 752.42 | 1.88 | up | 1.91e-05 |
|  | ZIC2 | 364.35 | 183.2 | 1.99 | up | 1.96e-05 |
|  | USP18 | 309.96 | 165.55 | 1.87 | up | 2.54e-05 |
|  | IDO1 | 2286.27 | 559.8 | 4.08 | up | 2.69e-05 |
|  | TUSC8 | 41.81 | 116.51 | 2.78 | down | 2.99e-05 |
|  | GAS2L1 | 867.08 | 527.36 | 1.64 | up | 4.36e-05 |
|  | TYMP | 3413.15 | 1654.42 | 2.06 | up | 6.08e-05 |
|  | TNFSF9 | 486.54 | 276.65 | 1.76 | up | 7.72e-05 |
|  | TRIB2 | 1632.38 | 628.92 | 2.6 | up | 8.34e-05 |
|  | CHD3 | 3714.96 | 2486.63 | 1.49 | up | 8.47e-05 |
|  | MYRIP | 144.62 | 326.9 | 2.27 | down | 8.75e-05 |
|  | KIAA1549L | 290.12 | 130.2 | 2.23 | up | 9.38e-05 |
|  | PAH | 14.88 | 126.21 | 8.33 | down | 1.07e-04 |
|  | XAF1 | 445.12 | 236.95 | 1.88 | up | 1.08e-04 |
|  | PKP1 | 215.38 | 396.62 | 1.85 | down | 1.21e-04 |
|  | LINC01006 | 201.92 | 326.28 | 1.61 | down | 1.30e-04 |
|  | EIF4EBP3 | 88.27 | 148.75 | 1.69 | down | 2.01e-04 |
|  | RNF152 | 238.23 | 165.9 | 1.44 | up | 2.04e-04 |
|  | APOL2 | 1685.08 | 1018.35 | 1.65 | up | 2.11e-04 |
|  | ODF3B | 435.88 | 246.84 | 1.77 | up | 2.15e-04 |
|  | WARS | 13193.5 | 5249.14 | 2.51 | up | 2.22e-04 |
|  | COL9A1 | 76.23 | 140.23 | 1.85 | down | 2.24e-04 |
|  | COL2A1 | 10.15 | 120.69 | 12.5 | down | 2.38e-04 |
|  | MEP1A | 1224.73 | 2901.67 | 2.38 | down | 2.56e-04 |
|  | LOC101928881 | 84.85 | 152.47 | 1.79 | down | 2.66e-04 |
|  | SYT7 | 1077.27 | 2001.16 | 1.85 | down | 2.71e-04 |
|  | VANGL2 | 263.38 | 567.44 | 2.17 | down | 2.93e-04 |
|  | DHRS12 | 241.12 | 349.82 | 1.45 | down | 2.98e-04 |
|  | TNFAIP2 | 1940.23 | 1238.91 | 1.57 | up | 3.05e-04 |
|  | KDM7A-DT | 451.69 | 650.29 | 1.45 | down | 3.53e-04 |
|  | TCF7 | 1032.73 | 1582.56 | 1.54 | down | 3.59e-04 |
|  | APOL6 | 3328.58 | 2309.41 | 1.44 | up | 3.62e-04 |
|  | LAG3 | 318.92 | 112.09 | 2.85 | up | 3.91e-04 |
|  | RNF32 | 87.69 | 139.33 | 1.59 | down | 3.93e-04 |
|  | MIAT | 287.38 | 169.21 | 1.7 | up | 3.98e-04 |
|  | EEF1AKMT1 | 170.19 | 248.86 | 1.47 | down | 4.25e-04 |
|  | GBP4 | 2797.65 | 1104.18 | 2.53 | up | 4.49e-04 |
|  | APOL1 | 5589.62 | 3371.26 | 1.66 | up | 4.59e-04 |
|  | FECH | 612.12 | 393.79 | 1.55 | up | 4.76e-04 |
|  | PKM | 52127.96 | 34586.6 | 1.51 | up | 4.81e-04 |
|  | SNX24 | 223.92 | 147.99 | 1.51 | up | 4.95e-04 |
|  | OAS2 | 2484.12 | 1192.68 | 2.08 | up | 4.99e-04 |
|  | IRF7 | 1046.46 | 726.21 | 1.44 | up | 5.26e-04 |
|  | LAP3 | 4411.54 | 3023.49 | 1.46 | up | 5.26e-04 |
|  | TDGF1 | 395.92 | 835.32 | 2.13 | down | 5.28e-04 |
|  | NXPH4 | 741.35 | 206.02 | 3.6 | up | 5.46e-04 |
|  | ACSL6 | 318.62 | 719.16 | 2.27 | down | 5.60e-04 |
|  | FRMD1 | 130.77 | 285.41 | 2.17 | down | 5.65e-04 |
|  | H3F3A | 549.31 | 873.56 | 1.59 | down | 5.79e-04 |
|  | SLC6A4 | 63.88 | 120.77 | 1.89 | down | 5.94e-04 |
|  | TNFRSF11A | 1584.23 | 1056.15 | 1.5 | up | 6.02e-04 |
|  | CD274 | 222.88 | 120.68 | 1.85 | up | 6.81e-04 |
|  | ENO2 | 1260.69 | 775.16 | 1.63 | up | 6.85e-04 |
|  | SLC43A3 | 892.31 | 440.31 | 2.03 | up | 7.60e-04 |
|  | MAPK11 | 213.69 | 122.91 | 1.74 | up | 7.94e-04 |
|  | NKD1 | 1382.12 | 2810.48 | 2.04 | down | 8.08e-04 |
|  | PTCH1 | 828.69 | 1374.26 | 1.67 | down | 8.44e-04 |
|  | IFI44L | 872.88 | 341.08 | 2.56 | up | 8.72e-04 |
|  | CHN2 | 409.62 | 842.13 | 2.04 | down | 8.83e-04 |
|  | CTTNBP2 | 161.5 | 354.32 | 2.17 | down | 8.91e-04 |
|  | GZMA | 314.54 | 158.23 | 1.99 | up | 9.32e-04 |
|  | SLCO1B3 | 227.96 | 120.53 | 1.89 | up | 9.43e-04 |
|  | SLC2A9 | 102.62 | 152.33 | 1.49 | down | 9.68e-04 |
|  | OTULINL | 1259.96 | 1882.35 | 1.49 | down | 9.71e-04 |
|  | LRRC36 | 50.65 | 106.36 | 2.08 | down | 1.05e-03 |
|  | BST2 | 4992.42 | 2182.3 | 2.29 | up | 1.05e-03 |
|  | IFI6 | 5407.81 | 3136.23 | 1.72 | up | 1.06e-03 |
|  | APOL3 | 991.27 | 534.21 | 1.86 | up | 1.08e-03 |
|  | EPM2AIP1 | 343.46 | 533.37 | 1.56 | down | 1.09e-03 |
|  | ASPHD2 | 436.27 | 249.54 | 1.75 | up | 1.13e-03 |
|  | A1CF | 382.42 | 664.82 | 1.72 | down | 1.14e-03 |
|  | LOC283177 | 128.31 | 251.64 | 1.96 | down | 1.18e-03 |
|  | SLC13A3 | 98.92 | 251.11 | 2.56 | down | 1.19e-03 |
|  | CIITA | 1407.15 | 597.55 | 2.35 | up | 1.19e-03 |
|  | IFIT3 | 1516.81 | 704.57 | 2.15 | up | 1.24e-03 |
|  | SERPINB5 | 3154.38 | 1982.54 | 1.59 | up | 1.24e-03 |
|  | FRMD5 | 332.85 | 218.53 | 1.52 | up | 1.26e-03 |
|  | IHH | 1446.23 | 2362.38 | 1.64 | down | 1.28e-03 |
|  | CRYL1 | 1738.38 | 2623.11 | 1.52 | down | 1.30e-03 |
|  | PIAS2 | 719.42 | 433.86 | 1.66 | up | 1.35e-03 |
|  | AXIN2 | 2408.35 | 4455.87 | 1.85 | down | 1.36e-03 |
|  | ZSWIM3 | 171.19 | 259.75 | 1.52 | down | 1.36e-03 |
|  | SLC6A14 | 1083.38 | 476.26 | 2.27 | up | 1.38e-03 |
|  | CELP | 70.27 | 162.19 | 2.33 | down | 1.39e-03 |
|  | SAMD9L | 902.62 | 518.7 | 1.74 | up | 1.41e-03 |
|  | SLC22A3 | 408.65 | 651.14 | 1.59 | down | 1.42e-03 |
|  | IL2RB | 603.35 | 349.93 | 1.72 | up | 1.51e-03 |
|  | CD2 | 359.23 | 245.92 | 1.46 | up | 1.53e-03 |
|  | STAT1 | 10358.73 | 6071.66 | 1.71 | up | 1.55e-03 |
|  | HSD11B2 | 2178.73 | 3371.15 | 1.54 | down | 1.57e-03 |
|  | HOXD8 | 242.69 | 144.19 | 1.68 | up | 1.61e-03 |
|  | RARRES3 | 2101.62 | 934.28 | 2.25 | up | 1.62e-03 |
|  | PARD6G | 148.38 | 100.88 | 1.47 | up | 1.64e-03 |
|  | PARP9 | 2550.08 | 1765.79 | 1.44 | up | 1.66e-03 |
|  | DDAH2 | 1615.27 | 2395.52 | 1.49 | down | 1.66e-03 |
|  | NPFFR1 | 57.46 | 114.64 | 2 | down | 1.67e-03 |
|  | HIVEP3 | 287.04 | 198.72 | 1.44 | up | 1.67e-03 |
|  | ISG15 | 2487.54 | 1473.98 | 1.69 | up | 1.69e-03 |
|  | NT5DC3 | 1086.27 | 699.27 | 1.55 | up | 1.73e-03 |
|  | LY6G6F-LY6G6D | 90.31 | 282.22 | 3.12 | down | 1.73e-03 |
|  | USP13 | 519.19 | 349.03 | 1.49 | up | 1.74e-03 |
|  | ZNF717 | 119.46 | 176.74 | 1.47 | down | 1.75e-03 |
|  | UBASH3B | 293.88 | 192.73 | 1.52 | up | 1.75e-03 |
|  | IRF1 | 4765.23 | 3182.41 | 1.5 | up | 1.75e-03 |
|  | AHNAK2 | 1509.27 | 916.99 | 1.65 | up | 1.78e-03 |
|  | SLC1A1 | 868.19 | 463.72 | 1.87 | up | 1.82e-03 |
|  | OAS3 | 4879.12 | 2970.88 | 1.64 | up | 1.82e-03 |
|  | SKA1 | 459.27 | 307.85 | 1.49 | up | 1.87e-03 |
|  | SLC9A7 | 625.77 | 363.19 | 1.72 | up | 1.94e-03 |
|  | LYSMD2 | 412.5 | 281.67 | 1.46 | up | 1.97e-03 |
|  | GPR160 | 1862.5 | 2725.18 | 1.47 | down | 1.97e-03 |
|  | TPBG | 802.27 | 517.97 | 1.55 | up | 2.01e-03 |
|  | PLAGL2 | 1897.12 | 2808.58 | 1.47 | down | 2.10e-03 |
|  | CARD16 | 282.73 | 196.28 | 1.44 | up | 2.16e-03 |
|  | FRAS1 | 421.58 | 235.97 | 1.79 | up | 2.20e-03 |
|  | CCL24 | 478.73 | 1055.25 | 2.22 | down | 2.20e-03 |
|  | RNF144A | 663.62 | 435.32 | 1.52 | up | 2.21e-03 |
|  | DUSP4 | 2709.73 | 1506.47 | 1.8 | up | 2.23e-03 |
|  | CCR5 | 242.81 | 144.91 | 1.68 | up | 2.24e-03 |
|  | ZNF606 | 87.46 | 157.37 | 1.79 | down | 2.28e-03 |
|  | HSPA4L | 700.88 | 375.83 | 1.86 | up | 2.29e-03 |
|  | LOC102723878 | 85.08 | 247.5 | 2.94 | down | 2.31e-03 |
|  | DLEU1 | 202.81 | 292.43 | 1.45 | down | 2.32e-03 |
|  | SLC25A37 | 1554.81 | 1054.81 | 1.47 | up | 2.37e-03 |
|  | RPS21 | 16217.19 | 24706.26 | 1.52 | down | 2.42e-03 |
|  | CCL4 | 327.58 | 192.79 | 1.7 | up | 2.46e-03 |
|  | LY6G6D | 88 | 291.66 | 3.33 | down | 2.47e-03 |
|  | MAPK12 | 289.85 | 159.66 | 1.82 | up | 2.47e-03 |
|  | EXOC3L4 | 288.04 | 149 | 1.93 | up | 2.54e-03 |
|  | PRDX5 | 8164.12 | 12587.59 | 1.54 | down | 2.58e-03 |
|  | GBP5 | 626.12 | 392 | 1.6 | up | 2.61e-03 |
|  | OSER1-DT | 155.46 | 260.81 | 1.67 | down | 2.71e-03 |
|  | SNPH | 277.85 | 149.98 | 1.85 | up | 2.74e-03 |
|  | ZNF470 | 97.38 | 161.01 | 1.67 | down | 2.74e-03 |
|  | CYP39A1 | 212.81 | 308.62 | 1.45 | down | 2.82e-03 |
|  | TNNC2 | 180.54 | 428.89 | 2.38 | down | 2.84e-03 |
|  | KRT23 | 745.5 | 1953.38 | 2.63 | down | 2.85e-03 |
|  | UBE2L6 | 3646.96 | 2218.45 | 1.64 | up | 2.87e-03 |
|  | IFIT2 | 580.62 | 256.7 | 2.26 | up | 2.88e-03 |
|  | PRSS12 | 575.08 | 389.66 | 1.48 | up | 2.90e-03 |
|  | BIRC3 | 1987.92 | 1119.13 | 1.78 | up | 2.98e-03 |
|  | SLC44A5 | 59 | 164.64 | 2.78 | down | 2.99e-03 |
|  | PTAFR | 603.65 | 384.66 | 1.57 | up | 3.07e-03 |
|  | DPEP1 | 3180.92 | 7141.99 | 2.22 | down | 3.13e-03 |
|  | FRRS1 | 191.54 | 129.49 | 1.48 | up | 3.15e-03 |
|  | PRDM8 | 180.81 | 96.72 | 1.87 | up | 3.16e-03 |
|  | HAPLN3 | 750.54 | 469.74 | 1.6 | up | 3.20e-03 |
|  | ETV5 | 934.38 | 509.44 | 1.83 | up | 3.20e-03 |
|  | LRRC31 | 219 | 342.83 | 1.56 | down | 3.32e-03 |
|  | TNFSF13B | 276.77 | 147.68 | 1.87 | up | 3.36e-03 |
|  | LOC105372629 | 129.96 | 256.56 | 1.96 | down | 3.41e-03 |
|  | MUC1 | 8398 | 4793.78 | 1.75 | up | 3.62e-03 |
|  | XPNPEP2 | 169.73 | 439.34 | 2.56 | down | 3.66e-03 |
|  | CEACAM5 | 59385.69 | 90382.61 | 1.52 | down | 3.66e-03 |
|  | UBE2V1 | 214.62 | 313.25 | 1.45 | down | 3.67e-03 |
|  | GABRP | 1516.38 | 234.18 | 6.48 | up | 3.79e-03 |
|  | PFDN4 | 489.69 | 743.37 | 1.52 | down | 3.80e-03 |
|  | TIGIT | 158.04 | 104.41 | 1.51 | up | 3.82e-03 |
|  | TP53RK | 775.96 | 1130.85 | 1.45 | down | 3.89e-03 |
|  | ZNF503-AS1 | 59.96 | 123.24 | 2.04 | down | 3.95e-03 |
|  | RUBCNL | 1437.31 | 2665.26 | 1.85 | down | 4.04e-03 |
|  | IFI44 | 687.12 | 338.88 | 2.03 | up | 4.07e-03 |
|  | NAALADL2 | 284.08 | 457.66 | 1.61 | down | 4.08e-03 |
|  | PLA2G2A | 10203.5 | 6056.52 | 1.68 | up | 4.08e-03 |
|  | NKG7 | 259.42 | 147.84 | 1.75 | up | 4.11e-03 |
|  | CAB39L | 378.19 | 711.14 | 1.89 | down | 4.12e-03 |
|  | SPATA18 | 296.92 | 200.89 | 1.48 | up | 4.14e-03 |
|  | LPCAT1 | 2845.23 | 1810.96 | 1.57 | up | 4.14e-03 |
|  | RN7SK | 64.08 | 8534.43 | 100 | down | 4.20e-03 |
|  | MT2A | 2870.62 | 1629.59 | 1.76 | up | 4.21e-03 |
|  | PIK3CD | 392 | 269.9 | 1.45 | up | 4.26e-03 |
|  | TFAP2A | 429.19 | 195.64 | 2.19 | up | 4.27e-03 |
|  | PSORS1C1 | 80.15 | 129.85 | 1.61 | down | 4.35e-03 |
|  | SPIN3 | 67.77 | 106.36 | 1.56 | down | 4.37e-03 |
|  | NFATC1 | 311.92 | 192.32 | 1.62 | up | 4.37e-03 |
|  | CPLX2 | 50.54 | 143.37 | 2.86 | down | 4.39e-03 |
|  | CD96 | 172.58 | 113.31 | 1.52 | up | 4.43e-03 |
|  | CBR3 | 184 | 117.19 | 1.57 | up | 4.56e-03 |
|  | NOTUM | 1477.54 | 2185.22 | 1.47 | down | 4.73e-03 |
|  | APOBEC3C | 1421.5 | 892.6 | 1.59 | up | 4.85e-03 |
|  | PTPRU | 800.31 | 491.76 | 1.63 | up | 4.91e-03 |
|  | IL12RB1 | 253.46 | 127.36 | 1.99 | up | 4.92e-03 |
|  | CXCL14 | 2373.46 | 4883.12 | 2.04 | down | 4.93e-03 |
|  | CXCL9 | 2222.92 | 1008.89 | 2.2 | up | 4.97e-03 |
|  | IL18BP | 564.62 | 344.71 | 1.64 | up | 5.03e-03 |
|  | TRIM69 | 1238.27 | 856.08 | 1.45 | up | 5.04e-03 |
|  | DNAJC3-DT | 87.58 | 132.56 | 1.52 | down | 5.07e-03 |
|  | MOGAT3 | 367.27 | 632 | 1.72 | down | 5.09e-03 |
|  | MYOM3 | 411.62 | 683.14 | 1.67 | down | 5.10e-03 |
|  | VAV3 | 1601.35 | 2566.94 | 1.61 | down | 5.10e-03 |
|  | PMFBP1 | 60.92 | 113.25 | 1.85 | down | 5.15e-03 |
|  | PARP14 | 3673.96 | 2465.48 | 1.49 | up | 5.17e-03 |
|  | EFNA5 | 186.58 | 121.16 | 1.54 | up | 5.21e-03 |
|  | APOBEC3G | 473.35 | 204.6 | 2.31 | up | 5.21e-03 |
|  | NPSR1 | 426.38 | 149.48 | 2.85 | up | 5.29e-03 |
|  | GBP1 | 2108.35 | 1295.43 | 1.63 | up | 5.34e-03 |
|  | P2RY6 | 171.58 | 106.06 | 1.62 | up | 5.43e-03 |
|  | IFITM10 | 166.58 | 111.26 | 1.5 | up | 5.49e-03 |
|  | PRR15 | 2233.04 | 3223.06 | 1.45 | down | 5.57e-03 |
|  | ERAP2 | 2430.08 | 1359.98 | 1.79 | up | 5.63e-03 |
|  | ARL4C | 1522.88 | 1051.46 | 1.45 | up | 5.67e-03 |
|  | AIF1L | 351.08 | 194.49 | 1.81 | up | 5.70e-03 |
|  | GTF3A | 3904.85 | 5688.36 | 1.45 | down | 5.73e-03 |
|  | ZNF736 | 192.38 | 315.65 | 1.64 | down | 5.77e-03 |
|  | HPSE | 490.73 | 286.65 | 1.71 | up | 5.80e-03 |
|  | RSAD2 | 579.15 | 272.66 | 2.12 | up | 5.94e-03 |
|  | FOXC1 | 304.08 | 166.79 | 1.82 | up | 6.00e-03 |
|  | ZNF141 | 100.96 | 153.06 | 1.52 | down | 6.02e-03 |
|  | CXCL10 | 2199.62 | 700.98 | 3.14 | up | 6.02e-03 |
|  | NEK3 | 739.35 | 1095.83 | 1.49 | down | 6.27e-03 |
|  | CD8A | 333.23 | 204.29 | 1.63 | up | 6.29e-03 |
|  | CD7 | 348.96 | 207.95 | 1.68 | up | 6.32e-03 |
|  | FLVCR2 | 231 | 152.26 | 1.52 | up | 6.34e-03 |
|  | KCNK1 | 1376.38 | 913.68 | 1.51 | up | 6.44e-03 |
|  | STS | 895.88 | 579.59 | 1.55 | up | 6.47e-03 |
|  | GGH | 2442.5 | 3991.12 | 1.64 | down | 6.53e-03 |
|  | MX1 | 4201.65 | 1928.75 | 2.18 | up | 6.69e-03 |
|  | PRSS21 | 361.31 | 111.59 | 3.24 | up | 6.86e-03 |
|  | PCK1 | 873 | 1322.35 | 1.52 | down | 7.00e-03 |
|  | LYPD5 | 194.12 | 125.48 | 1.55 | up | 7.30e-03 |
|  | AGT | 520.46 | 784.65 | 1.52 | down | 7.30e-03 |
|  | AREG | 1603.62 | 2811.04 | 1.75 | down | 7.44e-03 |
|  | MUC5AC | 967.12 | 1704.45 | 1.75 | down | 7.47e-03 |
|  | SREBF1 | 5588.46 | 3691.26 | 1.51 | up | 7.52e-03 |
|  | L1TD1 | 414.15 | 988.78 | 2.38 | down | 7.53e-03 |
|  | FCGR3A | 1441.88 | 876.45 | 1.65 | up | 7.56e-03 |
|  | RGS16 | 823.31 | 563.41 | 1.46 | up | 7.60e-03 |
|  | SLC15A3 | 1042.62 | 622.8 | 1.67 | up | 7.62e-03 |
|  | ITGAL | 525.38 | 347.51 | 1.51 | up | 7.76e-03 |
|  | ABAT | 505.19 | 822.72 | 1.64 | down | 8.09e-03 |
|  | IGF2BP3 | 202.69 | 122.03 | 1.66 | up | 8.17e-03 |
|  | TRNP1 | 453.27 | 267.5 | 1.69 | up | 8.28e-03 |
|  | BAHCC1 | 757.69 | 497.65 | 1.52 | up | 8.31e-03 |
|  | CMPK2 | 615.5 | 300.64 | 2.05 | up | 8.35e-03 |
|  | DBN1 | 2273.88 | 1474.14 | 1.54 | up | 8.48e-03 |
|  | C10orf99 | 1192.58 | 2114.69 | 1.79 | down | 8.50e-03 |
|  | IYD | 701.58 | 1098.12 | 1.56 | down | 8.50e-03 |
|  | AIFM3 | 622.77 | 984.67 | 1.59 | down | 8.59e-03 |
|  | CPS1 | 2479.31 | 402.41 | 6.16 | up | 8.81e-03 |
|  | CYP27A1 | 819.81 | 1216.66 | 1.49 | down | 8.91e-03 |
|  | HAGLR | 455.15 | 282.13 | 1.61 | up | 9.03e-03 |
|  | HLA-DRB1 | 12077 | 6716.09 | 1.8 | up | 9.10e-03 |
|  | TSPAN33 | 356.31 | 527.56 | 1.47 | down | 9.14e-03 |
|  | HLA-DMA | 2343.69 | 1381.64 | 1.7 | up | 9.14e-03 |
|  | ZNF649 | 144.92 | 213.51 | 1.47 | down | 9.17e-03 |
|  | BMP7 | 610.5 | 1183.22 | 1.92 | down | 9.22e-03 |
|  | ICAM1 | 1756.65 | 1131.13 | 1.55 | up | 9.24e-03 |
|  | GYG2 | 353.46 | 552.72 | 1.56 | down | 9.26e-03 |
|  | SECTM1 | 1410.12 | 648.68 | 2.17 | up | 9.26e-03 |
|  | SOWAHA | 282.46 | 463.31 | 1.64 | down | 9.48e-03 |
|  | AGAP2 | 288.62 | 156.32 | 1.85 | up | 9.53e-03 |
|  | ZNF618 | 598.46 | 380.27 | 1.57 | up | 9.53e-03 |
|  | PFKP | 5542.62 | 3850.91 | 1.44 | up | 9.58e-03 |
|  | SLC1A3 | 202.62 | 127.76 | 1.59 | up | 9.68e-03 |
|  | AGR2 | 39450.46 | 25748.78 | 1.53 | up | 9.68e-03 |
|  | SLC51B | 81.85 | 129.06 | 1.59 | down | 9.70e-03 |
|  | SLC4A4 | 594.77 | 323.08 | 1.84 | up | 9.73e-03 |
|  | CPNE1 | 4346.12 | 6402.72 | 1.47 | down | 9.80e-03 |
|  | CD247 | 170.31 | 115.99 | 1.47 | up | 9.90e-03 |
|  | IL22RA1 | 998.96 | 1445.15 | 1.45 | down | 9.91e-03 |
|  | TRIM22 | 1304.81 | 806.87 | 1.62 | up | 1.00e-02 |
| Head and neck cancer | MYO7A | 442.92 | 228.38 | 1.94 | up | 1.21e-04 |
|  | TSPAN13 | 601.25 | 1327.37 | 2.22 | down | 4.46e-04 |
|  | TRPS1 | 1619.33 | 946.36 | 1.71 | up | 5.12e-04 |
|  | HIST1H4I | 129.58 | 303.37 | 2.33 | down | 8.78e-04 |
|  | NLRC5 | 3283.92 | 2079.62 | 1.58 | up | 1.08e-03 |
|  | MOXD1 | 2289.33 | 718.87 | 3.18 | up | 1.71e-03 |
|  | ACSL5 | 1275.67 | 650.89 | 1.96 | up | 1.90e-03 |
|  | PARP3 | 768.17 | 466.17 | 1.65 | up | 2.09e-03 |
|  | ATP9B | 574.25 | 397.62 | 1.44 | up | 2.19e-03 |
|  | HIST1H2BK | 386.08 | 847.8 | 2.17 | down | 2.29e-03 |
|  | ID2 | 1339.42 | 701.35 | 1.91 | up | 2.46e-03 |
|  | POLR3G | 321.92 | 215.73 | 1.49 | up | 3.26e-03 |
|  | LRRC1 | 746.5 | 1174.57 | 1.56 | down | 3.29e-03 |
|  | MTRNR2L1 | 2.08 | 232.32 | 100 | down | 4.19e-03 |
|  | DUSP9 | 69.67 | 243.63 | 3.45 | down | 5.38e-03 |
|  | HHLA3 | 83.08 | 140.93 | 1.69 | down | 5.59e-03 |
|  | PAIP2B | 120.25 | 217.01 | 1.82 | down | 5.69e-03 |
|  | ZNF488 | 51.92 | 139.28 | 2.7 | down | 5.77e-03 |
|  | SNN | 880.33 | 1275.93 | 1.45 | down | 5.95e-03 |
|  | CCNQ | 481.5 | 696.12 | 1.45 | down | 5.99e-03 |
|  | TPPP3 | 2680.42 | 1573.33 | 1.7 | up | 6.12e-03 |
|  | PRODH | 94.58 | 278.13 | 2.94 | down | 6.33e-03 |
|  | CLDN1 | 4636.25 | 11269.34 | 2.44 | down | 6.33e-03 |
|  | PUM3 | 1656.25 | 1113.56 | 1.49 | up | 6.49e-03 |
|  | C1orf115 | 119 | 288.76 | 2.44 | down | 6.51e-03 |
|  | PRF1 | 844.5 | 407.16 | 2.07 | up | 6.61e-03 |
|  | SLC39A6 | 5258.5 | 3239.54 | 1.62 | up | 6.80e-03 |
|  | SPTSSA | 1166.58 | 1834.13 | 1.56 | down | 6.82e-03 |
|  | HIST1H3H | 56.5 | 136.15 | 2.44 | down | 7.43e-03 |
|  | METRN | 218 | 469.06 | 2.17 | down | 7.50e-03 |
|  | BOLA2-SMG1P6 | 109.58 | 169.89 | 1.54 | down | 7.80e-03 |
|  | SLC16A13 | 109.75 | 185.87 | 1.69 | down | 7.95e-03 |
|  | MAMLD1 | 86.42 | 158.01 | 1.82 | down | 8.52e-03 |
|  | DGCR6 | 52 | 106.06 | 2.04 | down | 8.83e-03 |
|  | OSBP2 | 134.17 | 286.8 | 2.13 | down | 9.46e-03 |
| Lung Adenocarcinoma | SPAG7 | 762.04 | 1107.28 | 1.45 | down | 7.94e-06 |
|  | EPB41L4A-AS1 | 193.74 | 324.15 | 1.67 | down | 1.21e-05 |
|  | TFRC | 7225.74 | 4201.62 | 1.72 | up | 4.96e-05 |
|  | NUP205 | 2813.22 | 1869.98 | 1.5 | up | 6.10e-05 |
|  | SIL1 | 1399.78 | 2077.52 | 1.49 | down | 7.68e-05 |
|  | LRAT | 43.17 | 148.78 | 3.45 | down | 7.81e-05 |
|  | IPPK | 165.91 | 109.68 | 1.51 | up | 9.65e-05 |
|  | SMOC1 | 235.7 | 691.25 | 2.94 | down | 1.07e-04 |
|  | CST3 | 5871.04 | 9597.12 | 1.64 | down | 1.39e-04 |
|  | MED1 | 2289.26 | 1419.24 | 1.61 | up | 1.50e-04 |
|  | XYLB | 237.04 | 162.85 | 1.46 | up | 1.71e-04 |
|  | PEG10 | 11377.78 | 3363.1 | 3.38 | up | 1.93e-04 |
|  | LGALS3BP | 21618.22 | 33500.78 | 1.54 | down | 2.19e-04 |
|  | NCAPD3 | 1420.43 | 916.39 | 1.55 | up | 2.20e-04 |
|  | CENPQ | 276 | 191.74 | 1.44 | up | 2.48e-04 |
|  | COQ8B | 700.78 | 1009.93 | 1.45 | down | 2.57e-04 |
|  | SLC25A13 | 1507.87 | 1003.8 | 1.5 | up | 2.63e-04 |
|  | MAGEA6 | 531.65 | 211.76 | 2.51 | up | 3.40e-04 |
|  | ADAM17 | 1768.39 | 1100.59 | 1.61 | up | 3.43e-04 |
|  | CAPZA2 | 8832.48 | 4466.79 | 1.98 | up | 3.74e-04 |
|  | ASCC3 | 2251.65 | 1513.69 | 1.49 | up | 3.85e-04 |
|  | GPRC5C | 1444.83 | 2770.04 | 1.92 | down | 3.86e-04 |
|  | ABHD14A | 265.83 | 424.79 | 1.59 | down | 3.87e-04 |
|  | SAT2 | 612.7 | 883.66 | 1.45 | down | 3.89e-04 |
|  | PDE4D | 728.13 | 1758.85 | 2.44 | down | 4.07e-04 |
|  | SLC22A3 | 320.61 | 938.38 | 2.94 | down | 4.14e-04 |
|  | KBTBD11 | 175.09 | 370.01 | 2.13 | down | 4.16e-04 |
|  | CHST10 | 210.91 | 372.56 | 1.75 | down | 4.29e-04 |
|  | TMEM38A | 353.04 | 233.24 | 1.51 | up | 4.36e-04 |
|  | RILP | 243.74 | 374.16 | 1.54 | down | 4.44e-04 |
|  | WASHC5 | 2471.39 | 1709.16 | 1.45 | up | 5.03e-04 |
|  | FAM135A | 900.7 | 623.9 | 1.44 | up | 5.08e-04 |
|  | TOPBP1 | 1773.83 | 1190.14 | 1.49 | up | 5.23e-04 |
|  | CDK12 | 3739.61 | 1963.82 | 1.9 | up | 5.42e-04 |
|  | NAA38 | 616.3 | 970.51 | 1.56 | down | 5.45e-04 |
|  | NDUFA2 | 966.91 | 1425.28 | 1.47 | down | 5.78e-04 |
|  | ZNF714 | 699.78 | 298.06 | 2.35 | up | 5.95e-04 |
|  | CIP2A | 432.87 | 258.87 | 1.67 | up | 6.26e-04 |
|  | GCLM | 2713.09 | 1480.61 | 1.83 | up | 6.45e-04 |
|  | BOLA2-SMG1P6 | 168.87 | 115.47 | 1.46 | up | 6.71e-04 |
|  | SPDYE3 | 267.43 | 184.42 | 1.45 | up | 6.96e-04 |
|  | CASTOR3 | 489.39 | 337.36 | 1.45 | up | 7.46e-04 |
|  | MSRA | 212.35 | 311.49 | 1.47 | down | 8.78e-04 |
|  | TXNRD2 | 616 | 905.17 | 1.47 | down | 8.94e-04 |
|  | NCK1 | 1252 | 869.49 | 1.44 | up | 9.06e-04 |
|  | CLTB | 1227.65 | 1837.73 | 1.49 | down | 9.25e-04 |
|  | INHA | 11.96 | 341.69 | 33.33 | down | 9.57e-04 |
|  | RANGRF | 223.91 | 344.32 | 1.54 | down | 1.03e-03 |
|  | FURIN | 5846.26 | 11288.02 | 1.92 | down | 1.06e-03 |
|  | PIP4K2C | 3296.13 | 2242.72 | 1.47 | up | 1.07e-03 |
|  | NEIL1 | 282.83 | 463.21 | 1.64 | down | 1.15e-03 |
|  | TTK | 451.65 | 288.85 | 1.56 | up | 1.16e-03 |
|  | NUP155 | 2263.7 | 1518.19 | 1.49 | up | 1.19e-03 |
|  | AZIN2 | 150.74 | 252.16 | 1.67 | down | 1.28e-03 |
|  | XRCC2 | 299.91 | 187.78 | 1.6 | up | 1.29e-03 |
|  | FBXO45 | 970.3 | 659.25 | 1.47 | up | 1.29e-03 |
|  | TRIP13 | 908.13 | 604.4 | 1.5 | up | 1.40e-03 |
|  | LMO4 | 936 | 1487.74 | 1.59 | down | 1.45e-03 |
|  | ATAD2 | 1999.3 | 1331.84 | 1.5 | up | 1.51e-03 |
|  | MCM2 | 2522.3 | 1522.1 | 1.66 | up | 1.53e-03 |
|  | TCIM | 3943.39 | 8668.05 | 2.22 | down | 1.54e-03 |
|  | RND3 | 1498.91 | 953.25 | 1.57 | up | 1.56e-03 |
|  | ARHGEF34P | 158.04 | 103.55 | 1.53 | up | 1.58e-03 |
|  | ACAP2 | 2096.83 | 1458.86 | 1.44 | up | 1.58e-03 |
|  | MAPK8IP2 | 550.26 | 276.36 | 1.99 | up | 1.59e-03 |
|  | TRIM52-AS1 | 124.39 | 179.09 | 1.45 | down | 1.66e-03 |
|  | OBSL1 | 1275.57 | 2294.62 | 1.79 | down | 1.76e-03 |
|  | TMEM45B | 2548.22 | 1598.22 | 1.59 | up | 1.81e-03 |
|  | GNE | 1136.35 | 787.25 | 1.44 | up | 1.92e-03 |
|  | BBX | 2434.48 | 1585.54 | 1.54 | up | 1.94e-03 |
|  | CKAP2L | 461 | 293.29 | 1.57 | up | 1.97e-03 |
|  | SLC16A4 | 437.91 | 1113.98 | 2.56 | down | 1.97e-03 |
|  | TSPAN8 | 1604.13 | 2549.42 | 1.59 | down | 2.00e-03 |
|  | OSGIN2 | 1604.52 | 1046.9 | 1.53 | up | 2.19e-03 |
|  | MCM10 | 446.57 | 253.82 | 1.76 | up | 2.22e-03 |
|  | ZSCAN21 | 231.26 | 141.63 | 1.63 | up | 2.29e-03 |
|  | ZKSCAN1 | 3554.39 | 2101.83 | 1.69 | up | 2.37e-03 |
|  | CASC3 | 3899.65 | 2679.74 | 1.46 | up | 2.40e-03 |
|  | BORA | 228.83 | 148.97 | 1.54 | up | 2.50e-03 |
|  | SLC35A3 | 1907.39 | 1276.66 | 1.49 | up | 2.71e-03 |
|  | IFITM2 | 2379.96 | 3471.93 | 1.45 | down | 2.75e-03 |
|  | NXPE3 | 791.04 | 524.21 | 1.51 | up | 2.93e-03 |
|  | ZBTB10 | 2003.09 | 1240.71 | 1.61 | up | 3.01e-03 |
|  | BRCA1 | 826.74 | 453.73 | 1.82 | up | 3.01e-03 |
|  | IFRD1 | 1719.61 | 1192.87 | 1.44 | up | 3.08e-03 |
|  | CRYBG3 | 959.57 | 590.58 | 1.62 | up | 3.09e-03 |
|  | LOC101929705 | 319.43 | 121.76 | 2.62 | up | 3.13e-03 |
|  | TNFRSF10D | 220.91 | 350.56 | 1.59 | down | 3.19e-03 |
|  | CDC6 | 1155.74 | 592.26 | 1.95 | up | 3.20e-03 |
|  | LY6K | 624.39 | 309.08 | 2.02 | up | 3.21e-03 |
|  | SERPINF2 | 243.74 | 580.86 | 2.38 | down | 3.22e-03 |
|  | POLQ | 285.91 | 174.44 | 1.64 | up | 3.28e-03 |
|  | CLSPN | 307.3 | 212.12 | 1.45 | up | 3.30e-03 |
|  | TFF3 | 1147.26 | 3491.02 | 3.03 | down | 3.31e-03 |
|  | SCD | 19749.61 | 11439.75 | 1.73 | up | 3.34e-03 |
|  | CREB3L1 | 2058.35 | 3042.84 | 1.47 | down | 3.42e-03 |
|  | WNK1 | 10729.57 | 7442.16 | 1.44 | up | 3.45e-03 |
|  | SMIM10L2A | 138.26 | 314.25 | 2.27 | down | 3.48e-03 |
|  | GRHL1 | 1027.22 | 664.27 | 1.55 | up | 3.49e-03 |
|  | FAM3C | 2738.22 | 1892.99 | 1.45 | up | 3.53e-03 |
|  | MAGEA3 | 680.39 | 263.61 | 2.58 | up | 3.53e-03 |
|  | CLDN7 | 3073.04 | 4468.27 | 1.45 | down | 3.54e-03 |
|  | PYCARD | 391.26 | 603.32 | 1.54 | down | 3.63e-03 |
|  | TOP2A | 5568.83 | 3331.93 | 1.67 | up | 3.73e-03 |
|  | FRS2 | 2088.17 | 879.6 | 2.37 | up | 3.75e-03 |
|  | KLF8 | 387.39 | 257.37 | 1.51 | up | 3.81e-03 |
|  | RPH3AL | 547.26 | 802.32 | 1.47 | down | 3.98e-03 |
|  | GOLGA6L9 | 56.87 | 105.26 | 1.85 | down | 4.15e-03 |
|  | RAD51AP1 | 412.87 | 269.66 | 1.53 | up | 4.25e-03 |
|  | GSPT2 | 182.96 | 293.38 | 1.61 | down | 4.30e-03 |
|  | KLC3 | 209.57 | 136.8 | 1.53 | up | 4.33e-03 |
|  | SPIN4 | 443.26 | 300.6 | 1.47 | up | 4.37e-03 |
|  | KLHDC10 | 2637.61 | 1787.67 | 1.48 | up | 4.37e-03 |
|  | PALM | 316.26 | 483.82 | 1.54 | down | 4.43e-03 |
|  | SOX11 | 9.35 | 159.12 | 16.67 | down | 4.51e-03 |
|  | CHRD | 207.48 | 396.1 | 1.92 | down | 4.78e-03 |
|  | BAHCC1 | 639.61 | 970.48 | 1.52 | down | 4.85e-03 |
|  | GJB1 | 252.17 | 595.9 | 2.38 | down | 4.89e-03 |
|  | CNNM4 | 2067.3 | 1189.75 | 1.74 | up | 4.92e-03 |
|  | COL27A1 | 510.7 | 784.12 | 1.54 | down | 5.06e-03 |
|  | TMEM38B | 534.04 | 353.37 | 1.51 | up | 5.11e-03 |
|  | SMC4 | 2195.13 | 1494.57 | 1.47 | up | 5.17e-03 |
|  | ID1 | 793 | 1640.68 | 2.08 | down | 5.48e-03 |
|  | TMEM108 | 172.26 | 350.71 | 2.04 | down | 5.49e-03 |
|  | PMM1 | 779.83 | 1258.51 | 1.61 | down | 5.69e-03 |
|  | CEP55 | 943.74 | 634.81 | 1.49 | up | 5.79e-03 |
|  | RHOBTB3 | 4073.83 | 2494.68 | 1.63 | up | 5.98e-03 |
|  | SYDE2 | 249.22 | 173.22 | 1.44 | up | 6.00e-03 |
|  | FAM149A | 163.91 | 286.27 | 1.75 | down | 6.11e-03 |
|  | ZNF367 | 262.09 | 173.65 | 1.51 | up | 6.18e-03 |
|  | KPNA2 | 4623.04 | 2925.8 | 1.58 | up | 6.19e-03 |
|  | MAGEA12 | 377.91 | 141.75 | 2.67 | up | 6.38e-03 |
|  | STARD3 | 3176.91 | 1607.87 | 1.98 | up | 6.44e-03 |
|  | TST | 992.96 | 1459.37 | 1.47 | down | 6.59e-03 |
|  | ZNF681 | 283.57 | 186.54 | 1.52 | up | 6.61e-03 |
|  | MCM6 | 2208 | 1508.25 | 1.46 | up | 6.63e-03 |
|  | PRR11 | 754.26 | 482.17 | 1.56 | up | 6.72e-03 |
|  | PLD3 | 8600.39 | 13066.98 | 1.52 | down | 6.78e-03 |
|  | MELK | 636.35 | 433.42 | 1.47 | up | 6.79e-03 |
|  | NFE2L3 | 809.04 | 1329.05 | 1.64 | down | 6.88e-03 |
|  | CHL1 | 381 | 1042.38 | 2.7 | down | 6.93e-03 |
|  | PNPLA7 | 217.96 | 356.59 | 1.64 | down | 7.03e-03 |
|  | RPS26 | 2590.96 | 3796.25 | 1.47 | down | 7.39e-03 |
|  | HILPDA | 1750.78 | 745.48 | 2.35 | up | 7.46e-03 |
|  | SLC4A3 | 131.83 | 239.13 | 1.82 | down | 7.67e-03 |
|  | WIPF2 | 3304.3 | 2278.12 | 1.45 | up | 7.89e-03 |
|  | ENGASE | 614.65 | 922.68 | 1.49 | down | 7.91e-03 |
|  | FAM177B | 28.87 | 133.34 | 4.55 | down | 8.26e-03 |
|  | SPINK5 | 404.48 | 815.71 | 2 | down | 8.31e-03 |
|  | DENND4C | 1855.35 | 1234.93 | 1.5 | up | 8.34e-03 |
|  | ABO | 674.09 | 1070.02 | 1.59 | down | 8.34e-03 |
|  | ACOX2 | 253.7 | 403.05 | 1.59 | down | 8.38e-03 |
|  | CEP72 | 340.74 | 236.58 | 1.44 | up | 8.43e-03 |
|  | SPDEF | 595.35 | 1201.87 | 2 | down | 8.47e-03 |
|  | CCNE2 | 214.35 | 148.17 | 1.45 | up | 8.54e-03 |
|  | LONRF2 | 264.57 | 438.51 | 1.67 | down | 8.63e-03 |
|  | SYT17 | 934.96 | 601.02 | 1.56 | up | 8.73e-03 |
|  | TFF1 | 70.43 | 1427.75 | 20 | down | 8.87e-03 |
|  | EGFL7 | 870.74 | 1331.58 | 1.54 | down | 8.95e-03 |
|  | KNDC1 | 149 | 379.59 | 2.56 | down | 8.97e-03 |
|  | AGA | 699.3 | 1020.7 | 1.45 | down | 9.11e-03 |
|  | WFDC2 | 6235.96 | 10811.87 | 1.72 | down | 9.14e-03 |
|  | DDIAS | 263.78 | 180.84 | 1.46 | up | 9.22e-03 |
|  | TMEM80 | 395.52 | 587.35 | 1.49 | down | 9.66e-03 |
|  | CTSV | 504.43 | 251.48 | 2.01 | up | 9.70e-03 |
|  | CSAG1 | 267.13 | 103.81 | 2.57 | up | 9.87e-03 |
|  | MAST4 | 1002.17 | 1447.85 | 1.45 | down | 9.91e-03 |
|  | KCTD1 | 261.61 | 377.43 | 1.45 | down | 1.00e-02 |
| Lung Squamous Cell Carcinoma | NOCT | 148.42 | 252.51 | 1.69 | down | 4.64e-04 |
|  | LMO4 | 5201.79 | 3063.54 | 1.7 | up | 1.57e-03 |
|  | MUC4 | 7570.37 | 2953.8 | 2.56 | up | 1.63e-03 |
|  | ARRDC2 | 507.58 | 768.38 | 1.52 | down | 1.65e-03 |
|  | BMP3 | 869.37 | 414.73 | 2.1 | up | 2.49e-03 |
|  | ACVR2A | 711.84 | 466.32 | 1.53 | up | 2.54e-03 |
|  | SGF29 | 598.58 | 362.07 | 1.65 | up | 2.68e-03 |
|  | RPS6KA6 | 336.42 | 195.41 | 1.72 | up | 3.09e-03 |
|  | KATNAL2 | 144.58 | 100.74 | 1.44 | up | 3.15e-03 |
|  | LINC01139 | 187.79 | 106.43 | 1.76 | up | 3.46e-03 |
|  | MLLT11 | 1532.16 | 997.55 | 1.54 | up | 3.46e-03 |
|  | HOXB4 | 56.32 | 126.71 | 2.27 | down | 3.96e-03 |
|  | DEGS1 | 3435 | 2331.03 | 1.47 | up | 4.45e-03 |
|  | PRKG2 | 234.05 | 134.12 | 1.75 | up | 4.58e-03 |
|  | AP3B2 | 376.89 | 259.31 | 1.45 | up | 4.58e-03 |
|  | CHST9 | 780.26 | 323.72 | 2.41 | up | 4.87e-03 |
|  | HLA-H | 529.16 | 933.21 | 1.75 | down | 5.16e-03 |
|  | KIAA0895 | 417 | 274.69 | 1.52 | up | 5.24e-03 |
|  | SULT1E1 | 499.74 | 153.3 | 3.26 | up | 5.52e-03 |
|  | CABLES1 | 502.37 | 327.36 | 1.53 | up | 5.80e-03 |
|  | FAM184A | 166.26 | 109.51 | 1.52 | up | 7.11e-03 |
|  | LYPD6B | 739.68 | 458.59 | 1.61 | up | 7.71e-03 |
|  | ERFE | 208.84 | 119.95 | 1.74 | up | 7.81e-03 |
|  | TTC39C | 1134.68 | 768.15 | 1.48 | up | 8.06e-03 |
|  | BCHE | 537.95 | 294.68 | 1.83 | up | 8.30e-03 |
| Multiple Myeloma | ZNF74 | 170.74 | 259.85 | 1.52 | down | 7.19e-05 |
|  | SLC44A1 | 5038.03 | 7287.65 | 1.45 | down | 2.78e-04 |
|  | PDE1B | 525.16 | 308.27 | 1.7 | up | 3.55e-04 |
|  | MTHFD1L | 487.48 | 794.25 | 1.64 | down | 7.54e-04 |
|  | SEPTIN10 | 1265.84 | 2230.48 | 1.75 | down | 7.81e-04 |
|  | UBE2QL1 | 840.97 | 1433.41 | 1.69 | down | 8.68e-04 |
|  | ZFAT | 282.9 | 464.61 | 1.64 | down | 1.07e-03 |
|  | SSPN | 392.32 | 704.49 | 1.79 | down | 1.20e-03 |
|  | AGPAT5 | 370.71 | 550.06 | 1.49 | down | 1.61e-03 |
|  | FAM229A | 317.42 | 212.43 | 1.49 | up | 1.71e-03 |
|  | POU4F1 | 425.39 | 219.35 | 1.94 | up | 1.81e-03 |
|  | FOXP1 | 795.61 | 480.02 | 1.66 | up | 2.33e-03 |
|  | BAG3 | 682.77 | 448.11 | 1.52 | up | 2.72e-03 |
|  | TMEM255A | 481.52 | 284.6 | 1.69 | up | 2.93e-03 |
|  | REM2 | 176.03 | 107.43 | 1.64 | up | 3.68e-03 |
|  | PACSIN3 | 78.29 | 133.81 | 1.69 | down | 3.84e-03 |
|  | HAUS3 | 1876.26 | 1261.42 | 1.49 | up | 4.42e-03 |
|  | PTPRB | 227.42 | 148.29 | 1.53 | up | 4.50e-03 |
|  | PLEKHG2 | 2395.06 | 1434.31 | 1.67 | up | 4.50e-03 |
|  | ASH1L-AS1 | 155.39 | 107.88 | 1.44 | up | 4.64e-03 |
|  | ZAP70 | 203.39 | 139.31 | 1.46 | up | 4.71e-03 |
|  | ALDH7A1 | 151.55 | 249.04 | 1.64 | down | 4.72e-03 |
|  | CXCR4 | 9765.84 | 15952.92 | 1.64 | down | 5.17e-03 |
|  | RRAD | 420.32 | 209.18 | 2.01 | up | 5.41e-03 |
|  | CLCN4 | 125.52 | 220.2 | 1.75 | down | 5.47e-03 |
|  | ZC3HAV1L | 78.65 | 132.28 | 1.69 | down | 6.08e-03 |
|  | NFKBIA | 34982.74 | 22264.15 | 1.57 | up | 6.08e-03 |
|  | HYPK | 381.23 | 259.57 | 1.47 | up | 6.17e-03 |
|  | TP53RK | 260.65 | 378.48 | 1.45 | down | 6.54e-03 |
|  | CA14 | 164.42 | 105.19 | 1.56 | up | 6.87e-03 |
|  | PSAT1 | 2535.23 | 3906.73 | 1.54 | down | 7.60e-03 |
|  | ZBTB10 | 2337.55 | 1385.34 | 1.69 | up | 7.66e-03 |
|  | BACH2 | 665.39 | 425.36 | 1.56 | up | 7.93e-03 |
|  | NR1D1 | 1670.65 | 1138.63 | 1.47 | up | 8.17e-03 |
|  | INF2 | 511.81 | 871.59 | 1.69 | down | 8.54e-03 |
|  | GEM | 717.35 | 303.43 | 2.36 | up | 8.55e-03 |
|  | CD83 | 449.19 | 282.06 | 1.59 | up | 8.73e-03 |
|  | SSTR3 | 1264.97 | 643.73 | 1.97 | up | 9.23e-03 |
|  | ZNF234 | 97.77 | 140.93 | 1.45 | down | 9.44e-03 |
|  | ZNF74 | 170.74 | 259.85 | 1.52 | down | 7.19e-05 |
|  | SLC44A1 | 5038.03 | 7287.65 | 1.45 | down | 2.78e-04 |
|  | PDE1B | 525.16 | 308.27 | 1.7 | up | 3.55e-04 |
|  | MTHFD1L | 487.48 | 794.25 | 1.64 | down | 7.54e-04 |
|  | SEPTIN10 | 1265.84 | 2230.48 | 1.75 | down | 7.81e-04 |
|  | UBE2QL1 | 840.97 | 1433.41 | 1.69 | down | 8.68e-04 |
|  | ZFAT | 282.9 | 464.61 | 1.64 | down | 1.07e-03 |
|  | SSPN | 392.32 | 704.49 | 1.79 | down | 1.20e-03 |
|  | AGPAT5 | 370.71 | 550.06 | 1.49 | down | 1.61e-03 |
|  | FAM229A | 317.42 | 212.43 | 1.49 | up | 1.71e-03 |
|  | POU4F1 | 425.39 | 219.35 | 1.94 | up | 1.81e-03 |
|  | FOXP1 | 795.61 | 480.02 | 1.66 | up | 2.33e-03 |
|  | BAG3 | 682.77 | 448.11 | 1.52 | up | 2.72e-03 |
|  | TMEM255A | 481.52 | 284.6 | 1.69 | up | 2.93e-03 |
|  | REM2 | 176.03 | 107.43 | 1.64 | up | 3.68e-03 |
|  | PACSIN3 | 78.29 | 133.81 | 1.69 | down | 3.84e-03 |
|  | HAUS3 | 1876.26 | 1261.42 | 1.49 | up | 4.42e-03 |
|  | PTPRB | 227.42 | 148.29 | 1.53 | up | 4.50e-03 |
|  | PLEKHG2 | 2395.06 | 1434.31 | 1.67 | up | 4.50e-03 |
|  | ASH1L-AS1 | 155.39 | 107.88 | 1.44 | up | 4.64e-03 |
|  | ZAP70 | 203.39 | 139.31 | 1.46 | up | 4.71e-03 |
|  | ALDH7A1 | 151.55 | 249.04 | 1.64 | down | 4.72e-03 |
|  | CXCR4 | 9765.84 | 15952.92 | 1.64 | down | 5.17e-03 |
|  | RRAD | 420.32 | 209.18 | 2.01 | up | 5.41e-03 |
|  | CLCN4 | 125.52 | 220.2 | 1.75 | down | 5.47e-03 |
|  | ZC3HAV1L | 78.65 | 132.28 | 1.69 | down | 6.08e-03 |
|  | NFKBIA | 34982.74 | 22264.15 | 1.57 | up | 6.08e-03 |
|  | HYPK | 381.23 | 259.57 | 1.47 | up | 6.17e-03 |
|  | TP53RK | 260.65 | 378.48 | 1.45 | down | 6.54e-03 |
|  | CA14 | 164.42 | 105.19 | 1.56 | up | 6.87e-03 |
|  | PSAT1 | 2535.23 | 3906.73 | 1.54 | down | 7.60e-03 |
|  | ZBTB10 | 2337.55 | 1385.34 | 1.69 | up | 7.66e-03 |
|  | BACH2 | 665.39 | 425.36 | 1.56 | up | 7.93e-03 |
|  | NR1D1 | 1670.65 | 1138.63 | 1.47 | up | 8.17e-03 |
|  | INF2 | 511.81 | 871.59 | 1.69 | down | 8.54e-03 |
|  | GEM | 717.35 | 303.43 | 2.36 | up | 8.55e-03 |
|  | CD83 | 449.19 | 282.06 | 1.59 | up | 8.73e-03 |
|  | SSTR3 | 1264.97 | 643.73 | 1.97 | up | 9.23e-03 |
|  | ZNF234 | 97.77 | 140.93 | 1.45 | down | 9.44e-03 |
| Melanoma | HDAC9 | 713.63 | 267.91 | 2.66 | up | 3.35e-06 |
|  | GPAA1 | 4067.54 | 5950.55 | 1.47 | down | 1.46e-05 |
|  | MLF1 | 523.8 | 333.1 | 1.57 | up | 2.08e-05 |
|  | NPHP1 | 187.83 | 130.22 | 1.44 | up | 3.83e-05 |
|  | HGH1 | 1184.14 | 1732.88 | 1.47 | down | 3.84e-05 |
|  | ELMOD2 | 849.89 | 591.85 | 1.44 | up | 3.96e-05 |
|  | MNS1 | 187.6 | 117.04 | 1.6 | up | 5.33e-05 |
|  | ZBTB18 | 669.91 | 456.43 | 1.47 | up | 6.86e-05 |
|  | WDR72 | 271.11 | 92.87 | 2.92 | up | 7.22e-05 |
|  | PDLIM3 | 2194.37 | 966.18 | 2.27 | up | 8.82e-05 |
|  | PSMC3IP | 275 | 189.93 | 1.45 | up | 9.01e-05 |
|  | PDE10A | 249.09 | 162.18 | 1.54 | up | 1.08e-04 |
|  | USP1 | 1829.66 | 1236.5 | 1.48 | up | 1.30e-04 |
|  | GNAZ | 282.57 | 136.06 | 2.08 | up | 1.54e-04 |
|  | RFC4 | 853.63 | 571.92 | 1.49 | up | 1.81e-04 |
|  | HOXC8 | 74.54 | 127.51 | 1.72 | down | 1.87e-04 |
|  | NCAPG2 | 1774.77 | 1194.97 | 1.49 | up | 2.09e-04 |
|  | THEM6 | 849.34 | 1268.73 | 1.49 | down | 2.45e-04 |
|  | SNX7 | 724.97 | 504.61 | 1.44 | up | 3.18e-04 |
|  | KCTD14 | 60.77 | 109.22 | 1.79 | down | 3.28e-04 |
|  | SEMA4C | 4690.71 | 2988.14 | 1.57 | up | 3.94e-04 |
|  | CCDC138 | 203.54 | 130.64 | 1.56 | up | 4.18e-04 |
|  | CASP8AP2 | 599.66 | 416.65 | 1.44 | up | 4.88e-04 |
|  | PHLDA3 | 2344.91 | 3802.64 | 1.61 | down | 5.38e-04 |
|  | CGREF1 | 230.23 | 426.25 | 1.85 | down | 5.70e-04 |
|  | RIN3 | 1204.17 | 1755.01 | 1.45 | down | 7.46e-04 |
|  | ECT2 | 1056.03 | 694.62 | 1.52 | up | 7.86e-04 |
|  | FAM81A | 325.74 | 185.81 | 1.75 | up | 9.03e-04 |
|  | CNN3 | 7389.2 | 5075.34 | 1.46 | up | 9.09e-04 |
|  | SDCBP | 19125.2 | 29511.21 | 1.54 | down | 9.20e-04 |
|  | CKAP2L | 551.2 | 382.33 | 1.44 | up | 1.02e-03 |
|  | FCGRT | 2435.69 | 3724.45 | 1.54 | down | 1.09e-03 |
|  | ZNF93 | 157.17 | 97.65 | 1.61 | up | 1.11e-03 |
|  | CYP27A1 | 4110.31 | 6352.55 | 1.54 | down | 1.13e-03 |
|  | ACSS1 | 1414.94 | 2056.18 | 1.45 | down | 1.20e-03 |
|  | SLC25A45 | 201.77 | 299.57 | 1.49 | down | 1.22e-03 |
|  | FDXR | 582.26 | 899.74 | 1.54 | down | 1.23e-03 |
|  | KRT14 | 687.69 | 18821.26 | 25 | down | 1.27e-03 |
|  | UHRF1 | 863.49 | 570.01 | 1.51 | up | 1.48e-03 |
|  | IFT57 | 940.23 | 571.42 | 1.65 | up | 1.50e-03 |
|  | VSIG10L | 100.4 | 224.62 | 2.22 | down | 1.51e-03 |
|  | ANLN | 2327.06 | 1621.47 | 1.44 | up | 1.51e-03 |
|  | IQCG | 342.49 | 231.43 | 1.48 | up | 1.52e-03 |
|  | SGO2 | 479.97 | 320.27 | 1.5 | up | 1.64e-03 |
|  | FAM174B | 783.94 | 1207.68 | 1.54 | down | 1.76e-03 |
|  | KIF11 | 1176.43 | 804.08 | 1.46 | up | 1.77e-03 |
|  | DKK1 | 646.4 | 235.77 | 2.74 | up | 1.85e-03 |
|  | SRGAP2C | 647.43 | 348.45 | 1.86 | up | 1.87e-03 |
|  | KIF15 | 514.54 | 295.39 | 1.74 | up | 1.89e-03 |
|  | ZNF439 | 265.14 | 182.35 | 1.45 | up | 1.90e-03 |
|  | NAPRT | 997.97 | 1450.37 | 1.45 | down | 1.90e-03 |
|  | SPA17 | 145.17 | 98.15 | 1.48 | up | 1.96e-03 |
|  | KIAA0895 | 220.09 | 151.14 | 1.46 | up | 1.97e-03 |
|  | PAIP1 | 1819.26 | 1244.32 | 1.46 | up | 2.03e-03 |
|  | SLC39A4 | 446.4 | 716.33 | 1.61 | down | 2.05e-03 |
|  | CYGB | 1339.57 | 2513.94 | 1.89 | down | 2.05e-03 |
|  | EDA2R | 194.4 | 321.34 | 1.67 | down | 2.19e-03 |
|  | TTK | 473.77 | 313.25 | 1.51 | up | 2.21e-03 |
|  | HLTF | 2129.74 | 1386.04 | 1.54 | up | 2.26e-03 |
|  | ASNS | 1193.97 | 728.98 | 1.64 | up | 2.32e-03 |
|  | SMC4 | 1992.74 | 1233.56 | 1.62 | up | 2.33e-03 |
|  | CAMK4 | 504.23 | 193.05 | 2.61 | up | 2.43e-03 |
|  | APOE | 32517.71 | 52986.22 | 1.64 | down | 2.46e-03 |
|  | DNA2 | 214.94 | 133.7 | 1.61 | up | 2.48e-03 |
|  | TNFSF9 | 110.49 | 198.45 | 1.79 | down | 2.62e-03 |
|  | COMMD2 | 1062.2 | 730.61 | 1.45 | up | 2.71e-03 |
|  | RAP1GAP | 784.74 | 1239.8 | 1.59 | down | 2.94e-03 |
|  | CHRM1 | 54.14 | 127.92 | 2.38 | down | 2.96e-03 |
|  | KLHL23 | 303.69 | 143.76 | 2.11 | up | 3.37e-03 |
|  | E2F7 | 485.49 | 314.32 | 1.54 | up | 3.39e-03 |
|  | TSKU | 926.77 | 1542.43 | 1.67 | down | 3.47e-03 |
|  | CIP2A | 502.11 | 320.65 | 1.57 | up | 3.52e-03 |
|  | CALML3 | 37.34 | 949.64 | 25 | down | 3.67e-03 |
|  | EIF4EBP3 | 109.66 | 172.57 | 1.56 | down | 3.76e-03 |
|  | NUF2 | 589.11 | 385.04 | 1.53 | up | 3.79e-03 |
|  | PER3 | 988.66 | 681.38 | 1.45 | up | 3.91e-03 |
|  | NCCRP1 | 38.2 | 448.53 | 11.11 | down | 3.91e-03 |
|  | RASGRF1 | 488.29 | 289.06 | 1.69 | up | 4.07e-03 |
|  | LOC100288637 | 266.51 | 166.45 | 1.6 | up | 4.09e-03 |
|  | CDK1 | 1138.54 | 786.44 | 1.45 | up | 4.09e-03 |
|  | ACP5 | 3884.17 | 5820.18 | 1.49 | down | 4.34e-03 |
|  | ESRP1 | 419.37 | 775.39 | 1.85 | down | 4.37e-03 |
|  | FAM111B | 373.54 | 255.45 | 1.46 | up | 4.46e-03 |
|  | EIF2A | 3189.14 | 2154.45 | 1.48 | up | 4.51e-03 |
|  | CRTAC1 | 1828.23 | 3737.96 | 2.04 | down | 4.77e-03 |
|  | EZH2 | 1041.43 | 722.64 | 1.44 | up | 4.87e-03 |
|  | NBEA | 759.77 | 402.63 | 1.89 | up | 4.91e-03 |
|  | RPS6KA2 | 5445.17 | 1147.49 | 4.75 | up | 4.98e-03 |
|  | XRCC2 | 325.71 | 213.68 | 1.52 | up | 5.37e-03 |
|  | HMMR | 545.57 | 349.06 | 1.56 | up | 5.38e-03 |
|  | ZG16B | 7.34 | 202.42 | 25 | down | 5.64e-03 |
|  | MYBPC1 | 25.4 | 115.26 | 4.55 | down | 5.76e-03 |
|  | CDKN1A | 3006.23 | 4418.53 | 1.47 | down | 5.79e-03 |
|  | PHLDB2 | 1005.14 | 437.95 | 2.3 | up | 5.94e-03 |
|  | NRG3 | 189.94 | 338.13 | 1.79 | down | 5.96e-03 |
|  | KRT13 | 121.71 | 428.65 | 3.57 | down | 5.99e-03 |
|  | TENM3 | 1056.8 | 545.21 | 1.94 | up | 6.18e-03 |
|  | ADSSL1 | 137.17 | 218.79 | 1.59 | down | 6.32e-03 |
|  | DLG3 | 609.91 | 402.55 | 1.52 | up | 6.33e-03 |
|  | PRELP | 1144.17 | 1790.97 | 1.56 | down | 6.55e-03 |
|  | MDGA2 | 92.23 | 159.07 | 1.72 | down | 6.57e-03 |
|  | ENPP4 | 284.31 | 169.88 | 1.67 | up | 6.59e-03 |
|  | VLDLR | 979.46 | 1440.7 | 1.47 | down | 6.67e-03 |
|  | MTCL1 | 419.54 | 279.48 | 1.5 | up | 6.72e-03 |
|  | CDKN2A | 1196.97 | 809.58 | 1.48 | up | 7.36e-03 |
|  | LINC02303 | 20.66 | 150.33 | 7.14 | down | 7.57e-03 |
|  | MSC | 650.54 | 1237.71 | 1.89 | down | 7.66e-03 |
|  | SAMD5 | 494.8 | 188.36 | 2.63 | up | 7.81e-03 |
|  | KRT16 | 325.49 | 11247.1 | 33.33 | down | 7.89e-03 |
|  | IL1RAP | 2779.14 | 1869.53 | 1.49 | up | 8.14e-03 |
|  | CHSY1 | 3058.11 | 2038.83 | 1.5 | up | 8.19e-03 |
|  | ASPM | 1255.37 | 847.47 | 1.48 | up | 8.20e-03 |
|  | CSRP2 | 793.6 | 339.46 | 2.34 | up | 8.27e-03 |
|  | DRAXIN | 445.77 | 117.37 | 3.8 | up | 8.30e-03 |
|  | CDR2L | 588.89 | 366.94 | 1.6 | up | 8.46e-03 |
|  | TG | 147.8 | 353.82 | 2.38 | down | 8.48e-03 |
|  | TRIM45 | 270.34 | 187.64 | 1.44 | up | 8.67e-03 |
|  | DLG5 | 964.26 | 669.43 | 1.44 | up | 8.69e-03 |
|  | SH3BGR | 191.06 | 94.84 | 2.01 | up | 8.81e-03 |
|  | LYPD1 | 722.46 | 390.93 | 1.85 | up | 9.06e-03 |
|  | MLLT11 | 1173.71 | 603.17 | 1.95 | up | 9.06e-03 |
|  | TTLL7 | 309.8 | 186.33 | 1.66 | up | 9.12e-03 |
|  | KCNS3 | 764.34 | 350.7 | 2.18 | up | 9.15e-03 |
|  | DEPDC1 | 522.03 | 332.26 | 1.57 | up | 9.24e-03 |
|  | TSHZ3 | 404.63 | 258.3 | 1.57 | up | 9.36e-03 |
|  | SLC36A4 | 443.8 | 297.85 | 1.49 | up | 9.40e-03 |
|  | HMGN5 | 310.17 | 187.82 | 1.65 | up | 9.69e-03 |
|  | LEPR | 272.31 | 155.42 | 1.75 | up | 9.78e-03 |
| Gastric cancer | TMX1 | 2412.54 | 1498.7 | 1.61 | up | 1.10e-05 |
|  | LRR1 | 428.15 | 281.47 | 1.52 | up | 8.25e-05 |
|  | HSD17B12 | 4561.85 | 2940.76 | 1.55 | up | 1.28e-04 |
|  | GNPNAT1 | 2200.54 | 1356.63 | 1.62 | up | 5.04e-04 |
|  | GCH1 | 822.23 | 487.14 | 1.69 | up | 6.18e-04 |
|  | USP2 | 36.54 | 115.19 | 3.12 | down | 7.92e-04 |
|  | ERH | 2984.92 | 1970.81 | 1.51 | up | 8.19e-04 |
|  | FEN1 | 1600.38 | 999.73 | 1.6 | up | 8.27e-04 |
|  | POLE2 | 351.31 | 227.6 | 1.54 | up | 8.39e-04 |
|  | ASF1B | 1266 | 874.15 | 1.45 | up | 8.96e-04 |
|  | CHAC2 | 191.15 | 131.87 | 1.45 | up | 9.52e-04 |
|  | TRMT5 | 665.54 | 453.62 | 1.47 | up | 1.01e-03 |
|  | TUBG2 | 169.08 | 288.01 | 1.69 | down | 1.10e-03 |
|  | MBD2 | 2748 | 1907.53 | 1.44 | up | 1.15e-03 |
|  | BTG3 | 1150.62 | 727.94 | 1.58 | up | 1.15e-03 |
|  | L2HGDH | 571.54 | 374.04 | 1.53 | up | 1.21e-03 |
|  | RAMAC | 425 | 273.26 | 1.56 | up | 1.26e-03 |
|  | MTHFD1 | 3453.62 | 2200.23 | 1.57 | up | 1.38e-03 |
|  | HMGN3 | 1704.46 | 1141.47 | 1.49 | up | 1.48e-03 |
|  | MRPL39 | 634.46 | 428.51 | 1.48 | up | 1.69e-03 |
|  | MPP2 | 46.54 | 122.93 | 2.63 | down | 1.70e-03 |
|  | STARD4 | 879.08 | 535.9 | 1.64 | up | 1.74e-03 |
|  | GPI | 14948.08 | 10229.52 | 1.46 | up | 1.77e-03 |
|  | PCSK9 | 1941.92 | 795.64 | 2.44 | up | 1.79e-03 |
|  | TMEM74B | 55.38 | 179.25 | 3.23 | down | 1.93e-03 |
|  | WDR41 | 946.77 | 650.4 | 1.46 | up | 1.94e-03 |
|  | AKAP6 | 62.38 | 251.28 | 4 | down | 2.10e-03 |
|  | RRM2 | 3631.85 | 2426.4 | 1.5 | up | 2.11e-03 |
|  | PNLIPRP2 | 11.77 | 295.55 | 25 | down | 2.14e-03 |
|  | KALRN | 691.31 | 1278.4 | 1.85 | down | 2.16e-03 |
|  | WDR89 | 461.69 | 307.13 | 1.5 | up | 2.27e-03 |
|  | H2AFZ | 5574.38 | 3489.37 | 1.6 | up | 2.28e-03 |
|  | NDRG4 | 54 | 145.97 | 2.7 | down | 2.42e-03 |
|  | CHN2 | 241 | 492.37 | 2.04 | down | 2.48e-03 |
|  | ERO1A | 7560.62 | 4934.1 | 1.53 | up | 2.56e-03 |
|  | CYP51A1 | 517.38 | 338.41 | 1.53 | up | 2.64e-03 |
|  | SLC44A3 | 1105 | 721.31 | 1.53 | up | 2.80e-03 |
|  | TPT1-AS1 | 239.92 | 445.18 | 1.85 | down | 2.86e-03 |
|  | IDI1 | 2334.46 | 1460.54 | 1.6 | up | 2.91e-03 |
|  | SOBP | 133.77 | 352.08 | 2.63 | down | 3.00e-03 |
|  | SLC39A8 | 1934.31 | 761.83 | 2.54 | up | 3.06e-03 |
|  | INSIG1 | 2984.85 | 1668.44 | 1.79 | up | 3.11e-03 |
|  | PLEKHA4 | 511.77 | 1084.34 | 2.13 | down | 3.14e-03 |
|  | ZNF367 | 624.15 | 432.52 | 1.44 | up | 3.25e-03 |
|  | FDPS | 2902.23 | 1777.08 | 1.63 | up | 3.62e-03 |
|  | DTL | 976.69 | 670.37 | 1.46 | up | 3.67e-03 |
|  | CYB561D2 | 637.15 | 433.28 | 1.47 | up | 3.69e-03 |
|  | MCUB | 615.46 | 426.93 | 1.44 | up | 3.78e-03 |
|  | UHRF1 | 1146.46 | 691.91 | 1.66 | up | 3.78e-03 |
|  | ZNF10 | 92.08 | 161.38 | 1.75 | down | 3.97e-03 |
|  | ITGA7 | 311 | 849.45 | 2.7 | down | 4.09e-03 |
|  | SPC24 | 388.23 | 251.11 | 1.55 | up | 4.23e-03 |
|  | OGDHL | 11.46 | 190.04 | 16.67 | down | 4.39e-03 |
|  | ZSCAN18 | 101.23 | 290.81 | 2.86 | down | 4.50e-03 |
|  | NNAT | 29.54 | 124.23 | 4.17 | down | 4.51e-03 |
|  | ABCB9 | 147.38 | 245 | 1.67 | down | 4.63e-03 |
|  | ENPP4 | 1190.77 | 763.52 | 1.56 | up | 4.63e-03 |
|  | ZNF750 | 8.15 | 160.55 | 20 | down | 4.72e-03 |
|  | LINC01089 | 88.62 | 147.29 | 1.67 | down | 4.98e-03 |
|  | CREB3L3 | 23.46 | 329.73 | 14.29 | down | 5.08e-03 |
|  | SHF | 82.31 | 138.74 | 1.69 | down | 5.09e-03 |
|  | TF | 9.77 | 489.89 | 50 | down | 5.10e-03 |
|  | ZNF606 | 77.69 | 182.35 | 2.33 | down | 5.13e-03 |
|  | INTU | 158.46 | 271.59 | 1.72 | down | 5.41e-03 |
|  | CCDC40 | 67.46 | 134.47 | 2 | down | 5.43e-03 |
|  | AGAP9 | 62.38 | 115.32 | 1.85 | down | 5.65e-03 |
|  | SLC2A12 | 103.08 | 211.11 | 2.04 | down | 5.79e-03 |
|  | TP53INP2 | 1324.15 | 2279.28 | 1.72 | down | 5.91e-03 |
|  | ULK1 | 1032.23 | 1496.49 | 1.45 | down | 5.93e-03 |
|  | TMED3 | 3911.85 | 2637.92 | 1.48 | up | 6.10e-03 |
|  | S1PR3 | 382.08 | 915.57 | 2.38 | down | 6.12e-03 |
|  | GTF2A2 | 2062 | 1258.37 | 1.64 | up | 6.15e-03 |
|  | DLGAP5 | 1021.46 | 640.59 | 1.59 | up | 6.71e-03 |
|  | CDKN3 | 676.92 | 447.42 | 1.51 | up | 6.84e-03 |
|  | GABRE | 158.69 | 350.1 | 2.22 | down | 7.04e-03 |
|  | NCF4 | 717.85 | 264.76 | 2.71 | up | 7.12e-03 |
|  | CASTOR2 | 78.08 | 155.06 | 2 | down | 7.17e-03 |
|  | C20orf194 | 305.62 | 573.46 | 1.89 | down | 7.41e-03 |
|  | TYMS | 1579.46 | 1043.03 | 1.51 | up | 7.43e-03 |
|  | SLC20A2 | 966.77 | 1396.16 | 1.45 | down | 7.49e-03 |
|  | PER3 | 372.23 | 741.25 | 2 | down | 7.70e-03 |
|  | RBP2 | 23.08 | 203.74 | 9.09 | down | 7.72e-03 |
|  | PARP1 | 7688.92 | 4435.58 | 1.73 | up | 8.01e-03 |
|  | ENO2 | 1098.54 | 681.4 | 1.61 | up | 8.04e-03 |
|  | FUT8 | 2525.62 | 1636.24 | 1.54 | up | 8.07e-03 |
|  | HECTD2 | 160.23 | 278.81 | 1.75 | down | 8.23e-03 |
|  | DHFR | 864.85 | 595.79 | 1.45 | up | 8.26e-03 |
|  | DEPDC1 | 814.46 | 536.34 | 1.52 | up | 8.39e-03 |
|  | FKBP3 | 1262.69 | 877.22 | 1.44 | up | 8.42e-03 |
|  | TBC1D32 | 107.54 | 155.85 | 1.45 | down | 8.45e-03 |
|  | RASL12 | 156.77 | 315.32 | 2 | down | 8.52e-03 |
|  | EFHD1 | 106.77 | 219.3 | 2.04 | down | 8.58e-03 |
|  | LMNB1 | 2783.85 | 1669.25 | 1.67 | up | 8.58e-03 |
|  | DERL3 | 966.77 | 633.38 | 1.53 | up | 8.79e-03 |
|  | WDHD1 | 656 | 424.39 | 1.55 | up | 8.92e-03 |
|  | CGNL1 | 145.54 | 398.73 | 2.7 | down | 9.09e-03 |
|  | AMOTL1 | 727.46 | 1538.27 | 2.13 | down | 9.09e-03 |
|  | CCNG1 | 3528.08 | 2372.99 | 1.49 | up | 9.20e-03 |
|  | ZNF154 | 75.85 | 148.2 | 1.96 | down | 9.23e-03 |
|  | MZT2A | 1413.08 | 950.91 | 1.49 | up | 9.31e-03 |
|  | HLF | 51.15 | 179.58 | 3.57 | down | 9.38e-03 |
|  | CCDC88C | 2869.23 | 1828.84 | 1.57 | up | 9.45e-03 |
|  | RPS19 | 21112.08 | 14094.38 | 1.5 | up | 9.71e-03 |
|  | RRAD | 66.38 | 217.19 | 3.23 | down | 9.89e-03 |
|  | C5orf30 | 720.23 | 485.24 | 1.48 | up | 1.00e-02 |
|  | AP5M1 | 1767.77 | 1188.86 | 1.49 | up | 1.00e-02 |
| Uterine cancer | NIPA1 | 809.12 | 446.96 | 1.81 | up | 2.97e-09 |
|  | CDC6 | 1197.02 | 749.78 | 1.6 | up | 2.27e-08 |
|  | DUOXA1 | 26 | 141.34 | 5.56 | down | 2.35e-08 |
|  | CORO1C | 3629.53 | 2477.24 | 1.47 | up | 2.49e-08 |
|  | CCDC3 | 322.98 | 785.79 | 2.44 | down | 6.83e-08 |
|  | UTP4 | 1520.98 | 1058.57 | 1.44 | up | 1.01e-07 |
|  | LONP1 | 6347.51 | 4340.97 | 1.46 | up | 1.07e-07 |
|  | CXCL13 | 638.91 | 281.73 | 2.27 | up | 1.09e-07 |
|  | CHAF1A | 1416.93 | 957.85 | 1.48 | up | 1.21e-07 |
|  | CORO2B | 32.09 | 124.75 | 3.85 | down | 1.25e-07 |
|  | DUOX2 | 27.93 | 116.4 | 4.17 | down | 1.61e-07 |
|  | TAF9 | 1851.28 | 1270.16 | 1.46 | up | 1.68e-07 |
|  | HASPIN | 208.28 | 116.46 | 1.79 | up | 1.74e-07 |
|  | CXCL9 | 2067.02 | 867.14 | 2.38 | up | 2.50e-07 |
|  | RAD51 | 476.74 | 315.44 | 1.51 | up | 4.12e-07 |
|  | OIP5 | 194.28 | 123.4 | 1.57 | up | 4.18e-07 |
|  | TIGAR | 375.95 | 259.41 | 1.45 | up | 4.40e-07 |
|  | ITGAE | 704.7 | 485.75 | 1.45 | up | 4.98e-07 |
|  | SKA1 | 472.51 | 309.57 | 1.53 | up | 6.38e-07 |
|  | GINS2 | 699.7 | 475.02 | 1.47 | up | 6.57e-07 |
|  | TUBGCP4 | 734.93 | 458.18 | 1.6 | up | 8.00e-07 |
|  | PKN3 | 1035.98 | 658.05 | 1.57 | up | 8.88e-07 |
|  | CYP2J2 | 156.88 | 276.4 | 1.75 | down | 9.90e-07 |
|  | TMCC2 | 58.7 | 134.58 | 2.27 | down | 1.01e-06 |
|  | SUSD2 | 465.84 | 1442.82 | 3.12 | down | 1.03e-06 |
|  | PGAM1 | 1428.6 | 919.45 | 1.55 | up | 1.13e-06 |
|  | GJB6 | 44.4 | 128.76 | 2.94 | down | 1.14e-06 |
|  | OSGIN1 | 661.21 | 272.36 | 2.43 | up | 1.29e-06 |
|  | NDRG2 | 1151.88 | 2233.57 | 1.92 | down | 1.29e-06 |
|  | IFRD1 | 1407.19 | 956.78 | 1.47 | up | 1.38e-06 |
|  | ORC1 | 404.74 | 257.95 | 1.57 | up | 1.68e-06 |
|  | ACAT1 | 1323.65 | 920.13 | 1.44 | up | 1.98e-06 |
|  | PSMC3IP | 211.88 | 130.47 | 1.62 | up | 2.06e-06 |
|  | ATP1B2 | 49.72 | 147.28 | 2.94 | down | 2.25e-06 |
|  | CENPN | 488.74 | 325.16 | 1.5 | up | 2.54e-06 |
|  | NOCT | 252.35 | 158.64 | 1.59 | up | 2.55e-06 |
|  | FAXDC2 | 200.98 | 363.62 | 1.82 | down | 2.55e-06 |
|  | WNT4 | 65.58 | 179.11 | 2.7 | down | 3.17e-06 |
|  | ZFP92 | 80.98 | 147.27 | 1.82 | down | 3.57e-06 |
|  | DUOX1 | 195.16 | 780.78 | 4 | down | 3.80e-06 |
|  | CDKN2A | 775 | 1703.53 | 2.22 | down | 3.86e-06 |
|  | PPP3CC | 405.72 | 280.76 | 1.45 | up | 3.90e-06 |
|  | GALC | 364.81 | 611.83 | 1.67 | down | 4.04e-06 |
|  | SEMA6D | 47.6 | 112.22 | 2.38 | down | 4.07e-06 |
|  | BNIP3 | 3419.65 | 1930.49 | 1.77 | up | 4.21e-06 |
|  | CPAMD8 | 400.56 | 1386.14 | 3.45 | down | 4.26e-06 |
|  | BAG2 | 811.95 | 512.58 | 1.58 | up | 4.27e-06 |
|  | AOC1 | 1364.6 | 4029.66 | 2.94 | down | 4.79e-06 |
|  | HHAT | 143.33 | 251.24 | 1.75 | down | 4.90e-06 |
|  | ACKR3 | 450.6 | 910.47 | 2.04 | down | 4.93e-06 |
|  | AP1S3 | 232.56 | 143.92 | 1.62 | up | 4.97e-06 |
|  | IFT172 | 1074.51 | 1609.09 | 1.49 | down | 5.04e-06 |
|  | KATNAL2 | 112.65 | 211.54 | 1.89 | down | 5.18e-06 |
|  | WFDC2 | 33774.05 | 68180.85 | 2 | down | 5.24e-06 |
|  | PDGFD | 190.02 | 378.98 | 2 | down | 5.30e-06 |
|  | LAG3 | 625.93 | 291.06 | 2.15 | up | 5.48e-06 |
|  | GPT2 | 4233.86 | 2745.37 | 1.54 | up | 5.48e-06 |
|  | C9orf40 | 265.05 | 179.67 | 1.48 | up | 5.59e-06 |
|  | IGSF9 | 976.56 | 1869.54 | 1.92 | down | 6.15e-06 |
|  | DIAPH3 | 550.86 | 346.57 | 1.59 | up | 6.36e-06 |
|  | PVR | 1807.53 | 1226.14 | 1.47 | up | 7.65e-06 |
|  | SLC7A4 | 80.63 | 134.07 | 1.67 | down | 8.19e-06 |
|  | WHAMM | 587.63 | 408.46 | 1.44 | up | 8.42e-06 |
|  | BRCA1 | 680.56 | 449.34 | 1.51 | up | 8.46e-06 |
|  | ZNF667 | 110.86 | 257.19 | 2.33 | down | 8.50e-06 |
|  | SLC22A17 | 383.4 | 707.46 | 1.85 | down | 8.86e-06 |
|  | WDR76 | 434.53 | 275.69 | 1.58 | up | 8.90e-06 |
|  | SHCBP1 | 490.3 | 339.62 | 1.44 | up | 8.92e-06 |
|  | PRSS12 | 181.72 | 346.86 | 1.92 | down | 9.32e-06 |
|  | PTP4A3 | 984.3 | 1830.24 | 1.85 | down | 9.65e-06 |
|  | LDHA | 27638.65 | 18956.85 | 1.46 | up | 9.98e-06 |
|  | RIMKLA | 426.88 | 252.79 | 1.69 | up | 1.01e-05 |
|  | PBXIP1 | 3340.98 | 5007.31 | 1.49 | down | 1.04e-05 |
|  | TMEM190 | 23.88 | 195.2 | 8.33 | down | 1.05e-05 |
|  | SCNN1G | 353.19 | 910.76 | 2.56 | down | 1.14e-05 |
|  | DTX4 | 585.7 | 976.05 | 1.67 | down | 1.14e-05 |
|  | RRM1 | 2603.05 | 1749.8 | 1.49 | up | 1.21e-05 |
|  | PNMA6A | 85.35 | 144.57 | 1.69 | down | 1.33e-05 |
|  | TMEM173 | 1242.44 | 1961.71 | 1.59 | down | 1.34e-05 |
|  | CDT1 | 1410.12 | 973.91 | 1.45 | up | 1.41e-05 |
|  | CDKN2B | 201.74 | 375.7 | 1.85 | down | 1.49e-05 |
|  | BRI3BP | 1437.84 | 986.38 | 1.46 | up | 1.53e-05 |
|  | MAP3K9 | 684.26 | 460.49 | 1.49 | up | 1.55e-05 |
|  | NCAPD3 | 1433.67 | 990.33 | 1.45 | up | 1.58e-05 |
|  | AUNIP | 270.42 | 182.5 | 1.48 | up | 1.60e-05 |
|  | TSPY26P | 225 | 415.36 | 1.85 | down | 1.61e-05 |
|  | NLN | 1482.4 | 1000.69 | 1.48 | up | 1.67e-05 |
|  | ERCC6L | 266.95 | 175.88 | 1.52 | up | 1.68e-05 |
|  | MYEOV | 408.93 | 217.29 | 1.88 | up | 1.91e-05 |
|  | ZNF117 | 464.23 | 821.08 | 1.75 | down | 2.28e-05 |
|  | TIAM1 | 245.21 | 433.41 | 1.75 | down | 2.29e-05 |
|  | GABBR1 | 262.79 | 523.09 | 2 | down | 2.31e-05 |
|  | PTGES | 110.56 | 260.66 | 2.38 | down | 2.50e-05 |
|  | ABO | 96.51 | 251.76 | 2.63 | down | 2.74e-05 |
|  | CDC25A | 551.53 | 376.07 | 1.47 | up | 2.84e-05 |
|  | FAM181A | 96.58 | 190.13 | 1.96 | down | 3.03e-05 |
|  | ZNF667-AS1 | 238.4 | 439.92 | 1.85 | down | 3.22e-05 |
|  | RRM2 | 2686.65 | 1563.46 | 1.72 | up | 3.23e-05 |
|  | SLC4A8 | 93.7 | 193.76 | 2.08 | down | 3.32e-05 |
|  | CST3 | 11716.86 | 18003.43 | 1.54 | down | 3.42e-05 |
|  | SLCO4A1 | 1125 | 344.82 | 3.26 | up | 3.43e-05 |
|  | TUBA4A | 2856.26 | 1675.45 | 1.7 | up | 3.67e-05 |
|  | ALDH1L1 | 176.47 | 298.84 | 1.69 | down | 3.89e-05 |
|  | ATP6V1B1 | 286.56 | 863.83 | 3.03 | down | 3.98e-05 |
|  | BCAM | 6852.84 | 17807.26 | 2.63 | down | 4.01e-05 |
|  | ZMAT1 | 152.05 | 316.55 | 2.08 | down | 4.04e-05 |
|  | CLDN9 | 91.49 | 328.96 | 3.57 | down | 4.07e-05 |
|  | GPR143 | 152.35 | 262.98 | 1.72 | down | 4.09e-05 |
|  | GCH1 | 569.6 | 353.39 | 1.61 | up | 4.16e-05 |
|  | FAM43A | 166.65 | 312.06 | 1.89 | down | 4.17e-05 |
|  | DHFR | 580.65 | 374.33 | 1.55 | up | 4.17e-05 |
|  | TMEM38B | 248.44 | 162.59 | 1.53 | up | 4.30e-05 |
|  | COL28A1 | 67.23 | 172.33 | 2.56 | down | 4.38e-05 |
|  | SCD | 12587.51 | 7756.64 | 1.62 | up | 4.58e-05 |
|  | TACR1 | 34.6 | 124.23 | 3.57 | down | 4.58e-05 |
|  | EVA1C | 188.47 | 301.36 | 1.59 | down | 4.59e-05 |
|  | ADAMTS17 | 71.07 | 141.3 | 2 | down | 4.62e-05 |
|  | MYO15B | 963.56 | 1388.05 | 1.45 | down | 4.63e-05 |
|  | RFC3 | 902.14 | 615.18 | 1.47 | up | 4.67e-05 |
|  | MYCL | 197.21 | 392.83 | 2 | down | 4.78e-05 |
|  | SCN5A | 46.42 | 106.83 | 2.33 | down | 4.83e-05 |
|  | PCAT19 | 100.07 | 326.83 | 3.23 | down | 5.02e-05 |
|  | MELK | 941.98 | 641.44 | 1.47 | up | 5.04e-05 |
|  | C1QTNF3 | 94.47 | 167.92 | 1.79 | down | 5.51e-05 |
|  | KCNQ3 | 36.72 | 123.68 | 3.33 | down | 5.63e-05 |
|  | SBDSP1 | 244.74 | 163.91 | 1.49 | up | 5.66e-05 |
|  | EFS | 216.84 | 374.77 | 1.72 | down | 5.70e-05 |
|  | SERTAD4 | 139.28 | 327.66 | 2.33 | down | 5.72e-05 |
|  | TRMT9B | 83.7 | 217.66 | 2.63 | down | 5.73e-05 |
|  | SUCLA2 | 1344 | 906.67 | 1.48 | up | 5.98e-05 |
|  | CYYR1 | 154.7 | 278.63 | 1.79 | down | 6.08e-05 |
|  | IQCA1 | 88.72 | 174.76 | 1.96 | down | 6.10e-05 |
|  | PLB1 | 84.98 | 155.26 | 1.82 | down | 6.23e-05 |
|  | LINC00284 | 60.44 | 165.44 | 2.7 | down | 6.38e-05 |
|  | USHBP1 | 72.65 | 141.64 | 1.96 | down | 6.38e-05 |
|  | MUC4 | 289.28 | 1264.38 | 4.35 | down | 6.39e-05 |
|  | LPL | 69.91 | 139.11 | 2 | down | 6.48e-05 |
|  | VGLL1 | 54.67 | 141.09 | 2.56 | down | 6.54e-05 |
|  | TLR5 | 120.28 | 229.23 | 1.92 | down | 6.57e-05 |
|  | THBS3 | 836.88 | 1333.97 | 1.59 | down | 6.89e-05 |
|  | PGK1 | 15348.19 | 10628.46 | 1.44 | up | 7.44e-05 |
|  | TYMS | 1338.26 | 904.89 | 1.48 | up | 7.54e-05 |
|  | C4orf46 | 588.79 | 405.91 | 1.45 | up | 7.76e-05 |
|  | LOC100507642 | 83.65 | 162.96 | 1.96 | down | 7.79e-05 |
|  | ENO2 | 2794.42 | 1843.78 | 1.52 | up | 7.80e-05 |
|  | RGL1 | 478.72 | 732.81 | 1.54 | down | 7.97e-05 |
|  | KCTD14 | 543.12 | 335.19 | 1.62 | up | 8.09e-05 |
|  | FBXL7 | 200.56 | 354.86 | 1.75 | down | 8.27e-05 |
|  | LTBP4 | 1660.84 | 3320.39 | 2 | down | 8.34e-05 |
|  | HSPA8 | 30187.91 | 20390.29 | 1.48 | up | 8.36e-05 |
|  | TRIM16L | 442.33 | 230.36 | 1.92 | up | 8.65e-05 |
|  | EFNA1 | 2120.3 | 3357.76 | 1.59 | down | 8.69e-05 |
|  | PRIMA1 | 85.72 | 223.58 | 2.63 | down | 8.77e-05 |
|  | UHRF1 | 1130.95 | 657.78 | 1.72 | up | 8.92e-05 |
|  | SLAMF7 | 565.58 | 334.67 | 1.69 | up | 9.48e-05 |
|  | TUBB2B | 348.7 | 537.03 | 1.54 | down | 9.66e-05 |
|  | GZMB | 464.07 | 315.98 | 1.47 | up | 9.81e-05 |
|  | FRMD4A | 265.53 | 412.45 | 1.56 | down | 9.92e-05 |
|  | GTF2IRD2B | 74.49 | 112.92 | 1.52 | down | 1.01e-04 |
|  | CDCA2 | 475.35 | 307.84 | 1.54 | up | 1.02e-04 |
|  | ARHGEF15 | 149.81 | 242.48 | 1.61 | down | 1.06e-04 |
|  | ANKMY1 | 471.56 | 714.97 | 1.52 | down | 1.07e-04 |
|  | ALPL | 2370.23 | 4524.4 | 1.92 | down | 1.07e-04 |
|  | RAMP2 | 335.09 | 531.69 | 1.59 | down | 1.08e-04 |
|  | DSTNP2 | 182.77 | 123.16 | 1.48 | up | 1.09e-04 |
|  | ADAM32 | 59.19 | 108.8 | 1.85 | down | 1.10e-04 |
|  | SLC27A6 | 99.63 | 145.72 | 1.47 | down | 1.12e-04 |
|  | NBPF1 | 475.16 | 330.24 | 1.44 | up | 1.13e-04 |
|  | ARHGEF4 | 224.7 | 377.83 | 1.69 | down | 1.14e-04 |
|  | RBPMS2 | 442.6 | 277.9 | 1.59 | up | 1.20e-04 |
|  | RBBP8 | 1964.23 | 1354.09 | 1.45 | up | 1.21e-04 |
|  | KCNK15 | 276.93 | 464.95 | 1.67 | down | 1.21e-04 |
|  | ZNF367 | 285.81 | 185.76 | 1.54 | up | 1.23e-04 |
|  | TRIM17 | 174.16 | 310.68 | 1.79 | down | 1.27e-04 |
|  | TARBP1 | 1001.05 | 1549.59 | 1.54 | down | 1.27e-04 |
|  | IGLL5 | 1721.84 | 1127.46 | 1.53 | up | 1.29e-04 |
|  | KLHDC8A | 42.09 | 143.93 | 3.45 | down | 1.31e-04 |
|  | SCNN1B | 453.6 | 1103.85 | 2.44 | down | 1.32e-04 |
|  | PPOX | 645.95 | 944.45 | 1.47 | down | 1.33e-04 |
|  | MX2 | 339.88 | 762.7 | 2.22 | down | 1.33e-04 |
|  | L3MBTL4 | 122.12 | 203.81 | 1.67 | down | 1.34e-04 |
|  | MS4A8 | 86.02 | 1052.03 | 12.5 | down | 1.43e-04 |
|  | LYPD2 | 2.63 | 186.35 | 100 | down | 1.45e-04 |
|  | LMO2 | 566.6 | 934.36 | 1.64 | down | 1.45e-04 |
|  | DHRS2 | 1468.63 | 329.67 | 4.45 | up | 1.48e-04 |
|  | FGD5 | 324.07 | 491.26 | 1.52 | down | 1.50e-04 |
|  | CLSPN | 285.05 | 172.79 | 1.65 | up | 1.56e-04 |
|  | ST6GALNAC2 | 721.98 | 1242.84 | 1.72 | down | 1.57e-04 |
|  | TNFRSF19 | 718.42 | 1444.09 | 2 | down | 1.57e-04 |
|  | DDIT4 | 6544.81 | 3624.54 | 1.81 | up | 1.58e-04 |
|  | IFI6 | 7990.74 | 13823.92 | 1.72 | down | 1.60e-04 |
|  | APBA1 | 83.07 | 126.93 | 1.54 | down | 1.64e-04 |
|  | NEBL | 193.23 | 458.88 | 2.38 | down | 1.67e-04 |
|  | COL9A2 | 1408.56 | 2826.35 | 2 | down | 1.68e-04 |
|  | B3GNT8 | 78.93 | 148.81 | 1.89 | down | 1.69e-04 |
|  | MUC1 | 10961.74 | 18280.92 | 1.67 | down | 1.69e-04 |
|  | WNT6 | 76.74 | 150.02 | 1.96 | down | 1.71e-04 |
|  | GALNT6 | 957.09 | 1693.32 | 1.75 | down | 1.75e-04 |
|  | CD8A | 689.63 | 289.05 | 2.39 | up | 1.77e-04 |
|  | NCAPG | 773.72 | 526.01 | 1.47 | up | 1.78e-04 |
|  | ABCG2 | 91.51 | 136.11 | 1.49 | down | 1.79e-04 |
|  | PBK | 776.14 | 506.66 | 1.53 | up | 1.79e-04 |
|  | TRIM29 | 467.56 | 933.99 | 2 | down | 1.79e-04 |
|  | XRCC2 | 332.02 | 230.05 | 1.44 | up | 1.80e-04 |
|  | TIGIT | 235.81 | 124.52 | 1.89 | up | 1.86e-04 |
|  | RSPO4 | 31.16 | 129.68 | 4.17 | down | 1.87e-04 |
|  | IRS2 | 234.14 | 599.33 | 2.56 | down | 1.89e-04 |
|  | LIMS2 | 564.28 | 842.83 | 1.49 | down | 1.89e-04 |
|  | TAPBPL | 641.33 | 948.02 | 1.47 | down | 1.93e-04 |
|  | FRZB | 246.23 | 779.76 | 3.12 | down | 2.00e-04 |
|  | EMID1 | 1634.84 | 3094.11 | 1.89 | down | 2.03e-04 |
|  | TMEM59L | 58.93 | 149.56 | 2.56 | down | 2.03e-04 |
|  | CXCR6 | 222.65 | 115.99 | 1.92 | up | 2.15e-04 |
|  | EPHA4 | 206.58 | 476.34 | 2.33 | down | 2.16e-04 |
|  | LINC00346 | 120.28 | 177.41 | 1.47 | down | 2.25e-04 |
|  | GBP5 | 1435.26 | 544.81 | 2.63 | up | 2.25e-04 |
|  | LINC01963 | 364 | 217.27 | 1.68 | up | 2.29e-04 |
|  | SRCIN1 | 174.67 | 324.14 | 1.85 | down | 2.34e-04 |
|  | CACNG6 | 11.49 | 120.01 | 10 | down | 2.38e-04 |
|  | CABCOCO1 | 58.4 | 166.93 | 2.86 | down | 2.41e-04 |
|  | CFB | 496.44 | 873.12 | 1.75 | down | 2.41e-04 |
|  | LINC00869 | 130.98 | 202.27 | 1.54 | down | 2.47e-04 |
|  | ABHD3 | 710.95 | 465.33 | 1.53 | up | 2.48e-04 |
|  | GAS6 | 1043.05 | 2223.33 | 2.13 | down | 2.51e-04 |
|  | PRF1 | 469.51 | 268.08 | 1.75 | up | 2.54e-04 |
|  | PDCD1 | 201.84 | 106.16 | 1.9 | up | 2.55e-04 |
|  | KRT5 | 2208.16 | 4100.19 | 1.85 | down | 2.59e-04 |
|  | LGALS3BP | 23124.26 | 33806.78 | 1.47 | down | 2.68e-04 |
|  | CRISP3 | 120.35 | 325.38 | 2.7 | down | 2.74e-04 |
|  | TMEM26 | 120.23 | 192.12 | 1.59 | down | 2.76e-04 |
|  | ST8SIA2 | 92.26 | 134.57 | 1.45 | down | 2.85e-04 |
|  | UAP1L1 | 1063.67 | 686.25 | 1.55 | up | 2.85e-04 |
|  | TRIB3 | 1011.91 | 599.74 | 1.69 | up | 2.90e-04 |
|  | TXNRD1 | 5311.74 | 3011.33 | 1.76 | up | 2.95e-04 |
|  | MARCO | 242.65 | 110.37 | 2.2 | up | 3.08e-04 |
|  | VWA1 | 3995.74 | 5819.08 | 1.45 | down | 3.08e-04 |
|  | GLB1L | 310.47 | 464.92 | 1.49 | down | 3.10e-04 |
|  | MKNK2 | 9942.77 | 6591.11 | 1.51 | up | 3.10e-04 |
|  | FTL | 75183.02 | 51334.64 | 1.46 | up | 3.28e-04 |
|  | WNT5B | 134.77 | 417.15 | 3.12 | down | 3.33e-04 |
|  | IL18R1 | 83.19 | 123 | 1.47 | down | 3.35e-04 |
|  | CNIH3 | 89.35 | 154.21 | 1.72 | down | 3.36e-04 |
|  | CCL4 | 313.44 | 166.42 | 1.88 | up | 3.39e-04 |
|  | MST1R | 1081.23 | 638.15 | 1.69 | up | 3.42e-04 |
|  | TRO | 241.74 | 489.55 | 2.04 | down | 3.48e-04 |
|  | CLDN5 | 337.37 | 549.93 | 1.64 | down | 3.51e-04 |
|  | GPER1 | 126.44 | 182.78 | 1.45 | down | 3.59e-04 |
|  | ACPP | 74.42 | 127.87 | 1.72 | down | 3.66e-04 |
|  | IQCN | 280.21 | 538.39 | 1.92 | down | 3.71e-04 |
|  | FOXRED2 | 2232.02 | 1540.45 | 1.45 | up | 3.74e-04 |
|  | WDR86-AS1 | 79.21 | 204.86 | 2.56 | down | 3.77e-04 |
|  | SPIRE1 | 565.21 | 276.44 | 2.04 | up | 3.79e-04 |
|  | FCGR3A | 1173 | 695.75 | 1.69 | up | 3.82e-04 |
|  | NKG7 | 757.7 | 368.14 | 2.06 | up | 3.84e-04 |
|  | DPEP1 | 115.4 | 433.43 | 3.7 | down | 3.91e-04 |
|  | EDN3 | 277.42 | 812.81 | 2.94 | down | 4.06e-04 |
|  | CD55 | 1929.86 | 2829.15 | 1.47 | down | 4.06e-04 |
|  | KALRN | 286.09 | 441.59 | 1.54 | down | 4.11e-04 |
|  | PDE1A | 61.95 | 107.74 | 1.72 | down | 4.14e-04 |
|  | IGF2 | 3774.19 | 36522.7 | 10 | down | 4.42e-04 |
|  | CST4 | 21 | 203.59 | 10 | down | 4.45e-04 |
|  | SLC51A | 110.42 | 214.94 | 1.96 | down | 4.51e-04 |
|  | ADCYAP1R1 | 382.3 | 758.58 | 2 | down | 4.66e-04 |
|  | PAX2 | 337 | 1167.69 | 3.45 | down | 4.67e-04 |
|  | CTSF | 1060.42 | 1683.99 | 1.59 | down | 4.70e-04 |
|  | SERPINA11 | 16.88 | 122.76 | 7.14 | down | 4.73e-04 |
|  | CSPG5 | 372.86 | 221.68 | 1.68 | up | 4.75e-04 |
|  | SFRP5 | 31.58 | 106.58 | 3.33 | down | 4.77e-04 |
|  | GDF15 | 5663.86 | 2756.23 | 2.05 | up | 4.78e-04 |
|  | ACACB | 236.44 | 348.01 | 1.47 | down | 4.90e-04 |
|  | C11orf88 | 26.84 | 162.97 | 6.25 | down | 4.95e-04 |
|  | SLC44A4 | 1943.05 | 3417.01 | 1.75 | down | 4.99e-04 |
|  | SAMD5 | 65.6 | 122.32 | 1.85 | down | 4.99e-04 |
|  | BTC | 113.23 | 171.41 | 1.52 | down | 5.06e-04 |
|  | NMNAT2 | 75.23 | 147.58 | 1.96 | down | 5.07e-04 |
|  | EFCAB2 | 225.37 | 361.28 | 1.61 | down | 5.07e-04 |
|  | GALNT17 | 66.72 | 169.37 | 2.56 | down | 5.16e-04 |
|  | BMP3 | 49.63 | 197.33 | 4 | down | 5.19e-04 |
|  | PCK1 | 28.84 | 268.89 | 9.09 | down | 5.24e-04 |
|  | CRLF1 | 203.16 | 517.86 | 2.56 | down | 5.31e-04 |
|  | KCNIP3 | 119.56 | 239.4 | 2 | down | 5.35e-04 |
|  | SRD5A2 | 31.51 | 146.26 | 4.55 | down | 5.36e-04 |
|  | SHE | 66.7 | 104.31 | 1.56 | down | 5.40e-04 |
|  | HEY2 | 389.91 | 736.61 | 1.89 | down | 5.46e-04 |
|  | ABLIM2 | 79.12 | 137.51 | 1.72 | down | 5.70e-04 |
|  | MDFI | 364.93 | 621.07 | 1.69 | down | 5.94e-04 |
|  | SESN2 | 1155.88 | 710.54 | 1.63 | up | 5.98e-04 |
|  | TMEM200C | 57.93 | 113.86 | 1.96 | down | 5.99e-04 |
|  | LAMC3 | 396.33 | 769.61 | 1.96 | down | 6.09e-04 |
|  | CHL1 | 171.4 | 486.97 | 2.86 | down | 6.10e-04 |
|  | ZNF750 | 152 | 227.61 | 1.49 | down | 6.35e-04 |
|  | ATOH8 | 136.16 | 213.63 | 1.56 | down | 6.38e-04 |
|  | CCDC17 | 74.19 | 413.94 | 5.56 | down | 6.40e-04 |
|  | NFE2 | 79.49 | 156.45 | 1.96 | down | 6.42e-04 |
|  | TTLL10 | 54.88 | 171.21 | 3.12 | down | 6.45e-04 |
|  | LRRC4B | 65.72 | 161.5 | 2.44 | down | 6.50e-04 |
|  | HSPA4L | 553.86 | 270.03 | 2.05 | up | 6.55e-04 |
|  | MMP12 | 764.63 | 266.66 | 2.87 | up | 6.57e-04 |
|  | NECTIN4 | 862.33 | 1342.31 | 1.56 | down | 6.67e-04 |
|  | MAB21L4 | 218.56 | 358 | 1.64 | down | 6.73e-04 |
|  | MIR205HG | 92 | 182.8 | 2 | down | 7.00e-04 |
|  | SSH2 | 1036.07 | 711.36 | 1.46 | up | 7.14e-04 |
|  | HK2 | 3445.86 | 2198.54 | 1.57 | up | 7.15e-04 |
|  | BTNL9 | 90.23 | 276.07 | 3.03 | down | 7.18e-04 |
|  | GJC2 | 78.19 | 127.61 | 1.64 | down | 7.19e-04 |
|  | PACRG | 82.88 | 205.48 | 2.5 | down | 7.51e-04 |
|  | DNAH10 | 60.84 | 183.62 | 3.03 | down | 7.54e-04 |
|  | SPAG17 | 90.88 | 246.05 | 2.7 | down | 7.56e-04 |
|  | SNTN | 27.16 | 262.32 | 10 | down | 7.62e-04 |
|  | EMCN | 105.19 | 153.37 | 1.45 | down | 7.69e-04 |
|  | PKP1 | 124.42 | 250.85 | 2 | down | 7.78e-04 |
|  | SLC52A3 | 390.28 | 654.79 | 1.67 | down | 7.79e-04 |
|  | GPR162 | 108.28 | 224.64 | 2.08 | down | 7.89e-04 |
|  | PIK3R3 | 4549.14 | 2828.77 | 1.61 | up | 8.00e-04 |
|  | STON1 | 131 | 218.05 | 1.67 | down | 8.02e-04 |
|  | TICRR | 527.37 | 359.8 | 1.47 | up | 8.02e-04 |
|  | PFKFB4 | 1009.05 | 678.78 | 1.49 | up | 8.06e-04 |
|  | SRPX | 78.35 | 113.89 | 1.45 | down | 8.26e-04 |
|  | SLC7A11 | 884 | 514.78 | 1.72 | up | 8.31e-04 |
|  | SORCS2 | 67.23 | 174.85 | 2.63 | down | 8.43e-04 |
|  | KLHL3 | 166.81 | 252.72 | 1.52 | down | 9.01e-04 |
|  | STOX2 | 159.33 | 240.35 | 1.52 | down | 9.20e-04 |
|  | FAM189A2 | 246.33 | 534.3 | 2.17 | down | 9.30e-04 |
|  | CYP2S1 | 161.88 | 351.59 | 2.17 | down | 9.35e-04 |
|  | CLIC5 | 315.4 | 662.65 | 2.08 | down | 9.39e-04 |
|  | TLE4 | 150.56 | 289.53 | 1.92 | down | 9.41e-04 |
|  | GFRA1 | 44.4 | 277.29 | 6.25 | down | 9.45e-04 |
|  | KCNG1 | 190.63 | 371.03 | 1.96 | down | 9.65e-04 |
|  | LPAR3 | 533.77 | 878.73 | 1.64 | down | 9.87e-04 |
|  | LRRN1 | 221.14 | 327.33 | 1.47 | down | 9.94e-04 |
|  | ADAMTS15 | 650.95 | 1058.42 | 1.61 | down | 9.98e-04 |
|  | DIPK1B | 784.98 | 1241.82 | 1.59 | down | 1.02e-03 |
|  | SNED1 | 297.72 | 464.32 | 1.56 | down | 1.02e-03 |
|  | TMPRSS3 | 871.49 | 1315.51 | 1.52 | down | 1.02e-03 |
|  | CALML5 | 61.63 | 153.26 | 2.5 | down | 1.06e-03 |
|  | LINC00865 | 44.42 | 111.8 | 2.5 | down | 1.07e-03 |
|  | ASS1 | 2403.84 | 4917.86 | 2.04 | down | 1.07e-03 |
|  | SLC38A3 | 53.53 | 117.74 | 2.22 | down | 1.08e-03 |
|  | ERG | 205.51 | 304.84 | 1.49 | down | 1.08e-03 |
|  | TENM4 | 240.6 | 639.6 | 2.63 | down | 1.08e-03 |
|  | CA13 | 91.56 | 135.65 | 1.49 | down | 1.08e-03 |
|  | ADD2 | 206.02 | 337.67 | 1.64 | down | 1.10e-03 |
|  | KLK11 | 620.07 | 1313.44 | 2.13 | down | 1.11e-03 |
|  | LYNX1 | 298.26 | 693.13 | 2.33 | down | 1.12e-03 |
|  | C6 | 9.19 | 145.03 | 16.67 | down | 1.12e-03 |
|  | SLC25A27 | 125.88 | 208.45 | 1.67 | down | 1.13e-03 |
|  | GINS4 | 452.67 | 310.03 | 1.46 | up | 1.14e-03 |
|  | KCNJ15 | 90 | 283.33 | 3.12 | down | 1.16e-03 |
|  | MMRN1 | 117.28 | 214.73 | 1.82 | down | 1.17e-03 |
|  | MINDY1 | 433.93 | 628.06 | 1.45 | down | 1.17e-03 |
|  | ENPP2 | 712.77 | 1183.49 | 1.67 | down | 1.17e-03 |
|  | HMCN2 | 75.88 | 138.66 | 1.82 | down | 1.21e-03 |
|  | CCDC33 | 94.07 | 188.26 | 2 | down | 1.21e-03 |
|  | KCNN3 | 120.05 | 234.03 | 1.96 | down | 1.21e-03 |
|  | ERI1 | 969.86 | 666.67 | 1.45 | up | 1.22e-03 |
|  | KNL1 | 633.14 | 412.71 | 1.53 | up | 1.23e-03 |
|  | SUSD4 | 221.21 | 437.9 | 1.96 | down | 1.26e-03 |
|  | ZMYND12 | 80.26 | 155.19 | 1.92 | down | 1.26e-03 |
|  | COLQ | 71.09 | 111.69 | 1.56 | down | 1.26e-03 |
|  | SYN2 | 73.16 | 136.24 | 1.85 | down | 1.28e-03 |
|  | CD2 | 513.02 | 301.47 | 1.7 | up | 1.29e-03 |
|  | TCHH | 34.88 | 119.17 | 3.45 | down | 1.30e-03 |
|  | ARHGAP40 | 75.77 | 153.16 | 2.04 | down | 1.31e-03 |
|  | ADAMTSL3 | 66.77 | 151.4 | 2.27 | down | 1.31e-03 |
|  | MINDY4 | 104.42 | 151.37 | 1.45 | down | 1.36e-03 |
|  | AMBP | 888.09 | 245.42 | 3.62 | up | 1.37e-03 |
|  | RTP4 | 199.3 | 299.65 | 1.49 | down | 1.39e-03 |
|  | IGFBP4 | 6873.12 | 10698.94 | 1.56 | down | 1.39e-03 |
|  | ADRA2C | 525.93 | 801.68 | 1.52 | down | 1.39e-03 |
|  | PPARGC1A | 24 | 108.54 | 4.55 | down | 1.42e-03 |
|  | CALHM6 | 222.28 | 146.47 | 1.52 | up | 1.44e-03 |
|  | SLC16A4 | 94.23 | 155.1 | 1.64 | down | 1.44e-03 |
|  | GUCY1A2 | 129.93 | 233.05 | 1.79 | down | 1.49e-03 |
|  | C2orf40 | 79.02 | 137.86 | 1.75 | down | 1.51e-03 |
|  | SDK2 | 220.81 | 536.31 | 2.44 | down | 1.52e-03 |
|  | DMBT1 | 335.35 | 991.34 | 2.94 | down | 1.53e-03 |
|  | SOX9 | 1678.23 | 2626.41 | 1.56 | down | 1.53e-03 |
|  | ADGRB2 | 243.16 | 351.73 | 1.45 | down | 1.54e-03 |
|  | LAYN | 96.47 | 148.41 | 1.54 | down | 1.55e-03 |
|  | PRKAR2B | 114.14 | 208.29 | 1.82 | down | 1.55e-03 |
|  | GARNL3 | 85.74 | 124.12 | 1.45 | down | 1.56e-03 |
|  | KCNJ2 | 83.12 | 131.73 | 1.59 | down | 1.57e-03 |
|  | LYZ | 2244.67 | 1284.46 | 1.75 | up | 1.58e-03 |
|  | CD79A | 406.21 | 212.9 | 1.91 | up | 1.59e-03 |
|  | NOL4L | 860.51 | 1280.28 | 1.49 | down | 1.59e-03 |
|  | C1orf115 | 757.42 | 1102.04 | 1.45 | down | 1.60e-03 |
|  | C3 | 15743.35 | 26775.15 | 1.69 | down | 1.60e-03 |
|  | KLF12 | 260.65 | 405.58 | 1.56 | down | 1.63e-03 |
|  | ANKRD66 | 22.65 | 113.05 | 5 | down | 1.73e-03 |
|  | EBF4 | 284.49 | 524.28 | 1.85 | down | 1.73e-03 |
|  | OPN3 | 115.19 | 193.72 | 1.69 | down | 1.74e-03 |
|  | CCL5 | 2101.91 | 969.81 | 2.17 | up | 1.76e-03 |
|  | NOVA2 | 74.19 | 123.44 | 1.67 | down | 1.76e-03 |
|  | NELL1 | 58.12 | 104.01 | 1.79 | down | 1.77e-03 |
|  | ZSWIM4 | 824.65 | 1219.04 | 1.47 | down | 1.78e-03 |
|  | IMPA2 | 1822 | 1097.41 | 1.66 | up | 1.79e-03 |
|  | CRTAC1 | 39.19 | 190.33 | 4.76 | down | 1.80e-03 |
|  | COL13A1 | 49 | 160.83 | 3.33 | down | 1.81e-03 |
|  | BMP6 | 66.7 | 129.99 | 1.96 | down | 1.81e-03 |
|  | ANXA2P2 | 209.56 | 143.66 | 1.46 | up | 1.82e-03 |
|  | SLC25A15 | 316.12 | 194.4 | 1.63 | up | 1.83e-03 |
|  | FSTL4 | 68.4 | 152.39 | 2.22 | down | 1.87e-03 |
|  | PCSK1N | 329.19 | 770.11 | 2.33 | down | 1.88e-03 |
|  | CCDC187 | 51.28 | 116.22 | 2.27 | down | 1.89e-03 |
|  | LY6E | 8941.12 | 13449.53 | 1.52 | down | 1.91e-03 |
|  | ERP27 | 176.3 | 386.85 | 2.17 | down | 1.91e-03 |
|  | SLPI | 18196.09 | 28648.64 | 1.56 | down | 1.92e-03 |
|  | WNT10A | 123.95 | 330.17 | 2.63 | down | 1.93e-03 |
|  | MACC1 | 276.37 | 484.84 | 1.75 | down | 1.93e-03 |
|  | GABRE | 110.88 | 243.93 | 2.22 | down | 1.93e-03 |
|  | CUBN | 99 | 159.94 | 1.61 | down | 1.97e-03 |
|  | EN2 | 396.63 | 195.78 | 2.03 | up | 2.00e-03 |
|  | CMPK2 | 174.93 | 277.81 | 1.59 | down | 2.01e-03 |
|  | ASTN1 | 73.79 | 168.28 | 2.27 | down | 2.04e-03 |
|  | C1orf53 | 80.47 | 119.33 | 1.49 | down | 2.05e-03 |
|  | ANKRD45 | 60.74 | 107.39 | 1.75 | down | 2.05e-03 |
|  | TTC30B | 265.21 | 387.94 | 1.47 | down | 2.08e-03 |
|  | CASC15 | 77.88 | 139.47 | 1.79 | down | 2.09e-03 |
|  | FAM3B | 203.65 | 365.89 | 1.79 | down | 2.09e-03 |
|  | CNTN3 | 101.53 | 156.46 | 1.54 | down | 2.10e-03 |
|  | FHOD3 | 120.84 | 192.18 | 1.59 | down | 2.10e-03 |
|  | DUSP15 | 58.21 | 106.49 | 1.82 | down | 2.18e-03 |
|  | WNT2B | 61.65 | 130.69 | 2.13 | down | 2.19e-03 |
|  | SLC26A7 | 108.26 | 311.74 | 2.86 | down | 2.24e-03 |
|  | CRABP2 | 1844.35 | 3719.25 | 2 | down | 2.24e-03 |
|  | FOXO6 | 105.44 | 205.92 | 1.96 | down | 2.27e-03 |
|  | DNAH2 | 157.51 | 359.04 | 2.27 | down | 2.30e-03 |
|  | CXCR3 | 220.51 | 123.78 | 1.78 | up | 2.30e-03 |
|  | PSCA | 61.88 | 270.51 | 4.35 | down | 2.39e-03 |
|  | CFAP65 | 112.7 | 326.02 | 2.86 | down | 2.39e-03 |
|  | CPXM1 | 1243.63 | 1985.88 | 1.59 | down | 2.42e-03 |
|  | TSPOAP1 | 521.63 | 761.67 | 1.47 | down | 2.43e-03 |
|  | DNAH17 | 73.6 | 110.63 | 1.49 | down | 2.52e-03 |
|  | PTN | 491.51 | 779.33 | 1.59 | down | 2.53e-03 |
|  | LRRC10B | 141.07 | 361.06 | 2.56 | down | 2.56e-03 |
|  | PNMA3 | 90.88 | 276.51 | 3.03 | down | 2.57e-03 |
|  | SAMD11 | 323.74 | 557.59 | 1.72 | down | 2.58e-03 |
|  | MX1 | 3212.58 | 4774.36 | 1.49 | down | 2.64e-03 |
|  | CYP39A1 | 113.84 | 175.08 | 1.54 | down | 2.64e-03 |
|  | CHAC1 | 450.63 | 305.88 | 1.47 | up | 2.70e-03 |
|  | ERICH3 | 163.16 | 351.29 | 2.17 | down | 2.72e-03 |
|  | FAM3D | 25.88 | 138.7 | 5.26 | down | 2.72e-03 |
|  | BEX4 | 750.35 | 1136.39 | 1.52 | down | 2.73e-03 |
|  | KRT6B | 31.19 | 183.69 | 5.88 | down | 2.75e-03 |
|  | FTH1 | 17909.3 | 11745.2 | 1.52 | up | 2.78e-03 |
|  | EFCAB1 | 196.74 | 475.74 | 2.44 | down | 2.79e-03 |
|  | LINC01503 | 154.58 | 266.95 | 1.72 | down | 2.81e-03 |
|  | F13A1 | 243.7 | 367.58 | 1.52 | down | 2.83e-03 |
|  | ADGRG2 | 225.37 | 535.7 | 2.38 | down | 2.83e-03 |
|  | SST | 323.23 | 1188.23 | 3.7 | down | 2.84e-03 |
|  | PSAT1 | 3750.98 | 2599.82 | 1.44 | up | 2.85e-03 |
|  | PLEKHS1 | 522.47 | 1429.18 | 2.7 | down | 2.94e-03 |
|  | PTPRJ | 1610.37 | 1103.42 | 1.46 | up | 2.94e-03 |
|  | TMEM220 | 97.09 | 139.83 | 1.45 | down | 2.98e-03 |
|  | HR | 158.47 | 234.09 | 1.47 | down | 3.00e-03 |
|  | PHLDA2 | 896.79 | 473.34 | 1.89 | up | 3.09e-03 |
|  | C1QB | 4513.02 | 2873.69 | 1.57 | up | 3.09e-03 |
|  | MB21D2 | 106 | 190.19 | 1.79 | down | 3.10e-03 |
|  | NPR1 | 539.23 | 854.56 | 1.59 | down | 3.13e-03 |
|  | CFAP44 | 329.72 | 488.66 | 1.49 | down | 3.13e-03 |
|  | MAGEH1 | 137.98 | 263.79 | 1.92 | down | 3.18e-03 |
|  | CD27 | 193.91 | 116.31 | 1.67 | up | 3.24e-03 |
|  | USP51 | 76.05 | 118.68 | 1.56 | down | 3.33e-03 |
|  | SSPN | 177.49 | 282.34 | 1.59 | down | 3.35e-03 |
|  | RFXAP | 208.72 | 143.01 | 1.46 | up | 3.40e-03 |
|  | CLIP3 | 420.12 | 623.42 | 1.49 | down | 3.41e-03 |
|  | DTNA | 80.28 | 157.63 | 1.96 | down | 3.44e-03 |
|  | CALN1 | 73.44 | 110.16 | 1.49 | down | 3.44e-03 |
|  | C11orf16 | 58.6 | 128.81 | 2.22 | down | 3.46e-03 |
|  | TTC30A | 246.37 | 375.91 | 1.52 | down | 3.52e-03 |
|  | FAM155A | 100.05 | 188.56 | 1.89 | down | 3.55e-03 |
|  | NPR3 | 147.51 | 348.61 | 2.38 | down | 3.56e-03 |
|  | HEXIM1 | 3136.84 | 2063.87 | 1.52 | up | 3.60e-03 |
|  | DISC1 | 196.88 | 294.21 | 1.49 | down | 3.65e-03 |
|  | LTBP2 | 1092.91 | 1806.33 | 1.64 | down | 3.70e-03 |
|  | KLK8 | 81.28 | 215.24 | 2.63 | down | 3.73e-03 |
|  | CCDC151 | 97.21 | 217.9 | 2.22 | down | 3.75e-03 |
|  | TTC22 | 216.14 | 332.75 | 1.54 | down | 3.79e-03 |
|  | FCGBP | 1582.02 | 2626.34 | 1.67 | down | 3.81e-03 |
|  | MYH7B | 146.93 | 864.28 | 5.88 | down | 3.85e-03 |
|  | IL34 | 61.4 | 104.39 | 1.69 | down | 3.88e-03 |
|  | PTCH1 | 544.47 | 822.9 | 1.52 | down | 3.89e-03 |
|  | ANXA1 | 17701.26 | 8509.77 | 2.08 | up | 3.89e-03 |
|  | APOB | 3.26 | 117.66 | 33.33 | down | 3.91e-03 |
|  | ZBED6CL | 641.93 | 425.24 | 1.51 | up | 3.92e-03 |
|  | CLDN23 | 112.7 | 168.8 | 1.49 | down | 4.00e-03 |
|  | FXYD3 | 1788.12 | 2834.18 | 1.59 | down | 4.04e-03 |
|  | EFNB2 | 822.44 | 1323.7 | 1.61 | down | 4.08e-03 |
|  | TSPAN7 | 356.14 | 762.84 | 2.13 | down | 4.12e-03 |
|  | THBD | 190.65 | 354.82 | 1.85 | down | 4.14e-03 |
|  | SOBP | 106.07 | 207.67 | 1.96 | down | 4.19e-03 |
|  | OLFM1 | 1008.14 | 163.06 | 6.18 | up | 4.21e-03 |
|  | CDKN1A | 6380.37 | 4345.73 | 1.47 | up | 4.21e-03 |
|  | SMOX | 1086.88 | 752.87 | 1.44 | up | 4.24e-03 |
|  | EFCAB12 | 140.49 | 273.23 | 1.96 | down | 4.24e-03 |
|  | PLSCR4 | 87.91 | 140.53 | 1.59 | down | 4.26e-03 |
|  | LRP2 | 84.84 | 365.68 | 4.35 | down | 4.28e-03 |
|  | TRABD2A | 51.91 | 106.11 | 2.04 | down | 4.33e-03 |
|  | CCDC153 | 125.86 | 196.37 | 1.56 | down | 4.36e-03 |
|  | TFRC | 7750.4 | 5213.55 | 1.49 | up | 4.44e-03 |
|  | STOML3 | 44.53 | 153.16 | 3.45 | down | 4.46e-03 |
|  | CAPN5 | 514.93 | 756.27 | 1.47 | down | 4.46e-03 |
|  | GAS7 | 633.65 | 923.91 | 1.45 | down | 4.49e-03 |
|  | PANX2 | 397.58 | 219.81 | 1.81 | up | 4.55e-03 |
|  | MZB1 | 586 | 379.41 | 1.54 | up | 4.55e-03 |
|  | ADM | 2224.93 | 1519.69 | 1.46 | up | 4.62e-03 |
|  | CD109 | 336.67 | 657.84 | 1.96 | down | 4.63e-03 |
|  | FABP6 | 89.93 | 183.37 | 2.04 | down | 4.66e-03 |
|  | PAM | 6988.88 | 10282.99 | 1.47 | down | 4.66e-03 |
|  | MYO3B | 114.09 | 216.12 | 1.89 | down | 4.71e-03 |
|  | MME | 144.72 | 285.8 | 1.96 | down | 4.71e-03 |
|  | CD3D | 328.51 | 191.34 | 1.72 | up | 4.74e-03 |
|  | PORCN | 812.47 | 1301.32 | 1.61 | down | 4.76e-03 |
|  | APOA1 | 22.37 | 553.37 | 25 | down | 4.76e-03 |
|  | PLPPR3 | 43.58 | 180.73 | 4.17 | down | 4.78e-03 |
|  | ALDH3A2 | 4866.86 | 3273.63 | 1.49 | up | 4.80e-03 |
|  | CCR5 | 281.28 | 155.24 | 1.81 | up | 4.81e-03 |
|  | ESCO2 | 348.88 | 228.08 | 1.53 | up | 4.81e-03 |
|  | ENTPD3 | 366.88 | 652.78 | 1.79 | down | 4.85e-03 |
|  | DCHS2 | 82.63 | 178.14 | 2.17 | down | 4.87e-03 |
|  | KRT13 | 210.42 | 324.14 | 1.54 | down | 4.91e-03 |
|  | HLA-J | 82.26 | 148.54 | 1.82 | down | 4.92e-03 |
|  | ERV3-1 | 325 | 489.34 | 1.52 | down | 4.97e-03 |
|  | LRRC71 | 79.7 | 242.09 | 3.03 | down | 5.07e-03 |
|  | KCNK5 | 541.14 | 792.78 | 1.47 | down | 5.08e-03 |
|  | KIT | 141.28 | 211.12 | 1.49 | down | 5.11e-03 |
|  | CXXC4 | 81.35 | 143.69 | 1.75 | down | 5.12e-03 |
|  | SCIN | 182.98 | 272.09 | 1.49 | down | 5.14e-03 |
|  | CACNA1A | 79.88 | 145.52 | 1.82 | down | 5.18e-03 |
|  | SYT7 | 294.14 | 507.58 | 1.72 | down | 5.25e-03 |
|  | CFH | 609.42 | 1183.67 | 1.96 | down | 5.26e-03 |
|  | HAS3 | 736.4 | 371.81 | 1.98 | up | 5.34e-03 |
|  | DYDC2 | 76.67 | 222.71 | 2.94 | down | 5.34e-03 |
|  | CFAP300 | 109.19 | 236.91 | 2.17 | down | 5.37e-03 |
|  | PDE8B | 120.7 | 176.69 | 1.47 | down | 5.39e-03 |
|  | CCL18 | 544 | 281.52 | 1.93 | up | 5.43e-03 |
|  | NR5A2 | 177.7 | 287.46 | 1.61 | down | 5.43e-03 |
|  | IGSF1 | 25.3 | 214.83 | 8.33 | down | 5.45e-03 |
|  | WT1 | 121.47 | 452.69 | 3.7 | down | 5.49e-03 |
|  | HK3 | 173.7 | 118.33 | 1.47 | up | 5.52e-03 |
|  | CMBL | 1293.07 | 889.7 | 1.45 | up | 5.58e-03 |
|  | CDH23 | 83.86 | 167.09 | 2 | down | 5.59e-03 |
|  | DZIP1L | 78.09 | 171.07 | 2.17 | down | 5.61e-03 |
|  | IFI27 | 12159.53 | 18430.28 | 1.52 | down | 5.62e-03 |
|  | HEY1 | 352.47 | 531.15 | 1.52 | down | 5.71e-03 |
|  | SAMD9 | 290.84 | 502.01 | 1.72 | down | 5.83e-03 |
|  | TENT5A | 346.33 | 503.09 | 1.45 | down | 5.84e-03 |
|  | S100A7 | 88.09 | 207.71 | 2.38 | down | 5.90e-03 |
|  | CCDC74A | 364.86 | 591.49 | 1.61 | down | 5.95e-03 |
|  | PIANP | 91.98 | 136.28 | 1.49 | down | 6.04e-03 |
|  | VSIG8 | 57.12 | 121.15 | 2.13 | down | 6.05e-03 |
|  | CLSTN2 | 188.81 | 421.49 | 2.22 | down | 6.07e-03 |
|  | PI3 | 1004.58 | 1809.29 | 1.79 | down | 6.08e-03 |
|  | ADAMTSL2 | 227.42 | 547.57 | 2.38 | down | 6.22e-03 |
|  | ORM1 | 54.56 | 273.7 | 5 | down | 6.26e-03 |
|  | MAATS1 | 172.63 | 316.77 | 1.85 | down | 6.29e-03 |
|  | C6orf15 | 37.26 | 336.08 | 9.09 | down | 6.35e-03 |
|  | GREM1 | 216.02 | 145.05 | 1.49 | up | 6.45e-03 |
|  | TMEM246 | 104.21 | 197.72 | 1.89 | down | 6.53e-03 |
|  | TTLL9 | 52.28 | 108.75 | 2.08 | down | 6.66e-03 |
|  | MAPK10 | 157.05 | 234.14 | 1.49 | down | 6.68e-03 |
|  | SYT5 | 62.35 | 159.32 | 2.56 | down | 6.71e-03 |
|  | CENPK | 318.26 | 217.74 | 1.46 | up | 6.91e-03 |
|  | MXRA5 | 1228.6 | 2015.51 | 1.64 | down | 6.97e-03 |
|  | IL11RA | 149.88 | 247.26 | 1.64 | down | 7.08e-03 |
|  | VWA3A | 190.02 | 455.01 | 2.38 | down | 7.11e-03 |
|  | HOXD10 | 159.93 | 255.82 | 1.59 | down | 7.16e-03 |
|  | LOC100507053 | 73.23 | 120.82 | 1.64 | down | 7.18e-03 |
|  | SIX3 | 44.7 | 109.86 | 2.44 | down | 7.18e-03 |
|  | LILRB4 | 545.47 | 347.21 | 1.57 | up | 7.37e-03 |
|  | ANKRD65 | 586.37 | 932.33 | 1.59 | down | 7.45e-03 |
|  | NOV | 314.33 | 587.07 | 1.85 | down | 7.45e-03 |
|  | KCNMB1 | 218.67 | 150.69 | 1.45 | up | 7.48e-03 |
|  | SULF2 | 2300.77 | 3599.62 | 1.56 | down | 7.74e-03 |
|  | GFOD1 | 244.19 | 358.75 | 1.47 | down | 7.79e-03 |
|  | FMN2 | 43.84 | 117.26 | 2.7 | down | 7.83e-03 |
|  | MN1 | 70.09 | 150.26 | 2.13 | down | 7.85e-03 |
|  | RNF152 | 412.86 | 656.86 | 1.59 | down | 7.95e-03 |
|  | ACKR1 | 258.84 | 392.15 | 1.52 | down | 7.98e-03 |
|  | APCDD1 | 3704.28 | 6364.06 | 1.72 | down | 8.06e-03 |
|  | CST6 | 90.16 | 202.41 | 2.22 | down | 8.28e-03 |
|  | RRAD | 246.28 | 644.52 | 2.63 | down | 8.31e-03 |
|  | SPEG | 246.56 | 405.63 | 1.64 | down | 8.34e-03 |
|  | RUNX3 | 391.79 | 668.2 | 1.69 | down | 8.38e-03 |
|  | RHOV | 364.67 | 542.07 | 1.49 | down | 8.43e-03 |
|  | STEAP4 | 64.63 | 161.73 | 2.5 | down | 8.46e-03 |
|  | CD3E | 750.84 | 418.27 | 1.8 | up | 8.60e-03 |
|  | KLK10 | 1123.47 | 1678.2 | 1.49 | down | 8.64e-03 |
|  | MAPK12 | 712.74 | 370.55 | 1.92 | up | 8.66e-03 |
|  | SLC7A5 | 6246.65 | 4163.74 | 1.5 | up | 8.68e-03 |
|  | CRYAB | 1957.86 | 1273.74 | 1.54 | up | 8.72e-03 |
|  | FOXS1 | 78.26 | 159.27 | 2.04 | down | 8.86e-03 |
|  | ZSCAN31 | 671.12 | 427.69 | 1.57 | up | 8.88e-03 |
|  | LRP8 | 692.49 | 473.78 | 1.46 | up | 8.99e-03 |
|  | RNF182 | 64.74 | 130.04 | 2 | down | 9.05e-03 |
|  | LYPD3 | 208.7 | 378.54 | 1.82 | down | 9.06e-03 |
|  | OXCT1 | 923.74 | 639.22 | 1.45 | up | 9.06e-03 |
|  | CTSE | 76.84 | 182.27 | 2.38 | down | 9.08e-03 |
|  | PRR29 | 187 | 369.88 | 1.96 | down | 9.11e-03 |
|  | KRT6A | 767.07 | 1644.24 | 2.13 | down | 9.14e-03 |
|  | PPP1R14A | 239.07 | 392.12 | 1.64 | down | 9.14e-03 |
|  | CFTR | 332.67 | 636.65 | 1.92 | down | 9.22e-03 |
|  | ZNF516 | 1382.05 | 2164.55 | 1.56 | down | 9.24e-03 |
|  | CC2D2A | 389.7 | 591.51 | 1.52 | down | 9.35e-03 |
|  | HIF3A | 58.88 | 341.33 | 5.88 | down | 9.43e-03 |
|  | ERICH2 | 78.95 | 121.75 | 1.54 | down | 9.47e-03 |
|  | APOD | 419.14 | 765.49 | 1.82 | down | 9.47e-03 |
|  | RASL11B | 229.09 | 489.59 | 2.13 | down | 9.54e-03 |
|  | HAVCR2 | 367.79 | 233.96 | 1.57 | up | 9.63e-03 |
|  | NHLRC4 | 100.56 | 173.83 | 1.72 | down | 9.76e-03 |
|  | HOXD9 | 307.16 | 450.17 | 1.47 | down | 1.00e-02 |
