# Supplementary figures and images for: R-spondin family biology and emerging linkages to cancer
Source: Ann Med. 2023 Jan 16;55(1):428–46. doi: 10.1080/07853890.2023.2166981 (PMC9848353; doi:10.1080/07853890.2023.2166981)

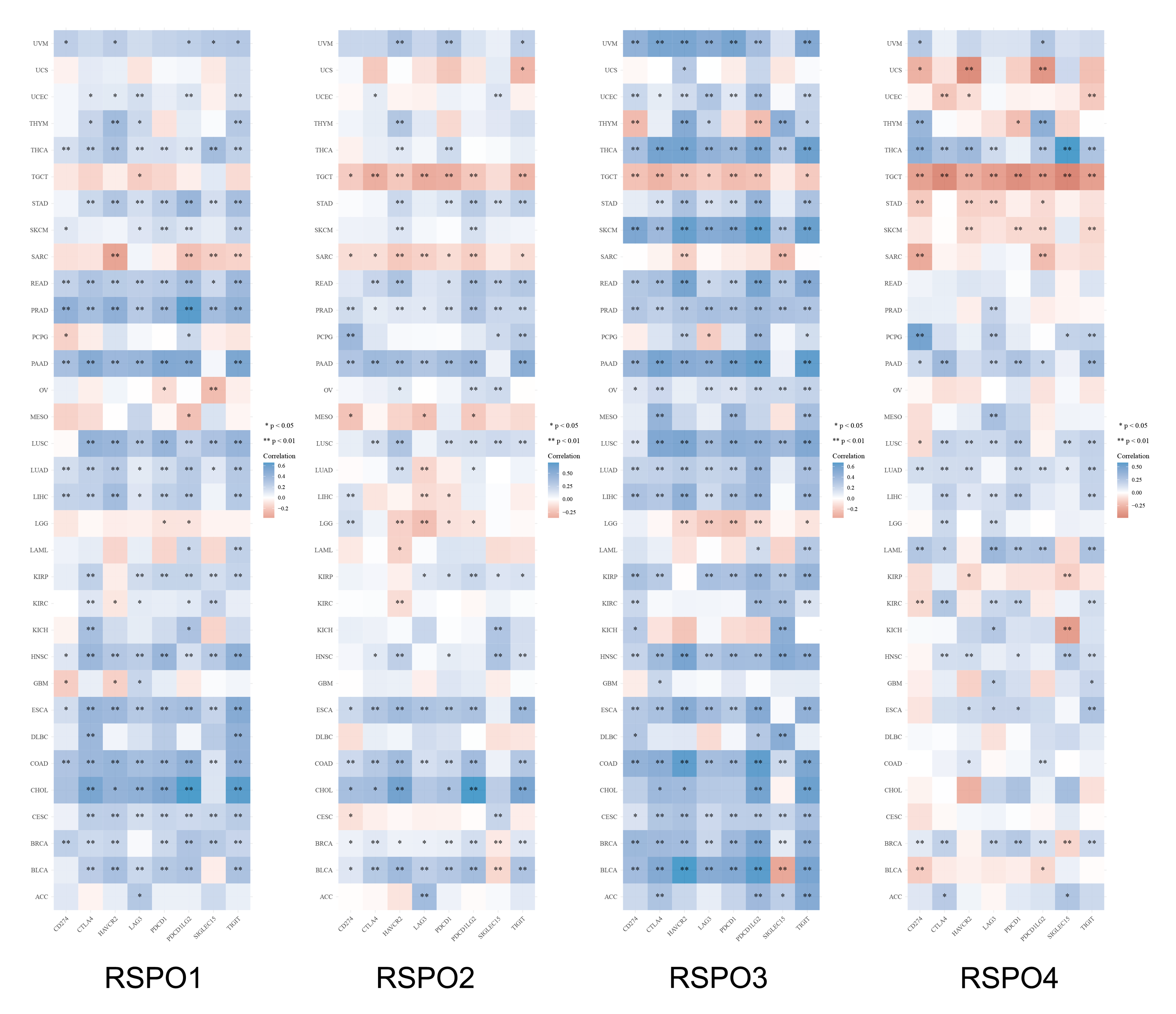

Supplement: Supplemental Material [file IANN_A_2166981_SM6674.tif]

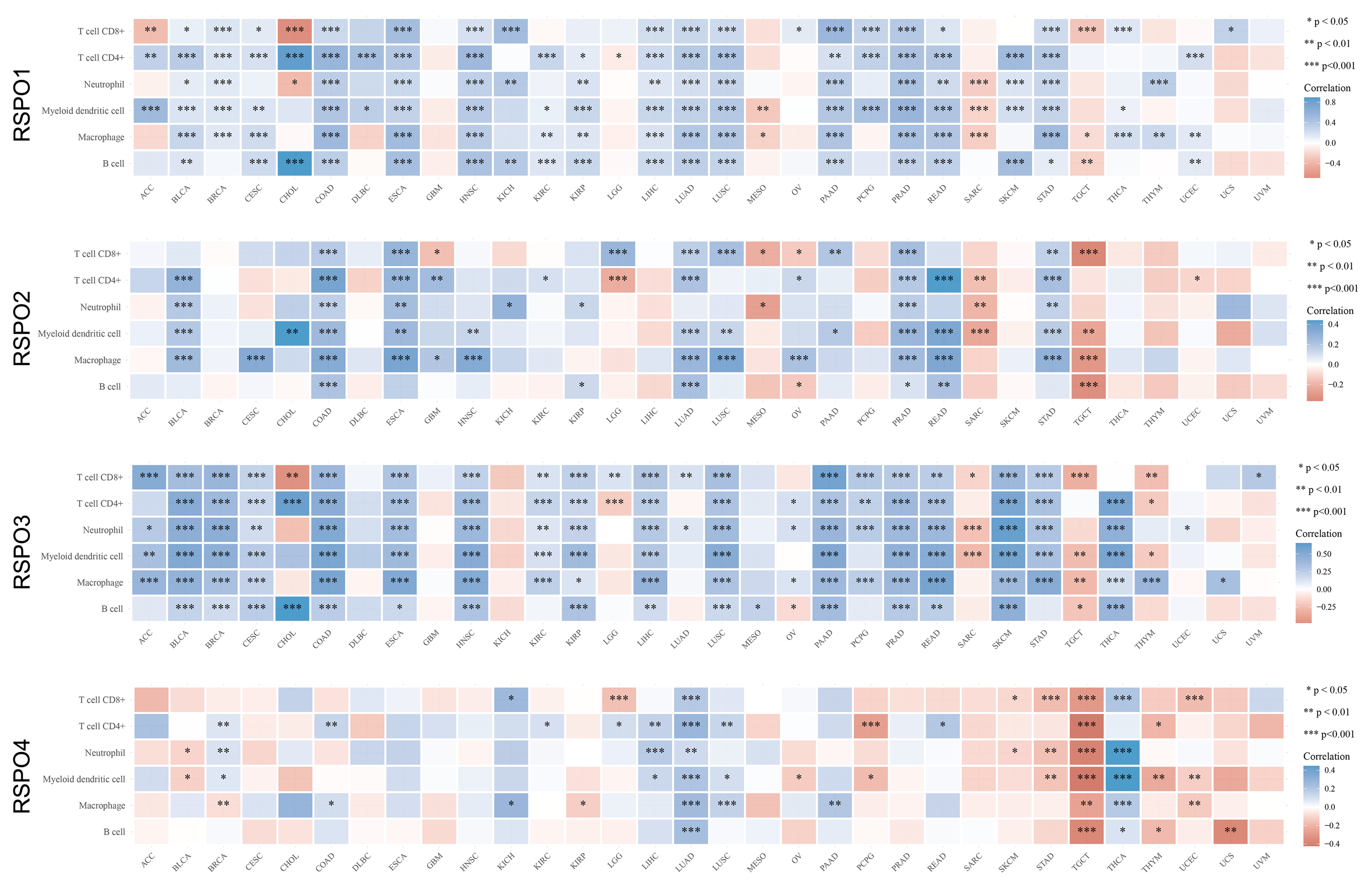

Supplement: Supplemental Material [file IANN_A_2166981_SM6658.tif]
